# Comparing Effectiveness and Safety of SGLT2 Inhibitors vs DPP-4 Inhibitors in Patients With Type 2 Diabetes and Varying Baseline HbA_1c_ Levels

**DOI:** 10.1001/jamainternmed.2022.6664

**Published:** 2023-02-06

**Authors:** Elvira D’Andrea, Deborah J. Wexler, Seoyoung C. Kim, Julie M. Paik, Ethan Alt, Elisabetta Patorno

**Affiliations:** 1 Division of Pharmacoepidemiology and Pharmacoeconomics; Brigham and Women’s Hospital and Harvard Medical School, Boston, Massachusetts; 2 Diabetes Center, Massachusetts General Hospital, Harvard Medical School, Boston; 3 Division of Kidney Medicine, Brigham and Women’s Hospital, Boston, Massachusetts; 4 New England Geriatric Research Education and Clinical Center, VA Boston Healthcare System, Boston, Massachusetts

## Abstract

**Importance:**

Sodium-glucose cotransporter 2 inhibitor (SGLT2i) therapy has been associated with cardiovascular benefits and a few adverse events; however, whether the comparative effectiveness and safety profiles vary with differences in baseline hemoglobin A_1c_ (HbA_1c_) levels is unknown.

**Objective:**

To compare cardiovascular effectiveness and safety of treatment with SGLT2i vs dipeptidyl peptidase 4 inhibitor (DPP-4i) in adults with type 2 diabetes (T2D) (1) overall and (2) at varying baseline HbA_1c_ levels.

**Design, Setting, and Participants:**

A new-user comparative effectiveness and safety research study was conducted among 144 614 commercially insured adults, initiating treatment with SGLT2i or DPP-4i and with a recorded T2D diagnosis at baseline and at least 1 HbA_1c_ laboratory result recorded within 3 months before treatment initiation.

**Interventions:**

The intervention consisted of the initiation of treatment with SGLT2i or DPP-4i.

**Main Outcomes and Measures:**

Primary outcomes were a composite of myocardial infarction, stroke, or all-cause death (modified major adverse cardiovascular events [MACE]) and hospitalization for heart failure (HHF). Safety outcomes were hypovolemia, fractures, falls, genital infections, diabetic ketoacidosis (DKA), acute kidney injury (AKI), and lower-limb amputation. Incidence rate (IR) per 1000 person-years, hazard ratios (HR) and rate differences (RD) with their 95% CIs were estimated controlling for 128 covariates.

**Results:**

A total of 144 614 eligible adults (mean [SD] age, 62 [12.4] years; 54% male participants) with T2D initiating treatment with a SGLT2i (n = 60 523) or a DPP-4i (n = 84 091) were identified; 44 099 had an HbA_1c_ baseline value of less than 7.5%, 52 986 between 7.5% and 9%, and 47 529 greater than 9%. Overall, 87 274 eligible patients were 1:1 propensity score–matched: 24 052 with HbA_1c_ less than 7.5%; 32 290 with HbA_1c_ between 7.5% and 9%; and 30 932 with HbA_1c_ greater than 9% (to convert percentage of total hemoglobin to proportion of total hemoglobin, multiply by 0.01). The initiation of SGLT2i vs DPP-4i was associated with a reduction in the risk of modified MACE (IR per 1000 person-years 17.13 vs 20.18, respectively; HR, 0.85; 95% CI, 0.75-0.95; RD, −3.02; 95% CI, −5.23 to –0.80) and HHF (IR per 1000 person-years 3.68 vs 8.08, respectively; HR, 0.46; 95% CI, 0.35 to 0.57; RD −4.37; 95% CI, −5.62 to −3.12) over a mean follow-up of 8 months, with no evidence of treatment effect heterogeneity across the HbA_1c_ levels. Treatment with SGLT2i showed an increased risk of genital infections and DKA and a reduced AKI risk compared with DPP-4i. Findings were consistent by HbA_1c_ levels, except for a more pronounced risk of genital infections associated with SGLT2i for HbA_1c_ levels of 7.5% to 9% (IR per 1000 person-years 68.5 vs 22.8, respectively; HR, 3.10; 95% CI, 2.68-3.58; RD, 46.22; 95% CI, 40.54-51.90).

**Conclusions and Relevance:**

In this comparative effectiveness and safety research study among adults with T2D, SGLT2i vs DPP-4i treatment initiators had a reduced risk of modified MACE and HHF, an increased risk of genital infections and DKA, and a lower risk of AKI, regardless of baseline HbA_1c_.

## Key Points

**Question:**

Does the effectiveness and safety of sodium-glucose cotransporter 2 inhibitors (SGLT2i) differ from that of dipeptidyl peptidase 4 inhibitors (DPP-4i) in patients with type 2 diabetes (T2D) overall and at varying baseline hemoglobin A_1c_ (HbA_1c_) levels?

**Findings:**

In this large new-user comparative effectiveness and safety research study including 87 274 propensity-scored matched adults with T2D, SGLT2i treatment initiators had a reduced risk of major cardiovascular events, heart failure, and acute kidney injury and an increased risk of genital infections and diabetic ketoacidosis compared with DPP-4i treatment initiators, regardless of their baseline HbA_1c_ level.

**Meaning:**

Despite concern that use of SGLT2i at higher HbA_1c_ levels would cause excess risk, the findings of this study suggest that patients with T2D can benefit from the use of SGLT2i regardless of glycemic control, with the expected adverse effect profile when compared with DPP-4i, with no additional risk of adverse effects in patients with elevated HbA_1c_ levels.

## Teaser

This comparative effectiveness study evaluates cardiovascular effectiveness and safety of SGLT2 inhibitors vs DPP-4 inhibitors in adults with type 2 diabetes overall and at varying baseline HbA_1c_ levels.

## Introduction

Type 2 diabetes (T2D) affects more than 11% of the US population and is associated with increased morbidity and mortality from cardiovascular and kidney disease.^1,2^ Mitigating the risk of these complications is a priority in the management of diabetes. In large-scale postmarketing randomized cardiovascular outcome trials (CVOTs), sodium-glucose cotransporter 2 inhibitors (SGLT2i) have demonstrated a cardiorenal protective effect in patients with T2D and established cardiovascular or kidney conditions.^3,4,5,6^ These benefits have been reproduced in real-world evidence studies, which encompass a patient population with a broader spectrum of cardiovascular risk as seen in clinical practice.^7,8^ However, it is still unclear whether patients with different levels of hyperglycemia can similarly benefit from the use of SGLT2i.

Cardiovascular outcome trials of SGLT2i have explored potential for treatment effect heterogeneity by hyperglycemia, defined as baseline assessment of glycated hemoglobin (HbA_1c_), identifying some potential variation in the cardiovascular effects of SGLT2i by HbA_1c_ level.^3,4,5,6,9^ However, subgroup analyses within CVOTs are generally underpowered to detect meaningful differences,^9^ and patients with uncontrolled diabetes were often underrepresented due to strict inclusion criteria.^3,4,6^ Further, since SGLT2i induce a glycosuric response by reducing kidney tubular glucose reabsorption, those medications can have a more pronounced effect on hyperglycemia in patients with poor glycemic control due to the increased amount of filtered glucose.^10^ The accompanying diuretic and natriuretic effect of SGLT2 inhibition may lead to a more marked improvement in volume status in patients with elevated vs controlled glycemia resulting in a lower risk for heart failure. Conversely, the higher concentration of glucose in the urine in patients with severe hyperglycemia could lead to an increased risk of adverse effects such as mycotic infections, volume depletion due to the osmotic diuresis induced by glycosuria, consequent increased risk of falls and fractures, and diabetic ketoacidosis (DKA).^11^

In this large comparative effectiveness and safety research study of patients with T2D, we evaluated cardiovascular and safety events associated with the initiation of SGLT2i treatment compared with dipeptidyl peptidase 4 inhibitor (DPP-4i), which clearly lack glycosuric adverse effects and are generally considered safe (1) in the overall population and (2) across subgroups of patients with controlled, above-target, or elevated HbA_1c_ levels at baseline.

## Methods

### Study Design and Data Source

We performed a new-user comparative effectiveness and safety research study using a US health insurance data set (deidentified Optum Clinformatics Data Mart Database) with nationwide commercial coverage including Medicare Advantage plans. Through linkage with national laboratory test provider chains, results for outpatient laboratory tests are available for a subset of approximately 45% of beneficiaries (including laboratory test results of HbA_1c_), representative of the full insured population (see eMethods and eTable 1 in Supplement 1). The Mass General Brigham institutional review board provided ethics approval. Informed consent was waived because the study used deidentified secondary data.

### Study Population

The study population included patients 18 years and older who initiated treatment with a SGLT2i (canagliflozin, dapagliflozin, empagliflozin, or ertugliflozin) or a DPP-4i (alogliptin, saxagliptin, linagliptin, or sitagliptin) between April 1, 2013 (consistent with the US Food and Drug Administration [FDA] approval of the first SGLT2i), and June 30, 2021. Treatment with DPP-4i was selected as the comparator because these medications are also frequently used as second-line therapy for T2D, have similar out-of-pocket costs as SGLT2i but a different mechanism of action, which does not involve inhibition of kidney glucose reabsorption and osmotic diuresis, and have shown no association with atherosclerotic cardiovascular outcomes. Cohort entry was the day of the first filled prescription of either SGLT2i or DPP-4i, with no use in the previous 6 months. Study eligibility was limited to patients with at least 6 months of continuous health plan enrollment, a recorded T2D diagnosis before cohort entry, and at least 1 HbA_1c_ laboratory result recorded within 3 months before cohort entry. We excluded patients with records of type 1, secondary, or gestational diabetes; malignant neoplasms; end-stage kidney disease; kidney replacement therapy; no laboratory results for creatinine; or nursing home residence within 6 months preceding cohort entry (eFigure 1 and eTable 2 in Supplement 1). Based on the most recent HbA_1c_ baseline value, we identified 3 different subcohorts which comprised patients with controlled (HbA_1c_ <7.5%), above-target (HbA_1c_ 7.5%-9%), or elevated (HbA_1c_ >9%) glycemia, respectively (to convert percentage of total hemoglobin to proportion of total hemoglobin, multiply by 0.01). The cutoffs for HbA_1c_ stratification were chosen by both inspecting terciles of the HbA_1c_ distribution among SGLT2i treatment initiators and considering the thresholds currently recommended to define controlled vs uncontrolled hyperglycemia.^12,13^

### Outcomes and Follow-up

The primary effectiveness outcomes were (1) modified major adverse cardiovascular events (MACE), a composite cardiovascular end point of myocardial infarction, ischemic or hemorrhagic stroke, and all-cause death, and (2) hospitalization for heart failure (HHF). Secondary effectiveness outcomes were myocardial infarction, ischemic or hemorrhagic stroke, and all-cause mortality. In prior studies, the positive predictive values of claims-based algorithms were at least 87% for myocardial infarction and stroke,^14,15,16^ and 84% to 100% for HHF.^17^ Safety outcomes included hypovolemia, nonvertebral fractures, falls, genital infections, DKA, acute kidney injury (AKI), and lower-limb amputations. Definitions were either validated against medical records^18,19,20,21^ or used in prior pharmacoepidemiologic studies assessing SGLT2i^22,23,24^ (eTable 3 in Supplement 1).

Adopting an as-treated approach, the follow-up started the day after cohort entry and continued until treatment discontinuation (allowing a 30-day grace period after termination of the last prescription’s supply), switch to or augmentation with a drug in the comparator class, occurrence of study outcome, death, end of continuous health plan enrollment, or end of available data, whichever came first.

### Baseline Patient Characteristics

Patient characteristics were selected a priori as potential confounders and measured at treatment initiation (demographics), as last recorded value within 3 months before cohort entry (HbA_1c_), or within 6 months before cohort entry (all other patient characteristics). Covariates included demographics, cardiovascular and other comorbidities, general health state indexes, such as combined comorbidity score and claims-based frailty index,^25,26^ HbA_1c_ laboratory results, diabetes-specific complications, use of glucose-lowering and other medications, indicators of health care utilization as proxy for disease state, surveillance, and intensity of care. Based on baseline creatinine laboratory results, we calculated the estimated glomerular filtration rate (eGFR_Cr_) using a version of the creatinine-based Chronic Kidney Disease–Epidemiology Collaboration (CKD-EPI) equation without the race term as correction factor.^27,28^ Other laboratory test results were also measured at baseline but were available for only a subset of the study population (see eTables 4-6 in Supplement 1 for a complete list of the baseline covariates).

### Statistical Analysis

Propensity score (PS) matching was used to control for confounding. The PS for initiating SGLT2i vs DPP-4i therapy was calculated within each HbA_1c_ subcohort separately through a logistic regression model with 128 prespecified covariates. Laboratory data, except for HbA_1c_ and eGFR_Cr_, were not included in the model because of the substantial proportion of missing information. Initiators of SGLT2i therapy were 1:1 matched to initiators of DPP-4i therapy on their estimated PS within each HbA_1c_ subcohort using the nearest neighbor approach with a caliper width of 0.01 on the PS scale. Covariate balance was assessed with standardized differences, with meaningful imbalances set at values higher than 10%.^29,30^ We also reviewed the balance in laboratory test results not included in the PS model, to evaluate potential residual confounding after PS matching.

We tabulated numbers of events, incidence rates (IRs), and rate differences (RDs) per 1000 person-years. Hazard ratios (HRs) and 95% CIs were estimated by Cox proportional hazard models. We used Kaplan-Meier methods to plot cumulative incidence of primary outcomes and log-rank tests to compare hazard rates between drug classes. Two-sided *P* values for homogeneity were obtained by performing Wald tests and values <.05 were considered indicative of treatment heterogeneity.

We inspected the robustness of the main findings through sensitivity analyses (see eMethods in Supplement 1), addressing potential informative censoring, time-lag bias,^31^ unmeasured confounding for high risk for recurrence, and DPP-4i effects on HHF (since saxagliptin and alogliptin showed an increased HHF rate in CVOTs,^32,33^ which resulted in an FDA warning,^34^ we conducted a sensitivity analysis for the HHF outcome redefining the comparator group as sitagliptin only).

All analyses were implemented using Aetion Evidence Platform (Aetion Inc) and Stata statistical software, version 15.1 (StataCorp LLC).

## Results

### Study Population and Baseline Characteristics

A total of 144 614 eligible adults (mean [SD] age, 62 [12.4] years; 54% male participants) with T2D initiating treatment with a SGLT2i (n = 60 523) or a DPP-4i (n = 84 091) were identified; 44 099 had an HbA_1c_ baseline value of less than 7.5%, 52 986 between 7.5% and 9%, and 47 529 greater than 9%. Overall, patients newly prescribed SGLT2i vs DPP-4i were younger, more likely to have obesity, a higher eGFR_Cr_, and to be treated with more than 1 glucose-lowering medication (particularly glucagon-like peptide-1 receptor agonists and insulin), and less likely to have a diagnosis of diabetic nephropathy or CKD (eTables 4-6 in Supplement 1).

**Figure 1.**
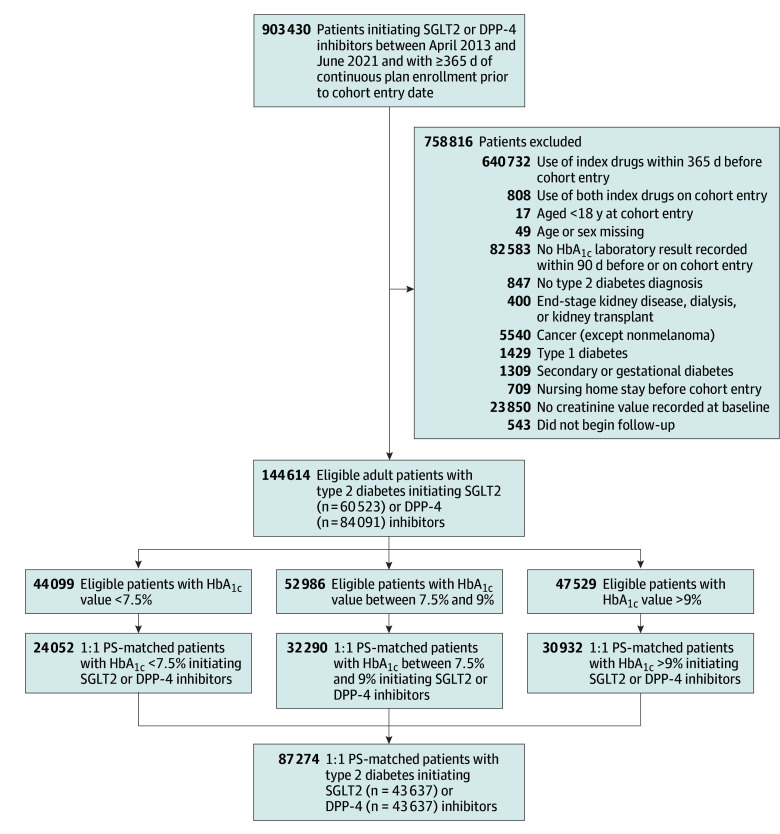
Study Flowchart Eligible population included in the study cohort initiating SGLT2 or DPP-4 inhibitors between April 2013 and June 2021 before and after matching, overall and stratified by HbA_1c_ baseline level. DPP-4 indicates dipeptidyl peptidase-4; HbA_1c_, hemoglobin A_1c_; PS, propensity score; SGLT2, sodium-glucose cotransporter 2.

After PS matching, 87 274 patients were retained: 24 052 with glycemia at target (HbA_1c_ <7.5% mean, 6.8%), 32 290 with glycemia above target (HbA_1c_ 7.5%-9% mean, 8.2%), and 30 932 with elevated glycemia (HbA_1c_ >9% mean, 10.6%) (Figure 1); all baseline characteristics were well balanced (Table), including the laboratory test results not included in the PS model (eTables 4-6 in Supplement 1), and the PS distributions overlapped completely (eFigure 2 in Supplement 1).

**Table.** Selected Baseline Characteristics of Unmatched and 1:1 Propensity Score–Matched Patients Initiating SGLT2 Inhibitor vs DPP-4 Inhibitor Therapy Overall and Stratified by HbA_1c_ Levels

**Characteristics before PS-matching**	**Subgroup HbA_1c_ <7.5%**			**Subgroup HbA_1c_ 7.5%-9%**			**Subgroup HbA_1c_ >9%**			**Overall population**		
	**SGLT2 inhibitor (n = 16 316)**	**DPP-4 inhibitor (n = 27 783)**	**St. Diff**	**SGLT2 inhibitor (n = 22 312)**	**DPP-4 inhibitor (n = 30 674)**	**St. Diff**	**SGLT2 inhibitor (n = 21 895)**	**DPP-4 inhibitor (n = 25 634)**	**St. Diff**	**SGLT2 inhibitor (n = 60 523)**	**DPP-4 inhibitor (n = 84 091)**	**St. Diff**
Age, mean (SD)	61.1 (11.5)	66.7 (11.6)	−0.5	60.8 (11.4)	64.9 (11.9)	−0.4	56.7 (11.9)	60.0 (13.0)	−0.3	59.4 (11.6)	64.0 (12.1)	−0.4
Male, No. (%)	8466 (51.9)	12 942 (46.6)	0.1	12 554 (56.3)	15 567 (50.7)	0.1	12 782 (58.4)	14 047 (54.8)	0.1	33 802 (55.8)	42 556 (50.6)	0.1
Female, No. (%)	7850 (48.1)	14 841 (53.4)	−0.1	9758 (43.7)	15 107 (49.3)	−0.1	9113 (41.6)	11 587 (45.2)	−0.1	26 721 (44.2)	41 535 (49.4)	−0.1
Race, No. (%)												
Asian	1098 (6.7)	2679 (9.6)	−0.1	1329 (6.0)	2548 (8.3)	−0.1	991 (4.5)	1545 (6.0)	−0.1	3418 (5.6)	6772 (8.1)	−0.1
Black	1902 (11.7)	3762(13.5)	−0.1	2495 (11.2)	3851 (12.6)	−0.04	2946 (13.5)	3859 (15.1)	−0.1	7343 (12.1)	11 472 (13.6)	−0.04
Hispanic	3252 (19.9)	6244 (22.5)	−0.1	4970 (22.3)	7838 (25.6)	−0.1	5932 (27.1)	7749 (30.2)	−0.1	14 154 (23.4)	21 831 (26.0)	−0.1
White	9144 (56.0)	13 584 (48.9)	0.1	12 186 (54.6)	14 686 (47.9)	0.1	10 712 (48.9)	11 060 (43.1)	0.1	32 042 (52.9)	39 330 (46.8)	0.1
Other^a^ or unknown	20 (5.6)	1514 (5.4)	0.01	1332 (6.0)	1751 (5.7)	0.01	1314 (6.0)	1421 (5.5)	0.02	3566 (5.9)	4686 (5.6)	0.01
Obesity, No. (%)	6254 (38.3)	6889 (24.8)	0.3	8107 (36.3)	8178 (26.7)	0.2	7952 (36.3)	6932 (27.0)	0.2	22 313 (36.9)	21 999 (26.2)	0.2
Laboratory results, mean (SD)												
HbA_1c_ value, %^b^	6.8 (0.6)	6.8 (0.6)	0.1	8.2 (0.6)	8.2 (0.6)	0.1	10.6 (1.4)	10.6 (1.5)	−0.01	8.7 (1.0)	8.5 (1.0)	0.3
mmol/mol	51 (NA)	50 (NA)	NA	66 (NA)	66 (NA)	NA	91 (NA)	93 (NA)	NA	72 (NA)	69 (NA)	NA
eGFR_Cr_^c^	76.2 (23.9)	68.0 (24.4)	0.3	78.7 (23.4)	73.4 (24.2)	0.2	84.0 (24.5)	80.1 (25.3)	0.2	80.0 (23.9)	73.7 (24.6)	0.3
Burden of comorbidities, mean (SD)												
Combined comorbidity score^d^	1.1 (2.0)	1.4 (2.3)	−0.2	1.0 (1.8)	1.2 (2.0)	−0.1	1.0 (1.7)	1.0 (1.9)	−0.03	1.0 (1.8)	1.2 (2.1)	−0.1
Frailty score^e^	0.2 (0.04)	0.2 (0.1)	<0.01	0.1 (0.04)	0.2 (0.04)	−0.3	0.1 (0.04)	0.1 (0.04)	<0.01	0.1 (0.04)	0.2 (0.04)	−0.3
Diabetes-related comorbidities, No. (%)												
Diabetic nephropathy	2353 (14.4)	5948 (21.4)	−0.2	3350 (15.0)	5884 (19.2)	−0.11	2830 (12.9)	4019 (15.7)	−0.1	8533 (14.1)	15 851 (18.8)	−0.13
Diabetic retinopathy	964 (5.9)	1803 (6.5)	−0.02	1842 (8.3)	2473 (8.1)	0.01	1718 (7.8)	1986 (7.7)	<0.01	4524 (7.5)	6262 (7.4)	<0.01
Lower-limb amputations	66 (0.4)	120 (0.4)	<0.01	104 (0.5)	146 (0.5)	<0.01	145 (0.7)	169 (0.7)	<0.01	315 (0.5)	435 (0.5)	<0.01
Diabetic ketoacidosis	22 (0.1)	35 (0.1)	<0.01	32 (0.1)	42 (0.1)	<0.01	66 (0.3)	78 (0.3)	<0.01	120 (0.2)	155 (0.2)	<0.01
Other comorbidities, No. (%)												
History of cardiovascular disease	3873 (23.7)	7194 (25.9)	−0.1	4783 (21.4)	7110 (23.2)	−0.04	3953 (18.1)	4636 (18.1)	<0.01	12 609 (20.8)	18 940 (22.5)	−0.04
Heart failure	1284 (7.9)	2378 (8.6)	−0.03	1325 (5.9)	2093 (6.8)	−0.04	1402 (5.5)	1633 (5.6)	<0.01	4011 (6.6)	6104 (7.3)	−0.03
Chronic kidney disease, stage <3	1156 (7.1)	4930 (17.7)	−0.3	1333 (6.0)	3868 (12.6)	−0.2	941 (4.3)	2243 (8.8)	−0.2	3430 (5.7)	11 041 (13.1)	−0.3
Acute kidney injury	368 (2.3)	1235 (4.4)	−0.1	368 (1.6)	841 (2.7)	−0.1	328 (1.5)	596 (2.3)	−0.1	1064 (1.8)	2672 (3.2)	−0.1
Mycotic infections	1389 (8.5)	2638 (9.5)	−0.03	1857 (8.3)	2912 (9.5)	−0.04	1986 (9.1)	2440 (9.5)	−0.01	5232 (8.6)	7990 (9.5)	−0.03
Fractures	138 (0.8)	288 (1.0)	−0.02	144 (0.6)	258 (0.8)	−0.02	144 (0.7)	191 (0.7)	<0.01	426 (0.7)	737 (0.9)	−0.02
Falls	353 (2.2)	802 (2.9)	−0.04	441 (2.0)	736 (2.4)	−0.03	437 (2.0)	540 (2.1)	−0.01	1231 (2.0)	2078 (2.5)	−0.03
Diabetes treatment												
No use of any glucose-lowering drugs at baseline; No. (%)	2610 (16.0)	6053 (21.8)	−0.2	1898 (8.5)	3843 (12.5)	−0.1	2584 (11.8)	4630 (18.1)	−0.2	7092 (11.7)	14 526 (17.3)	−0.2
No. glucose-lowering drugs, mean (SD)^f^	1.1 (0.9)	1.0 (0.8)	0.2	1.4 (0.9)	1.2 (0.8)	0.2	1.4 (0.9)	1.3 (0.8)	0.1	1.3 (0.9)	1.2 (0.8)	<0.01
Metformin, No. (%)^f^	10 120 (62.0)	16 545 (59.6)	0.1	14 915 (66.8)	20 337 (66.3)	0.01	14 364 (65.6)	17 562 (68.5)	−0.1	39 399 (65.1)	54 444 (64.7)	0.01
Sulfonylureas (second generation), No. (%)^f^	2863 (17.5)	6334 (22.8)	−0.1	6756 (30.3)	11 174 (36.4)	−0.1	6263 (28.6)	9159 (35.7)	−0.2	15 882 (26.2)	26 667 (31.7)	−0.1
GLP-1 receptor agonists, No. (%)^f^	2464 (15.1)	580 (2.1)	0.5	3637 (16.3)	802 (2.6)	0.5	3510 (16.0)	806 (3.1)	0.5	9611 (15.9)	2188 (2.6)	0.5
Insulin, No. (%)^f^	1750 (10.7)	1536 (5.5)	0.2	4226 (18.9)	3405 (11.1)	0.2	5433 (24.8)	4476 (17.5)	0.2	11 409 (18.9)	9417 (11.2)	0.2
**Characteristics after PS-matching**	**Subgroup HbA_1c_ <7.5%**			**Subgroup HbA_1c_ 7.5%-9%**			**Subgroup HbA_1c_ >9%**			**Overall Population**		
	**SGLT2 inhibitor (n = 12 026)**	**DPP-4 inhibitor (n = 12 026)**	**St. Diff**	**SGLT2 inhibitor (n = 16 145)**	**DPP-4 inhibitor (n = 16 145)**	**St. Diff**	**SGLT2 inhibitor (n = 15 466)**	**DPP-4 inhibitor (n = 15 466)**	**St. Diff**	**SGLT2 inhibitor (n = 43 637)**	**DPP-4 inhibitor (n = 43 637)**	**St. Diff**
Age, mean (SD)	62.5 (11.4)	62.3 (11.3)	0.01	62.0 (11.5)	62.0 (11.4)	0.01	57.6 (12.2)	57.7 (12.1)	−0.01	60.6 (11.7)	60.6 (11.6)	<0.01
Male, No. (%)	6034 (50.2)	6047 (50.3)	<0.01	8794 (54.5)	8784 (54.4)	<0.01	8846 (57.2)	8859 (57.3)	<0.01	23 674 (54.3)	23 690 (54.3)	<0.01
Female, No. (%)	5992 (49.8)	5979 (49.7)	<0.01	7351 (45.5)	7361 (45.6)	<0.01	6620 (42.8)	6607 (42.7)	<0.01	19 963 (45.7)	19 947 (45.7)	<0.01
Race, No. (%)												
Asian	914 (7.6)	906 (7.5)	<0.01	1092 (6.8)	1113 (6.9)	<0.01	795 (5.1)	785 (5.1)	<0.01	2801 (6.4)	2804 (6.4)	<0.01
Black	1483 (12.3)	1471 (12.2)	<0.01	1930 (12.0)	1890 (11.7)	0.01	2193 (14.2)	2184 (14.1)	<0.01	5606 (12.8)	5545 (12.7)	<0.01
Hispanic	2482 (20.6)	2513 (20.9)	−0.01	3807 (23.6)	3768 (23.3)	0.01	4418 (28.6)	4439 (28.7)	<0.01	10 707 (24.5)	10 720 (24.6)	<0.01
White	6463 (53.7)	6436 (53.5)	<0.01	8358 (51.8)	8380 (51.9)	<0.01	7138 (46.2)	7141 (46.2)	<0.01	21 959 (50.3)	21 957 (50.3)	<0.01
Other^a^ or unknown	684 (5.7)	700 (5.8)	<0.01	958 (5.9)	994 (6.2)	−0.01	922 (6.0)	917 (5.9)	<0.01	2564 (5.9)	2611 (6.0)	<0.01
Obesity, No. (%)	3995 (33.2)	3959 (32.9)	0.01	5198 (32.2)	5209 (32.3)	<0.01	4941 (31.9)	4922 (31.8)	<0.01	14 134 (32.4)	14 090 (32.3)	<0.01
Laboratory results, mean (SD)												
HbA_1c_ value %^b^	6.8 (0.6)	6.8 (0.6)	0.02	8.2 (0.6)	8.2 (0.6)	<0.01	10.6 (1.4)	10.6 (1.4)	<0.01	8.7 (1.0)	8.7 (1.0)	<0.01
mmol/mol	51 (NA)	51 (NA)	NA	66 (NA)	66 (NA)	NA	93 (NA)	93 (NA)	NA	71 (NA)	71 (NA)	NA
eGFR_Cr_^c^	74.9 (23.6)	75.1 (23.6)	−0.01	77.4 (23.3)	77.8 (23.5)	−0.02	83.2 (24.4)	83.2 (24.5)	<0.01	78.8 (23.8)	79.0 (23.9)	−0.01
Burden of comorbidities, mean (SD)												
Combined comorbidity score^d^	1.1 (2.0)	1.1 (2.0)	<0.01	1.0 (1.9)	1.0 (1.8)	0.01	0.9 (1.7)	0.9 (1.7)	0.01	1.0 (1.9)	1.0 (1.8)	0.01
Frailty index^e^	0.2 (0.04)	0.2 (0.04)	<0.01	0.1 (0.04)	0.1 (0.04)	<0.01	0.1 (0.04)	0.1 (0.04)	<0.01	0.14 (0.04)	0.14 (0.04)	<0.01
Diabetes-related comorbidities, No. (%)												
Diabetic nephropathy	1818 (15.1)	1788 (14.9)	0.01	2477 (15.3)	2460 (15.2)	<0.01	2042 (13.2)	2051 (13.3)	<0.01	6337 (14.5)	6299 (14.4)	<0.01
Diabetic retinopathy	672 (5.6)	677 (5.6)	<0.01	1267 (7.8)	1261 (7.8)	<0.01	1134 (7.3)	1139 (7.4)	<0.01	3073 (7.0)	3077 (7.1)	<0.01
Lower-limb amputations	44 (0.4)	40 (0.3)	0.02	73 (0.5)	76 (0.5)	<0.01	90 (0.6)	94 (0.6)	<0.01	207 (0.5)	210 (0.5)	<0.01
Diabetic ketoacidosis	14 (0.1)	17 (0.1)	<0.01	21 (0.1)	19 (0.1)	<0.01	43 (0.3)	49 (0.3)	<0.01	78 (0.2)	85 (0.2)	<0.01
Other comorbidities, No. (%)												
History of cardiovascular disease	2806 (23.3)	2739 (22.8)	0.01	3462 (21.4)	3374 (20.9)	0.01	2625 (17.0)	2663 (17.2)	−0.01	8893 (20.4)	8776 (20.1)	0.01
Heart failure	874 (7.3)	861 (7.2)	<0.01	951 (5.9)	929 (5.8)	<0.01	767 (5.0)	731 (4.7)	0.01	2592 (5.9)	2521 (5.8)	<0.01
Chronic kidney disease, stage <3	1009 (8.4)	974 (8.1)	0.01	1158 (7.2)	1121 (6.9)	0.01	817 (5.3)	777 (5.0)	0.01	2984 (6.8)	2872 (6.6)	0.01
Acute kidney injury	286 (2.4)	302 (2.5)	−0.01	289 (1.8)	288 (1.8)	<0.01	254 (1.6)	250 (1.6)	<0.01	829 (1.9)	840 (1.9)	<0.01
Mycotic infections	1013 (8.4)	975 (8.1)	0.01	1365 (8.5)	1383 (8.6)	<0.01	1418 (9.2)	1421 (9.2)	<0.01	3796 (8.7)	3779 (8.7)	<0.01
Fractures	105 (0.9)	99 (0.8)	0.01	111 (0.7)	103 (0.6)	0.01	108 (0.7)	102 (0.7)	<0.01	324 (0.7)	304 (0.7)	<0.01
Falls	272 (2.3)	260 (2.2)	0.01	341 (2.1)	353 (2.2)	−0.01	306 (2.0)	296 (1.9)	0.01	919 (2.1)	909 (2.1)	<0.01
Diabetes treatment												
No use of any glucose-lowering drugs at baseline; No. (%)	2266 (18.8)	2276 (18.9)	<0.01	1699 (10.5)	1687 (10.4)	<0.01	2308 (14.9)	2290 (14.8)	<0.01	6273 (14.4)	6253 (14.3)	<0.01
No. glucose-lowering drugs, mean (SD)^f^	1.0 (0.8)	1.0 (0.8)	<0.01	1.3 (0.9)	1.3 (0.8)	0.01	1.3 (0.9)	1.3 (0.8)	−0.01	1.2 (0.9)	1.2 (0.8)	<0.01
Metformin, No. (%)^f^	7400 (61.5)	7573 (63.0)	−0.03	10 788 (66.8)	11 062 (68.5)	−0.04	10 360 (67.0)	10 584 (68.4)	−0.03	28 548 (65.4)	29 219 (67.0)	−0.03
Sulfonylureas (second generation), No. (%)^f^	2280 (19.0)	2311 (19.2)	−0.01	5269 (32.6)	5300 (32.8)	<0.01	4937 (31.9)	4937 (31.9)	<0.01	12 486 (28.6)	12 548 (28.8)	<0.01
GLP-1 receptor agonists, No. (%)^f^	754 (6.3)	537 (4.5)	0.1	1024 (6.3)	760 (4.7)	0.1	976 (6.3)	772 (5.0)	0.1	2754 (6.3)	2069 (4.7)	0.1
Insulin, No. (%)^f^	939 (7.8)	838 (7.0)	0.03	2268 (14.0)	2199 (13.6)	0.01	3109 (20.1)	3086 (20.0)	<0.01	6316 (14.5)	6123 (14.0)	0.01

Abbreviations: DPP-4i, dipeptidyl peptidase 4 inhibitors; eGFR_Cr_, creatinine-based estimated glomerular filtration rate; GLP-1, glucagon-like peptide-1; HbA_1c_, hemoglobin A_1c_; NA, not applicable; PS, propensity score; SGLT2, sodium-glucose cotransporter-2; St. Diff, standardized differences, ie, the difference in means or proportions divided by the pooled.

^a^ American Indian, Alaska Native, Native Hawaiian, or other Pacific Islander.

^b^ To convert to proportion of total hemoglobin, multiply by 0.01.

^c^ eGFR_Cr_ has been estimated applying the creatinine-based Chronic Kidney Disease–Epidemiology Collaboration (CKD-EPI) equation with no inclusion of race as correction factor.

^d^ A higher combined comorbidity score is associated with a greater risk of mortality during the follow-up.

^e^ Individuals are considered prefrail when the frailty index is between 0.15 and 0.24 and frail when the frailty index is at least 0.25.

^f^ Treatment prescriptions overlapping the date of initiation of the study drugs (ie, concurrent use).

In the overall population, 6.7% had moderate to advanced CKD, 8.7% had a history of mycotic infection, 0.7% had a history of fractures, 2.1% had a history of falls, and 14.0% were prescribed insulin on the day of cohort entry. Patients with HbA_1c_ levels of more than 9% treated with SGLT2i were younger than those with HbA_1c_ levels between 7.5% and 9% and those with HbA_1c_ levels less than 7.5% (57.7 vs 62.0 vs 62.5 years, respectively), mostly male participants (57.2% vs 54.5% vs 50.2%), less frequently White (46.2% vs 51.8% vs 53.7%), and more likely to receive insulin (20.1% vs 14.0% vs 7.8%). They had higher eGFR_Cr_ (83.2 vs 77.4 vs 74.9) and lower burden of comorbidities and frailty. Compared with SGLT2i treatment initiators with HbA_1c_ levels of less than 7.5% and greater than 9%, those with HbA_1c_ levels between 7.5% and 9% had higher prevalence of diabetes-related complications such as diabetic nephropathy (15.3% vs 15.1% vs 13.2%) and retinopathy (7.8% vs 5.6% vs 7.3%) and lower prevalence of untreated diabetes at baseline (Table).

Duration of follow-up on treatment varied slightly based on the outcome. In the overall population, the mean follow-up was 240 days for modified MACE and 241 days for HHF. Most patients were censored due to treatment discontinuation (approximately 60%). Details on follow-up and censoring reasons are reported in eTable 7 in Supplement 1.

### Primary Effectiveness Outcomes Analyses

**Figure 2.**
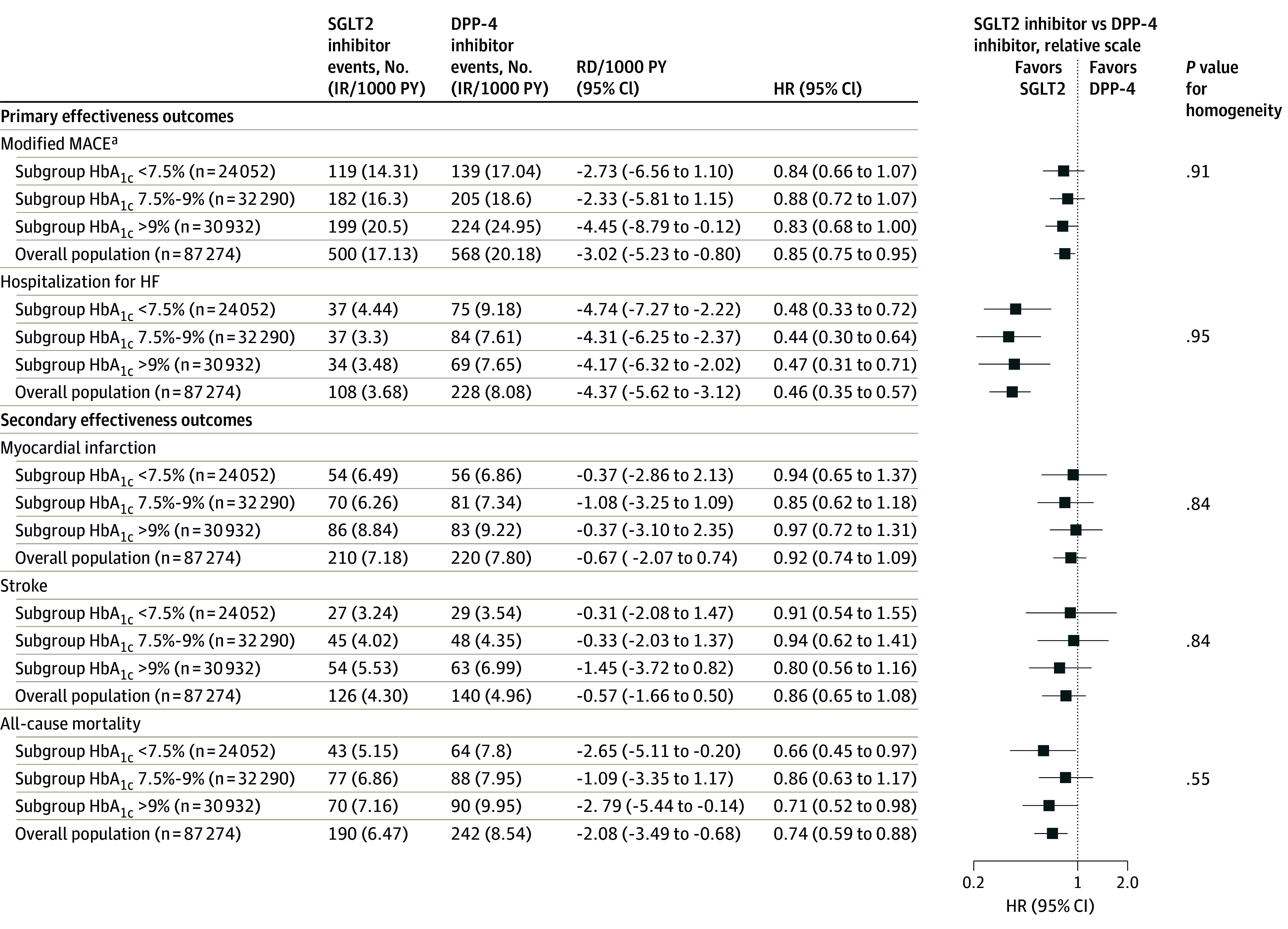
Primary and Secondary Effectiveness Outcomes in 1:1 Propensity Score–Matched Patients Initiating SGLT2 Inhibitor vs DPP-4 Inhibitor Therapy Stratified by HbA_1c_ Levels Number of events and incidence rates (IR) by treatment group and the point estimates of the effect sizes are shown overall and for HbA_1c_ subcohorts. Hazard ratios are indicated by squares; 95% CIs, by horizontal lines. DPP-4 indicates dipeptidyl peptidase 4; HbA_1c_, hemoglobin A_1c_; HR, hazard ratio; MACE, major adverse cardiovascular events; PY, person-years; RD, rate difference; SGLT2, sodium-glucose cotransporter 2. ^a^Modified MACE is composite outcome including myocardial infarction and stroke events and all-cause deaths. A detailed definition is reported in Supplement 1.

After PS matching, the IRs per 1000 person-years for modified MACE were overall 17.13 vs 20.18 in SGLT2i vs DPP-4i initiators, respectively, showing among new users of SGLT2i vs DPP-4i a 15% decreased risk (HR, 0.85; 95% CI, 0.75-0.95), or 3 fewer events in 1000 person-years (RD –3.02; 95% CI, –5.23 to −0.80). The results across subgroups were consistent with the overall findings with no evidence of effect heterogeneity (HbA_1c_ <7.5% HR, 0.84; 95% CI, 0.66-1.07; HbA_1c_ 7.5%-9% HR, 0.88; 95% CI, 0.72-1.07; and HbA_1c_ >9% HR, 0.83; 95% CI, 0.68-1.00; *P* for homogeneity = .91), although the degree of uncertainty was higher and the point estimates less precise due to the reduced statistical power (Figure 2).

Overall, 3.68 vs 8.08 HHF events per 1000 person-years were estimated in SGLT2i vs DPP-4i treatment initiators, respectively. The initiation of SGLT2i vs DPP-4i was associated with a 54% decreased risk of HHF (HR, 0.46; 95% CI, 0.35-0.57), corresponding to approximately 4 fewer cases per 1000 person-years (RD −4.37; 95% CI, −5.62 to −3.12). This was consistent across subgroups with no evidence of effect heterogeneity (HbA_1c_ <7.5% HR, 0.48; 95% CI, 0.33-0.72; HbA_1c_ 7.5%-9% HR, 0.44; 95% CI, 0.30-0.64; HbA_1c_ >9% HR, 0.47; 95% CI, 0.31-0.71; *P* = .95 for homogeneity) (Figure 2).

**Figure 3.**
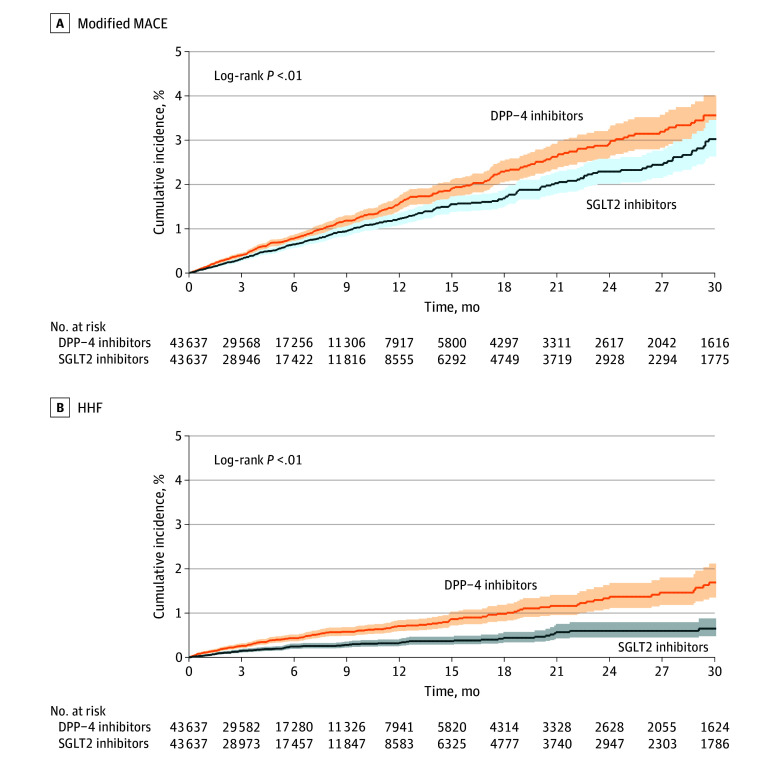
Cumulative Incidence of Modified MACE and HHF Comparing 1:1 Propensity Score–Matched Patients Initiating SGLT2 Inhibitor vs DPP-4 Inhibitor Therapy DPP-4 indicates dipeptidyl peptidase 4; HHF, hospitalization for heart failure; MACE, major adverse cardiovascular events; SGLT2, sodium-glucose cotransporter 2.

Kaplan-Meier curves comparing the cumulative incidence of modified MACE and HHF between initiators of SGLT2 vs DPP-4i therapy were consistent with these results and across subgroups (Figure 3 and eFigure 3 in Supplement 1). Clinical benefits were observed within the first 3 months of follow-up (Figure 3).

### Secondary Effectiveness Outcomes Analyses

No overall differences between SGLT2i and DPP-4i treatments in the risk of myocardial infarction (HR, 0.92; 95% CI, 0.74-1.09) or stroke (HR, 0.86; 95% CI, 0.65-1.08) were found. In the overall population, the initiation of treatment with SGLT2i vs DPP-4i was associated with a 26% reduced risk of all-cause mortality (HR, 0.74; 95% CI, 0.59-0.88), corresponding to 2 fewer deaths per 1000 person-years. No evidence of effect heterogeneity on either the relative or the absolute scale was found between subgroups for any of the secondary outcomes (Figure 2).

### Safety Outcomes Analyses

**Figure 4.**
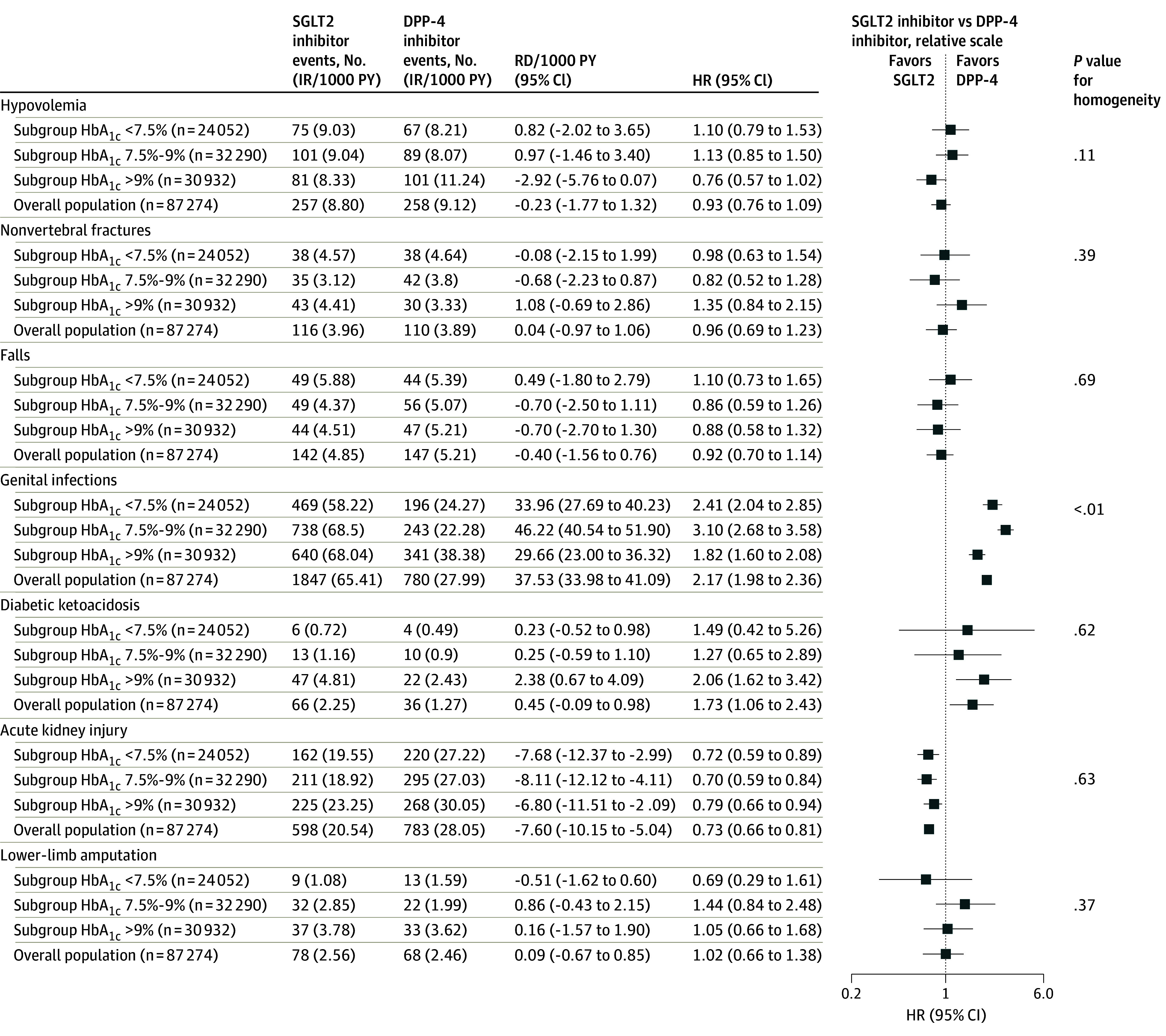
Safety Outcomes in 1:1 Propensity Score–Matched Patients Initiating SGLT2 Inhibitor vs DPP-4 Inhibitor Therapy Stratified by HbA_1c_ Levels Number of events and incidence rates (IR) by treatment group and point estimates of the effect sizes are shown overall and for HbA_1c_ subcohorts. Hazard ratios are indicated by squares; 95% CIs, by horizontal lines. DPP-4 indicates dipeptidyl peptidase 4; HbA_1c_, hemoglobin A_1c_; HR, hazard ratio; PY, person-years; RD, rate difference; SGLT2, sodium-glucose cotransporter 2.

The risks of hypovolemia, nonvertebral fractures, falls and lower-limb amputations were similar among patients initiating treatment with SGLT2i vs DPP-4i (Figure 4). In the overall population, SGLT2i vs DPP-4i initiators had a 2.17-fold increased risk of genital infections (HR, 2.17; 95% CI, 1.98-2.36), corresponding to approximately 38 additional events per 1000 person-years, with evidence of treatment effect heterogeneity across subgroups on both the relative and absolute scales (*P* < .01 for homogeneity). Patients with HbA_1c_ levels of 7.5% to 9% had a 3.1-fold increased risk for yeast infections (IR per 1000 person-years 68.5 vs 22.8, respectively; HR, 3.10; 95% CI, 2.68-3.58) vs an approximately 2-fold increased risk in patients with HbA_1c_ levels of less than 7.5% (HR, 2.41; 95% CI, 2.04-2.85) and greater than 9% (HR, 1.82; 95% CI, 1.60-2.08), corresponding to RD of 46.22 (95% CI, 40.54-51.90) for HbA_1c_ 7.5%-9%, vs 33.96 (95% CI, 27.69-40.23) for HbA_1c_ <7.5%, and 29.66 (95% CI, 23.00-36.32) for HbA_1c_ >9% additional cases per 1000 person-years. Overall, a 1.7-fold increased risk of DKA was found in SGLT2i treatment initiators (HR, 1.73; 95% CI, 1.06-2.43). Although the estimates are less precise and the uncertainty is higher due to the low number of DKA events within each HbA_1c_ subgroup, the stratified results appear consistent with the overall finding (Figure 4). In the overall cohort, a 27% decreased risk of AKI was associated with the initiation of SGLT2i vs DPP-4i (HR, 0.73; 95% CI, 0.66-0.81), corresponding to approximately 8 fewer cases per 1000 patient-years. Similar results were obtained in subgroup analyses by HbA_1c_ with no evidence of treatment effect heterogeneity by HbA_1c_ (Figure 4).

### Sensitivity Analyses

Findings remained consistent when an intention-to-treat approach was adopted and when the internal validity of the effectiveness and safety outcome analyses was tested (eTables 8-9 in Supplement 1) with some fluctuations in point estimates driven by the small number of events in the subgroup analyses by HbA_1c_. No difference in treatment effect was found in patients with vs without cardiovascular diseases.

## Discussion

In this large comparative effectiveness and safety research study of 87 274 adults with T2D, including 24 052 with controlled HbA_1c_ levels, 32 290 with above-target HbA_1c_ levels, and 30 932 with elevated baseline HbA_1c_ levels, we found that (1) initiating treatment with SGLT2i was associated with a reduced risk of modified MACE, HHF, and AKI and a higher risk of genital infections and DKA compared with DPP-4i; and that (2) the results did not vary based on preexposure HbA_1c_ levels for most outcomes evaluated. Although individual characteristics differed among subgroups (for example patients with elevated HbA_1c_ were younger, more likely to receive insulin, had a higher eGFR_Cr_ and fewer comorbidities than others), benefits and adverse effects of SGLT2i vs DPP-4i were similar across HbA_1c_ subcohorts.

Overall, patients receiving a SGLT2i had a 15% lower risk of the composite of myocardial infarction, stroke, or death from all causes (approximately 3 fewer cases per 1000 person-years) and a 54% lower risk of HHF (approximately 4 fewer cases per 1000 person-years) than those receiving a DPP-4i. These findings with respect to the modified MACE outcome parallel those of the placebo-controlled CANVAS trial for canagliflozin,^4^ and the placebo-controlled EMPA-REG OUTCOME trial for empagliflozin,^3^ and of large cohort studies comparing SGLT2i vs DPP-4i.^7,8,35,36,37^ Similarly, our HHF results are in line with those from CVOTs,^3,4,5,6^ and large comparative effectiveness studies.^37,38^ The effect estimates were consistent across HbA_1c_ subgroups for both modified MACE (HR range, 0.83-0.88), in line with exploratory analyses from a network meta-analysis^39^ and HHF (HR range, 0.46-0.48).

The safety analyses showed that overall patients initiating a SGLT2i had a higher risk of genital infections and DKA, both of which are known adverse effects of these medications,^21,22,40^ and a lower risk of AKI, a previously observed benefit,^22,41,42^ than those receiving a DPP-4i. Rates of hypovolemia, falls, bone fracture events, and lower-limb amputations were similar in the SGLT2i and DPP-4i groups. The analysis of the safety profile of SGLT2i across patients with different HbA_1c_ levels, which has not been investigated in CVOTs, is a main strength of our study. Medications in the SGLT2i class are responsible for pharmacologically induced renal glycosuria by suppressing sodium and glucose reabsorption in the proximal tubule. Given that the urinary glucose concentration and consequent osmotic diuresis is higher in SGLT2i users with uncontrolled glycemia than in those with better controlled glycemia, a common hypothesis is that the risk of hypovolemia (and consequent falls and fractures) from polyuria, genitourinary infections from glucosuria, amputations from a reduced limb perfusion due to hypotension and an increased risk of peripheral ischemia due to hemoconcentration and hyperviscosity, and DKA from decreased plasma glucose and insulin release might have been further increased in patients with elevated HbA_1c_ compared with patients with lower HbA_1c_.^43,44,45^ The findings of this study do not support this hypothesis. Results were largely consistent across all HbA_1c_ subcohorts, except for some evidence of treatment effect heterogeneity for genital infections, with the highest risk observed in patients with baseline HbA_1c_ levels between 7.5% and 9%. This subgroup had the highest prevalence of both diabetes-related complications and prescriptions of glucose-lowering medications, suggesting a more advanced stage of diabetes compared with other subgroups. This may explain the increased risk of yeast infections observed in these patients.

This study augments the evidence provided by CVOTs of SGLT2i, showing that patients with severe uncontrolled diabetes can benefit from the use of these medications in a fashion similar to patients with better controlled glycemia, with no further increase in the risk of adverse effects. The population of patients identified in this study is 5 to 9 times larger than the populations included in the CVOTs. Because of the larger sample, we were able to identify 3 subgroups of patients with different ranges of HbA_1c_, and thus explore with more granularity and reduced level of uncertainty the influence of increasing glycemic levels on the safety and effectiveness of SGLT2i treatment. Another strength of this study is better generalizability of the findings to routine care. Several studies reported that a considerable number of patients with T2D cared for in clinical practice do not have characteristics similar to the patient populations included in CVOTs.^46,47,48^ A recent review showed that if the enrollment criteria of CANVAS,^4^ EMPA-REG OUTCOME,^3^ and VERTIS-CV^6^ were applied to the real-world population, only 17% to 36% of patients with T2D would have been eligible, with only 49.5% of real-world patients with T2D eligible for a CVOT with broader inclusion criteria such as the DECLARE-TIMI-58 trial.^48^ Further, while CVOTs restrict to patients with cardiorenal diseases or multiple risk factors to achieve adequate statistical power in the time frame of the trials, we examined the comparative effectiveness of these drugs across the broader spectrum of cardiovascular risk. Lastly, adopting an active-comparator new-user design largely reduced the risk of biased findings, increasing the study validity.^49,50^

### Limitations

This study has limitations. First, residual confounding for unmeasured characteristics, such as duration of diabetes or body mass index, cannot be entirely ruled out. However, we observed that covariates not included in the PS model (laboratory test results available only for a subset of the analytic cohort) were balanced after adjustment. Additionally, compared with other large cohort studies that compared SGLT2 vs DPP-4i,^7,8,22,23,24,38,40,41^ we addressed potential confounding by diabetes severity and kidney function by controlling for eGFR_Cr_. Second, the stratification by HbA_1c_ levels reduced precision of some outcome estimates within subgroups, such as for modified MACE and DKA. However, the direction and magnitude of the effect for these outcomes were consistent with the overall findings, supporting the lack of effect modification by HbA_1c_. Third, as this study is based on routine care use of SGLT2i or DPP-4i, the mean follow-up (ie, time on treatment) was shorter compared with CVOTs, which introduce substantial measures to improve treatment adherence. Contrary to randomized clinical trials that require long follow-up to accumulate sufficient events for powered analyses, the size of this study population allowed us to generate overall results with high precision despite a shorter length of follow-up. Additionally, several trials showed that SGLT2i rapidly reduced the risk of cardiovascular death or HHF in patients with T2D, with benefits that are sustained over time.^51,52,53,54^ Thus, assuming no time-varying hazards, these results should be generalizable to longer-term findings. Fourth, potential for outcome misclassification cannot be entirely excluded; however, the validated outcome definitions used in this study have high positive predictive value and specificity and are not expected to differ by treatment group. Last, we could not evaluate cardiovascular death due to the lack of information on cause of death in the data. Death for all causes may be limited by incomplete death records in this data set, though we would not expect this to differ by treatment groups.

## Conclusions

In this large comparative effectiveness and safety study of 87 274 adults with T2D, patients who initiated SGLT2i therapy had a reduced risk of MACE, HHF, and AKI, and an increased risk of genital infections and DKA, compared with DPP-4i. The cardiovascular effectiveness and safety of SGLT2i vs DPP-4i did not vary based on baseline HbA_1c_ levels. This study complements the evidence provided by CVOTs by showing that patients with T2D can benefit from the use of SGLT2i regardless of glycemic control, with no additional increase in the risk of adverse effects in patients with above-target or elevated HbA_1c_ levels, compared with DPP-4i initiators with similar glycemic control.

## Notes

Supplement 1. **eMethods.** Supplementary methods **eTable 1.** Baseline characteristics of eligible patients with vs. without a HbA_1c_ value within 3 months before cohort entry. **eFigure 1.** Study design diagram and definition of the cohort **eTable 2.** Definitions of inclusion and exclusion criteria **eTable 3.** Outcome definitions **eTable 4.** Baseline characteristics of patients with HbA_1c_ < 7.5% initiating SGLT2 inhibitor vs. DPP4 inhibitor therapy before and after 1:1 PS-matching **eTable 5.** Baseline characteristics of patients with HbA_1c_ between 7.5% and 9% initiating SGLT2 inhibitor vs. DPP4 inhibitor therapy before and after 1:1 PS-matching **eTable 6.** Baseline characteristics of patients with HbA_1c_ > 9% initiating SGLT2 inhibitor vs. DPP4 inhibitor therapy before and after 1:1 PS-matching **eFigure 2.** Propensity score distributions of SGLT2i and DPP4i initiators in the overall population. **eTable 7.** Follow-up and censoring reasons after PS-matching (primary outcomes) **eFigure 3.** Cumulative incidence of modified MACE and HHF comparing 1:1 propensity score-matched patients initiating SGLT2 inhibitor vs. DPP-4 inhibitor therapy stratified by HbA_1c_ levels. **eTable 8.** Time-limited “intent-to-treat” analysis and secondary analyses of the primary effectiveness outcomes in 1:1 propensity score-matched patients initiating SGLT2 inhibitor vs. DPP-4 inhibitor therapy and stratified by HbA_1c_ levels **eTable 9.** Number of events, incidence rates, relative and absolute estimates for sensitivity analysis of incident safety outcomes in 1:1 PS-matched initiators of SGLT2 inhibitor vs. DPP4 inhibitor therapy

Supplement 2. Data Sharing Statement
