# Supplementary material for: Comparing Effectiveness and Safety of SGLT2 Inhibitors vs DPP-4 Inhibitors in Patients With Type 2 Diabetes and Varying Baseline HbA1c Levels
Source: JAMA Intern Med. 2023 Feb 6;183(3):242–54. doi: 10.1001/jamainternmed.2022.6664 (PMC9989905; doi:10.1001/jamainternmed.2022.6664)
Supplement: Supplement 1. — eMethods. Supplementary methods eTable 1. Baseline characteristics of eligible patients with vs. without a HbA1c value within 3 months before cohort entry. eFigure 1. Study design diagram and definition of the cohort eTable 2. Definitions of inclusion and exclusion criteria eTable 3. Outcome definitions eTable 4. Baseline characteristics of patients with HbA1c < 7.5% initiating SGLT2 inhibitor vs. DPP4 inhibitor therapy before and after 1:1 PS-matching eTable 5. Baseline characteristics of patients with HbA1c between 7.5% and 9% initiating SGLT2 inhibitor vs. DPP4 inhibitor therapy before and after 1:1 PS-matching eTable 6. Baseline characteristics of patients with HbA1c > 9% initiating SGLT2 inhibitor vs. DPP4 inhibitor therapy before and after 1:1 PS-matching eFigure 2. Propensity score distributions of SGLT2i and DPP4i initiators in the overall population. eTable 7. Follow-up and censoring reasons after PS-matching (primary outcomes) eFigure 3. Cumulative incidence of modified MACE and HHF comparing 1:1 propensity score-matched patients initiating SGLT2 inhibitor vs. DPP-4 inhibitor therapy stratified by HbA1c levels. eTable 8. Time-limited “intent-to-treat” analysis and secondary analyses of the primary effectiveness outcomes in 1:1 propensity score-matched patients initiating SGLT2 inhibitor vs. DPP-4 inhibitor therapy and stratified by HbA1c levels eTable 9. Number of events, incidence rates, relative and absolute estimates for sensitivity analysis of incident safety outcomes in 1:1 PS-matched initiators of SGLT2 inhibitor vs. DPP4 inhibitor therapy [file jamainternmed-e226664-s001.pdf]

Supplemental Online Content

D'Andrea E, Wexler DJ, Kim SC, Paik JM, Alt E, Patorno E. Comparing effectiveness and safety of SGLT2 inhibitors vs DPP-4 inhibitors in patients with type 2 diabetes and varying baseline HbA_{1c} levels. *JAMA Intern Med.* Published online February 6, 2023. doi:10.1001/jamainternmed.2022.6664

eMethods. Supplementary methods

eTable 1. Baseline characteristics of eligible patients with vs. without a HbA_{1c} value within 3 months before cohort entry.

eFigure 1. Study design diagram and definition of the cohort

eTable 2. Definitions of inclusion and exclusion criteria

eTable 3. Outcome definitions

eTable 4. Baseline characteristics of patients with HbA_{1c} < 7.5% initiating SGLT2 inhibitor vs. DPP4 inhibitor therapy before and after 1:1 PS-matching

eTable 5. Baseline characteristics of patients with HbA_{1c} between 7.5% and 9% initiating SGLT2 inhibitor vs. DPP4 inhibitor therapy before and after 1:1 PS-matching

eTable 6. Baseline characteristics of patients with HbA_{1c} > 9% initiating SGLT2 inhibitor vs. DPP4 inhibitor therapy before and after 1:1 PS-matching

eFigure 2. Propensity score distributions of SGLT2i and DPP4i initiators in the overall population.

eTable 7. Follow-up and censoring reasons after PS-matching (primary outcomes)

eFigure 3. Cumulative incidence of modified MACE and HHF comparing 1:1 propensity score-matched patients initiating SGLT2 inhibitor vs. DPP-4 inhibitor therapy stratified by HbA_{1c} levels.

eTable 8. Time-limited "intent-to-treat" analysis and secondary analyses of the primary effectiveness outcomes in 1:1 propensity score-matched patients initiating SGLT2 inhibitor vs. DPP-4 inhibitor therapy and stratified by HbA_{1c} levels

eTable 9. Number of events, incidence rates, relative and absolute estimates for sensitivity analysis of incident safety outcomes in 1:1 PS-matched initiators of SGLT2 inhibitor vs. DPP4 inhibitor therapy

This supplemental material has been provided by the authors to give readers additional information about their work.

© 2023 D'Andrea E et al. *JAMA Internal Medicine.*

eMethods

Feasibility Analyses

Before conducting the main effectiveness and safety analyses, we performed a feasibility analysis comparing baseline characteristics of eligible patients with vs. without an HbA1c value recorded within 3 months before cohort entry. The aim was to investigate whether patients with HbA1c value in the de-identified Optum® Clinformatics® Data Mart Database were representative of the full insured population by sharing similar baseline characteristics of those without an HbA1c value. Balance of baseline characteristics between the two sub-populations was assessed with standardized differences, with meaningful imbalances set at values higher than 10% (see eTable 1).

Sensitivity Analyses

We inspected the robustness of the main findings through sensitivity analyses. We explored potential informative censoring by carrying forward the exposure to the first-used medication for one year from cohort entry, without considering drug discontinuation or switching, i.e., mimicking an "intention-to-treat" approach. To assess consistency of treatment effect across subgroups of patients with and without history of cardiovascular disease (defined as a diagnosis of MI, angina, coronary atherosclerosis or other forms of chronic ischemic heart disease, coronary procedure, congestive heart failure, ischemic stroke, peripheral artery disease or surgery, recorded within six months before cohort entry), we tested the significance of an interaction term between exposure and cardiovascular disease history in our primary effectiveness outcome models. Our primary cohort included users of insulin, which is generally prescribed in routine care as treatment intensification (or third-line therapy) in patients with T2D at an advanced stage. Comparisons involving both second-line therapies (SGLT2i and DPP-4i) and a third-line therapy (insulin) may introduce time-lag bias, as insulin users can have a more severe diabetes. This bias is compounded by the fact that the insulin users had already started insulin before cohort entry, unlike the new users of SGLT2i or DPP-4i. To address potential time-lag bias and thus test the internal validity of the primary effectiveness findings, we excluded from the original cohort patients on insulin and required patients to receive metformin at cohort entry. Additionally, as CVOTs showed an increased HHF rate in patients receiving saxagliptin or alogliptin compared to placebo, which resulted in an FDA warning, we conducted a sensitivity analysis for the HHF outcome redefining the comparator group as sitagliptin only, which had a neutral effect on HHF in CVOTs. Finally, to address the potential for unmeasured confounding associated with the high risk for recurrence for certain safety outcomes, we excluded from the original study population patients with a recent history of fracture of the pelvis, hip, humerus, radius and ulna, genital mycotic infection and DKA events at baseline. All secondary cohorts built for the sensitivity analyses were stratified by HbA1c levels and within each HbA1c stratum, PS was re-estimated and matching re-performed. The definitions of the secondary cohorts are reported in eTable 2.

© 2023 D'Andrea E et al. *JAMA Internal Medicine.*

eTable 1. Baseline characteristics of eligible patients with vs. without a HbA_{1c} value within 3 months before cohort entry.

	Patients without HbA1c	Patients with HbA1c	St. Diff.
Number of patients	302,204	144,614	-
**Demographics and lifestyle risk factors**			
Age			
...mean (sd)	61.56 (12.52)	62.05 (12.37)	-0.04
...median [IQR]	63.00 [53, 70]	64.00 [53, 71]	--
Age Categories			
...18 - 39; n (%)	14,698 (4.9%)	6,496 (4.5%)	0.02
...40 - 44; n (%)	15,602 (5.2%)	6,938 (4.8%)	0.02
...45 - 49; n (%)	23,898 (7.9%)	11,167 (7.7%)	0.01
...50 - 54; n (%)	32,787 (10.8%)	14,947 (10.3%)	0.02
...55 - 59; n (%)	38,882 (12.9%)	17,784 (12.3%)	0.02
...60 - 64; n (%)	37,896 (12.5%)	17,402 (12.0%)	0.02
...65 - 69; n (%)	53,297 (17.6%)	27,360 (18.9%)	-0.03
...70 - 74; n (%)	40,592 (13.4%)	20,698 (14.3%)	-0.03
...75 - 79; n (%)	24,163 (8.0%)	12,171 (8.4%)	-0.01
...80 - 84; n (%)	13,915 (4.6%)	6,659 (4.6%)	0.00
...>= 85; n (%)	6,474 (2.1%)	2,992 (2.1%)	0.00
Male; n (%)	160,326 (53.1%)	76,358 (52.8%)	0.01
Race			
...White; n (%)	161,982 (53.6%)	71,372 (49.4%)	0.08
Quarter/Year of Cohort Entry			
...Q2 2013; n (%)	7,232 (2.4%)	2,743 (1.9%)	0.03
...Q3 2013; n (%)	8,375 (2.8%)	3,084 (2.1%)	0.05
...Q4 2013; n (%)	8,256 (2.7%)	3,116 (2.2%)	0.03
...Q1 2014; n (%)	7,410 (2.5%)	3,010 (2.1%)	0.03
...Q2 2014; n (%)	7,567 (2.5%)	3,006 (2.1%)	0.03
...Q3 2014; n (%)	8,497 (2.8%)	3,647 (2.5%)	0.02
...Q4 2014; n (%)	8,565 (2.8%)	3,865 (2.7%)	0.01
...Q1 2015; n (%)	7,573 (2.5%)	3,680 (2.5%)	0.00
...Q2 2015; n (%)	7,272 (2.4%)	3,799 (2.6%)	-0.01
...Q3 2015; n (%)	7,701 (2.5%)	3,859 (2.7%)	-0.01
...Q4 2015; n (%)	7,448 (2.5%)	3,437 (2.4%)	0.01
...Q1 2016; n (%)	7,163 (2.4%)	3,997 (2.8%)	-0.03
...Q2 2016; n (%)	6,482 (2.1%)	3,953 (2.7%)	-0.04
...Q3 2016; n (%)	8,701 (2.9%)	4,684 (3.2%)	-0.02
...Q4 2016; n (%)	8,566 (2.8%)	4,319 (3.0%)	-0.01
...Q1 2017; n (%)	9,363 (3.1%)	4,659 (3.2%)	-0.01
...Q2 2017; n (%)	8,368 (2.8%)	4,664 (3.2%)	-0.02
...Q3 2017; n (%)	10,109 (3.3%)	5,168 (3.6%)	-0.02
...Q4 2017; n (%)	9,261 (3.1%)	4,360 (3.0%)	0.01
...Q1 2018; n (%)	9,242 (3.1%)	4,585 (3.2%)	-0.01
...Q2 2018; n (%)	8,832 (2.9%)	4,683 (3.2%)	-0.02
...Q3 2018; n (%)	10,053 (3.3%)	4,598 (3.2%)	0.01
...Q4 2018; n (%)	9,745 (3.2%)	4,129 (2.9%)	0.02
...Q1 2019; n (%)	10,595 (3.5%)	4,895 (3.4%)	0.01

© 2023 D’Andrea E et al. *JAMA Internal Medicine.*

...Q2 2019; n (%)	9,583 (3.2%)	5,170 (3.6%)	-0.02
...Q3 2019; n (%)	10,815 (3.6%)	4,871 (3.4%)	0.01
...Q4 2019; n (%)	10,667 (3.5%)	4,610 (3.2%)	0.02
...Q1 2020; n (%)	11,306 (3.7%)	5,784 (4.0%)	-0.02
...Q2 2020; n (%)	9,409 (3.1%)	4,240 (2.9%)	0.01
...Q3 2020; n (%)	10,645 (3.5%)	5,665 (3.9%)	-0.02
...Q4 2020; n (%)	11,275 (3.7%)	5,650 (3.9%)	-0.01
...Q1 2021; n (%)	14,012 (4.6%)	6,151 (4.3%)	0.01
...Q2 2021; n (%)	12,116 (4.0%)	6,533 (4.5%)	-0.02
Obesity; n (%)	83,418 (27.6%)	44,312 (30.6%)	-0.07
**Burden of comorbidities**			
Combined comorbidity score, mean (sd)	1.00 (1.94)	1.14 (1.97)	-0.07
Frailty Score. mean (sd)	0.15 (0.04)	0.15 (0.04)	0.00
**Diabetes-related Comorbidities**			
Diabetic nephropathy; n (%)	40,797 (13.5%)	24,384 (16.9%)	-0.09
Diabetic retinopathy; n (%)	20,286 (6.7%)	10,786 (7.5%)	-0.03
Lower-limb amputations; n (%)	1,694 (0.6%)	750 (0.5%)	0.01
Diabetic ketoacidosis; n (%)	771 (0.3%)	275 (0.2%)	0.02
**Other comorbidities**			
Acute MI; n (%)	4,440 (1.5%)	1,536 (1.1%)	0.04
MI sequelae/old MI; n (%)	8,276 (2.7%)	3,507 (2.4%)	0.02
Stable angina; n (%)	9,431 (3.1%)	5,188 (3.6%)	-0.03
Unstable angina; n (%)	4,583 (1.5%)	1,792 (1.2%)	0.03
Coronary atherosclerosis; n (%)	45,856 (15.2%)	20,348 (14.1%)	0.03
Coronary procedure; n (%)	3,705 (1.2%)	1,312 (0.9%)	0.03
History of coronary procedure; n (%)	14,677 (4.9%)	6,000 (4.1%)	0.04
Ischemic stroke; n (%)	13,398 (4.4%)	6,159 (4.3%)	0.00
Congestive heart failure; n (%)	22,228 (7.4%)	9,657 (6.7%)	0.03
Acute Kidney Injury; n (%)	9,246 (3.1%)	3,736 (2.6%)	0.03
CKD Stage 3-4; n (%)	23,446 (7.8%)	14,471 (10.0%)	-0.08
Fractures; n (%)	2,692 (0.9%)	1,163 (0.8%)	0.01
Falls; n (%)	7,905 (2.6%)	3,309 (2.3%)	0.02
Mycotic infections	26,200 (8.7%)	13,222 (9.1%)	-0.01
**Diabetes treatment**			
Number of glucose-lowering medications at cohort entry, mean (sd)	1.21 (0.85)	1.24 (0.85)	-0.04
No use of glucose-lowering medications at baseline; n (%)	51,028 (16.9%)	21,618 (14.9%)	0.05
Metformin (concurrent use); n (%)	189,252 (62.6%)	93,843 (64.9%)	-0.05
Sulfonylureas - 2nd generation (concurrent use); n (%)	85,125 (28.2%)	42,549 (29.4%)	-0.03
GLP-1 RA (concurrent use); n (%)	24,823 (8.2%)	11,799 (8.2%)	0.00
Insulins (concurrent use); n (%)	47,038 (15.6%)	20,826 (14.4%)	0.03
**Other Common treatments**			
ACEi and ARBs; n (%)	202,159 (66.9%)	100,112 (69.2%)	-0.05
Beta blockers; n (%)	96,683 (32.0%)	44,899 (31.0%)	0.02
Calcium channel blockers; n (%)	75,576 (25.0%)	36,743 (25.4%)	-0.01
Statins; n (%)	197,579 (65.4%)	99,791 (69.0%)	-0.08
NSAIDs; n (%)	56,940 (18.8%)	27,215 (18.8%)	0.00
Opioids; n (%)	67,322 (22.3%)	29,225 (20.2%)	0.05

© 2023 D'Andrea E et al. *JAMA Internal Medicine.*

Antidepressants; n (%)	74,885 (24.8%)	31,932 (22.1%)	0.06
**Health Care Utilization Measures**			
Endocrinologist (Number of visits), mean (sd)	0.63 (2.66)	0.46 (1.86)	0.07
Cardiologist (Number of visits), mean (sd)	1.18 (3.80)	1.10 (3.54)	0.02
Electrocardiogram; n (%)	79,541 (26.3%)	40,353 (27.9%)	-0.04
Electrocardiogram (Number of tests), mean (sd)	0.59 (1.55)	0.54 (1.35)	0.03
ECG & Other cardiac imaging; n (%)	35,214 (11.7%)	15,712 (10.9%)	0.03
ECG & Other cardiac imaging (Number of tests), mean (sd)	0.54 (3.27)	0.41 (2.67)	0.04
Number of Hospitalization event, mean (sd)	0.09 (0.37)	0.07 (0.31)	0.06
Number of ED event, mean (sd)	0.19 (0.75)	0.18 (0.76)	0.01
Distinct medications ALL, mean (sd)	9.87 (5.23)	9.85 (5.02)	0.00
Distinct medications all BRAND, mean (sd)	2.19 (1.44)	2.16 (1.41)	0.02
Cost pharmacy out-of-pocket ($), mean (sd)	388.99 (429.95)	385.28 (428.48)	0.01
Abbreviations. St. Diff., standardized difference; SD, standard deviation; MI, myocardial infarction; CKD, chronic kidney disease; GLP-1 RA, Glucagon-like peptide-1 receptor agonists; ARBs, Angiotensin II Receptor Blockers; ACEis, Angiotensin Converting Enzyme inhibitors; NSAIDs, nonsteroidal anti-inflammatory drugs; ECG, electrocardiogram; ED, emergency department.			

© 2023 D'Andrea E et al. *JAMA Internal Medicine.*

eFigure 1. Study design diagram and definition of the cohort

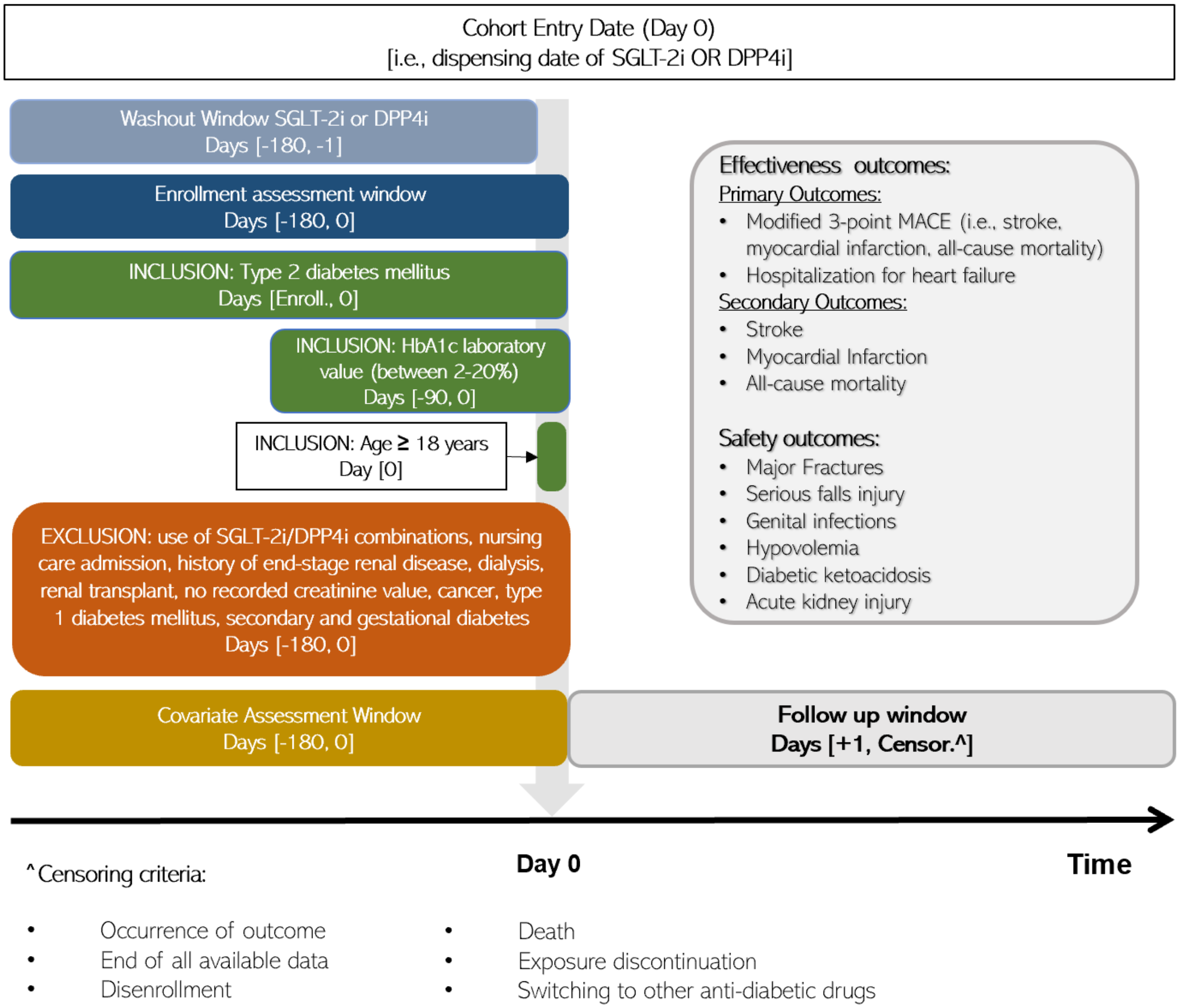

Abbreviations. MACE, major adverse cardiovascular event; SGLT-2i, Sodium-Glucose Cotransporter-2 Inhibitors; DPP4i, dipeptidyl peptidase 4 Inhibitors.

© 2023 D'Andrea E et al. *JAMA Internal Medicine.*

eTable 2. Definitions of inclusion and exclusion criteria

Inclusion criteria	Codes	Setting/Position
HbA1c laboratory records	Loinc: 17855-8, 17856-6, 71875-9, 41995-2, 43150-2, 4548-4, 4549-2, 55454-3, 74246-0	--
Type 2 diabetes mellitus	ICD 9 diagnosis: 250.00, 250.02, 250.10, 250.12, 250.20, 250.22, 250.30, 250.32, 250.40, 250.42, 250.50, 250.52, 250.60, 250.62, 250.70, 250.72, 250.80, 250.82, 250.90, 250.92<br>ICD 10 diagnosis: E11.x	Inpatient or outpatient setting, any position
Metformin maintenance therapy*	Generic Name: Metformin	2 fills recorded within 6 months before and including cohort entry
**Exclusion criteria**	**Codes**	
End-stage renal disease (dialysis or renal transplant)	ICD-9 diagnosis: 585.5, 585.6, 996.81, V42.0, V45.1x, V56.xx<br>ICD-9 procedure: 39.95, 54.98, 55.6x<br>ICD-10 diagnosis: N18.5, N18.6, R88.0, T82.41x, T82.42x, T82.43x, T82.49x, T85.611x, T85.621x, T85.631x, T85.71x, T86.1x, Y84.1, Z48.22, Z49.xx, Z91.15, Z94.0, Z99.2<br>ICD-10 procedure: 0TY00Zx, 0TY10Zx, 3E1M39Z, 5A1Dx0Z<br>HCPCS/CPT: 50360, 50365, 90920, 90921, 90924, 90925, 90935, 90937, 90939, 90940, 90945, 90947, 90957, 90958, 90959, 90960, 90961, 90962, 90965, 90966, 90969, 90970, 90989, 90993, 90999, 90997, 99512, 99559, 99512, G0257, G0314, G0315, G0316, G0317, G0318, G0319, G0322, G0323, G0326, G0327, S9335, S9339	Inpatient or outpatient setting, any position
Malignancy (excluding non-melanoma sin cancer)	ICD 9 diagnosis: 140.x-209.x (except 173.x, non-melanoma skin cancer and 209.4x, 209.5x, 209.6x)<br>ICD 10 diagnosis: C00.x-C96, D03.x, D45 (except C44.x, non-melanoma skin cancer)	Inpatient or outpatient setting, any position
Nursing home	Claims in SNF dataset<br>CPT codes: 99301, 99302, 99303, 99311, 99312, 99313, 99315, 99316, 99379, 99380, G0066<br>Place of service code: 31 (skilled nursing facility), 32 (nursing facility), 33 (custodial care facility)	Inpatient or outpatient setting, any position
Type 1 diabetes mellitus	ICD 9 diagnosis: 250.01, 250.03, 250.11, 250.13, 250.21, 250.23, 250.31, 250.33, 250.41, 250.43, 250.51, 250.53, 250.61, 250.63, 250.71, 250.73, 250.81, 250.83, 250.91, 250.93<br>ICD 10 diagnosis: E10.x	Inpatient or outpatient setting, any position
Secondary and gestational diabetes	ICD 9 diagnosis: 249.x, 648.8x<br>ICD 10 diagnosis: E08.x, E09.x, O24.4x, O99.81	Inpatient or outpatient setting, any position
Insulin*	Generic name: Insulin	1 fill recorded within 6 months before and including cohort entry
Mycotic infections**	ICD 9 diagnosis: 110.x-118.x<br>ICD 10 diagnosis: B35.x-B49.x	Inpatient or outpatient setting, any position
History of fracture of the hip, pelvis, humerus, radius, or ulna**	**Humerus fracture:**<br>ICD-9 diagnosis: 812.xx, 733.11<br>ICD 10 diagnosis: M80.02x, M80.82x, M84.42x, M84.62x, S42.5x<br>ICD-9 procedure: 78.52, 79.01, 79.11, 79.21, 79.31, 79.61<br>ICD-10 procedure: 0PHCx, 0PHDx, 0PHFx 0PHGx, OPSDx, OPSEx, OPSFx, OPSGx<br>CPT-4: 23600, 23605, 23610, 23615, 23620, 23625, 23630, 23665, 23670, 23680, 24500, 24505, 24510, 24515, 24530, 24531, 24535, 24536, 24538, 24540, 24542, 24545, 24560, 24565, 24575, 24586, 24587, 24588, 24516<br>**Radius/ulna fracture:**	Inpatient or outpatient setting, any position

© 2023 D’Andrea E et al. *JAMA Internal Medicine.*

	<u>ICD-9 diagnosis</u>: 813.xx, 733.12<br><u>ICD 10 diagnosis</u>: M80.03x, M80.83x, M84.43x, M84.63x, S52.x<br><u>ICD-9 procedure</u>: 78.53, 79.02, 79.12, 79.22, 79.32, 79.62<br><u>ICD-10 procedure</u>: 0PHHx, 0PHKx, 0PHJx, 0PHLx, 0PSHx, 0PSKx, 0PSJx, 0PSLx<br><u>CPT-4</u>: 24620, 24635, 24650, 24655, 24660, 24665, 24666, 24670, 24680, 24685, 25500, 25505, 25510, 25515, 25530, 25535, 25540, 25545, 25560, 25565, 25570, 25575, 25600, 25605, 25610, 25611, 25615, 25620, 25650<br>**Hip fracture:**<br><u>ICD-9 diagnosis</u>: 820.x (excl. 820.01, 820.11), 821.x (excl. 821.32, 820.11), 733.14, 733.15, 733.96, 733.97<br><u>ICD 10 diagnosis</u>: M80.05x, M80.85x, M84.35x, (excl. M84.350x), M84.45x, (excl. M84.454x), M84.65x (excl. M84.650x), M84.75x, S72.x (excl. S72.02x, S72.44x)<br>**Pelvic fracture:**<br><u>ICD-9 diagnosis</u>: 808.xx, 733.98<br><u>ICD 10 diagnosis</u>: M84.350x, M84.454x, M84.650x, S32.3x-S32.9x<br><u>HCPCS/CPT</u>: 27193, 27194, 27200, 27202, 27215, 27216, 27217, 27218, 27220, 27222, 27226, 27227, 27228, G0412, G0413, G0414, G0415	
Diabetic ketoacidosis**	<u>ICD-9 diagnosis</u>: 250.1x<br><u>ICD-10 diagnosis</u>: E10.1x, E11.1x, E13.1x	Inpatient or outpatient setting, any position
*Inclusion and exclusion criteria applied to the secondary cohort of the comparative effectiveness analysis.<br>** Exclusion criteria applied only to the secondary cohort of the comparative safety outcomes analysis.		

© 2023 D’Andrea E et al. *JAMA Internal Medicine.*

eTable 3. Outcome definitions

Outcome	Component	Diagnosis and/or Procedure Codes	Setting/Position
**Effectiveness outcomes**			
Modified MACE	Myocardial Infarction	ICD-9 diagnosis: 410.x<br>ICD-10 diagnosis: I21.x (excluding I21.9, I21.Ax)	Inpatient, primary or secondary position
	Stroke	ICD-9 diagnosis: 430, 431, 433.x1, 434.x1, 436<br>ICD-10 diagnosis: I60.x, I61.x, I63.x, I67.89	Inpatient, primary position
	All-cause mortality	Information on mortality was available through four sources: Centers for Medicare and Medicaid Services, Social Security Administration Master Death Files, in-hospital deaths, and death as a reason for insurance discontinuation.	
All-cause mortality	--	Same definition provided for the MACE outcome	
Myocardial Infarction	--	Same definition provided for the MACE outcome	
Stroke	--	Same definition provided for the MACE outcome	
Hospitalized Heart Failure	--	ICD-9 diagnosis: 428.xx, 398.91, 402.x1, 404.x1, 404.x3<br>ICD-10 diagnosis: I09.81, I11.0, I13.0, I13.2, I50.xxx	Inpatient, any position
**Safety outcomes**			
Major fractures	Humerus	Case qualifying (CQ) = 1 Diagnosis (ICD-9: 812.x, 733.11; ICD-10: M80.02xA, M80.82xA, M84.42xA, M84.62xA, S42.xxxA, S42.xxxB, S42.xxxC)<br>**OR**<br>CQ = 2 Diagnosis (ICD-9: 812.x, 733.11; ICD-10: M80.02xA, M80.82xA, M84.42xA, M84.62xA, S42.xxxA, S42.xxxB, S42.xxxC) **AND (overlapping)** Procedure (ICD-9: 78.52, 79.01, 79.11, 79.21, 79.31, 79.61; ICD-10: 0PHCx, 0PHDx, 0PHFx 0PHGx, OPSDx, OPSEx, OPSFx, OPSGx; CPT-4: 23600, 23605, 23610, 23615, 23620, 23625, 23630, 23665, 23670, 23680, 24500, 24505, 24510, 24515, 24530, 24531, 24535, 24536, 24538, 24540, 24542, 24545, 24560, 24565, 24575, 24586, 24587, 24588, 24516)	Inpatient, any position<br><br>Non-inpatient, any position
	Radius/ulna	CQ = 1 Diagnosis (ICD-9: 813.x, 733.12; ICD-10: M80.03xA, M80.83xA, M84.43xA, M84.63xA, S52.xxxA, S52.xxxB, S52.xxxC)<br>**OR**<br>CQ = 2 Diagnosis (ICD-9: 813.x, 733.12; ICD-10: M80.03xA, M80.83xA, M84.43xA, M84.63xA, S52.xxxA, S52.xxxB, S52.xxxC) **AND (overlapping)** Procedure (ICD-9: 78.53, 79.02, 79.12, 79.22, 79.32, 79.62; ICD-10: 0PHHx, 0PHKx, 0PHJx, 0PHLx, 0PSHx, 0PSKx, 0PSJx, 0PSLx; CPT-4: 24620, 24635, 24650, 24655, 24660, 24665, 24666, 24670, 24680, 24685, 25500, 25505, 25510, 25515, 25530, 25535, 25540, 25545, 25560, 25565, 25570, 25575, 25600, 25605, 25610, 25611, 25615, 25620, 25650)	Inpatient, any position<br><br>Non-inpatient, any position
	Hip	CQ = 1 Diagnosis [ICD-9: 820.x (excl. 820.01, 820.11), 821.x (excl. 821.32, 820.11), 733.14, 733.15, 733.96, 733.97; ICD 10: M80.05xA, M80.85xA, M84.35xA (excl. M84.350x), M84.45xA (excl. M84.454x), M84.65xA (excl. M84.650x), M84.75xA, S72.xxxA, S72.xxxB, S72.xxxC (excl. S72.02x, S72.44x)]<br>**OR**<br>CQ = 2 Diagnosis [ICD-9: 820.x (excl. 820.01, 820.11), 821.x (excl. 821.32, 820.11), 733.14, 733.15, 733.96, 733.97; ICD 10: M80.05xA, M80.85xA, M84.35xA (excl. M84.350x), M84.45xA (excl. M84.454x), M84.65xA (excl. M84.650x), M84.75xA, S72.xxxA, S72.xxxB, S72.xxxC (excl. S72.02x, S72.44x)] **AND (overlapping)** Procedure (ICD-9: 78.55, 79.05, 79.15, 79.25, 79.35, 79.65; ICD-10: 0QH6x, 0QH7x, 0QH8x, 0QH9x, 0QHBx, 0QHCx, 0QS6x, 0QS7x, 0QS8x, 0QS9x, 0QSBx, 0QSCx; CPT: 27230, 27232, 27235, 27236, 27238, 27240, 27244, 27245, 27246, 27248, 27267, 27268, 27269, 27125, 27130, 27500, 27503, 27508, 27509, 27513, 27501, 27502, 27506, 27507, 27514, 27254)	Inpatient, any position<br><br>Non-inpatient, any position

© 2023 D'Andrea E et al. *JAMA Internal Medicine.*

	Pelvis	CQ = 1 Diagnosis (ICD-9: 808.x, 733.98; ICD-10: ICD 10 diagnosis: M84.350xA, M84.454xA, M84.650xA, S32.3xxA, S32.3xxB, S32.4xxA, S32.4xxB, S32.5xxA, S32.5xxB, S32.6xxA, S32.6xxB, S32.8xxA, S32.8xxB, S32.9xxA, S32.9xxB)<br>**OR**<br>CQ = 2 Diagnosis (ICD-9: 808.x, 733.98; ICD-10: ICD 10 diagnosis: M84.350xA, M84.454xA, M84.650xA, S32.3xxA, S32.3xxB, S32.4xxA, S32.4xxB, S32.5xxA, S32.5xxB, S32.6xxA, S32.6xxB, S32.8xxA, S32.8xxB, S32.9xxA, S32.9xxB) **AND (overlapping)** Procedures (CPT/ HCPCS: 27193, 27194, 27200, 27202, 27215, 27216, 27217, 27218, 27220, 27222, 27226, 27227, 27228, G0412, G0413, G0414, G0415)	Inpatient, any position<br><br>Non-inpatient, any position
Serious falls injuries	Injury AND Falls	ICD-9 diagnosis: non-pathological fractures = 800.00-806.19, 807.0x-807.2x, 808.0x-8089x, 810.00-814.19, 818.0x-825.1x, 827.0x-829.1x; Brain injury = 852.00-852.39; Dislocation of the hip, knee, shoulder, elbow, or jaw = 830.0x-832.19, 835.00-835.13, 836.30-836.60<br>ICD-10 diagnosis: non-pathological fractures = S02.xxxA, S06.xxxA, S14.xxxA, S22.xxxA, S32.xxxA, S42.xxxA, S49.xxxA, S52.xxxA, S62.xxxA, S79.xxxA, S72.xxxA, S82.xxxA, S89.xxxA, S99.xxxA, S02.xxxB, S06.xxxB, S14.xxxB, S22.xxxB, S32.xxxB, S42.xxxB, S49.xxxB, S52.xxxB, S62.xxxB, S79.xxxB, S72.xxxB, S82.xxxB, S89.xxxB, S99.xxxB; Brain injury = S06.5xxA, S06.6xxA; Dislocation of the hip, knee, shoulder, elbow, or jaw = S03.0xxA, S43.0xxA, S43.1xxA, S43.2xxA, S43.3xxA, S53.0xxA, S53.1xxA, S73.0xxA, S83.0xxA, S83.1xxA<br>**AND (overlapping)**<br>ICD-9 diagnosis: E880.0x-E888.9x<br>ICD-10 diagnosis: V00.xxxA, W00.xxxA-W19.xxxA	Inpatient or ED setting, any position
	Injury NOT Motor vehicle accidents	ICD-9 and ICD-10 diagnosis: See codes for non-pathological; Brain injury; Dislocation of the hip, knee, shoulder, elbow, or jaw reported above<br>**NOT (not occurring +/- 15 days)**<br>ICD-9 diagnosis: E810.x-E819.x<br>ICD-10 diagnosis:V20-V79	Inpatient or ED setting, any position
Hypovolemia	--	ICD-9 diagnosis: 276.50, 276.52, 276.51<br>ICD-10 diagnosis: E86.9, E86.1, R57.1, E86.0	Inpatient setting and ED visits, any position
Genital infections^	--	ICD-9 diagnosis: 112.1, 616.1x, 112.2, 607.1, 112.2, 605<br>ICD-10 diagnosis: B37.3, N77.1, N76.0-N76.3, B37.49, B37.42, N48.1, N47.6, B37.49, N47.x (except N47.0, N47.6)	Any setting, any position
Diabetic ketoacidosis	--	ICD-9 diagnosis: 250.1x<br>ICD-10 diagnosis: E10.1x, E11.1x, E13.1x	Inpatient, primary position
Acute kidney injury	--	ICD-9 diagnosis: 584.x<br>ICD-10 diagnosis: N17.x	Inpatient, any position
Lower-limb amputations	--	ICD-9 procedure: 84.1x (excluding 84.18, 84.19)<br>ICD-10 procedure: 0Y.6x (excluding 0Y.62, 0Y.63, 0Y.64)<br>CPT: 27590, 27591, 27592, 27880, 27881, 27882, 27884, 27886, 27888, 27889, 28800, 28805, 28810, 28820, 28825, 27594, 27596, 27598	Any setting, any position

Abbreviations: MACE, Major Adverse Cardiovascular Events; ICD, International Classification of Diseases; CPT, Current Procedural Terminology.

© 2023 D'Andrea E et al. *JAMA Internal Medicine.*

eTable 4. Baseline characteristics of patients with HbA_{1c} < 7.5% initiating SGLT2 inhibitor vs. DPP4 inhibitor therapy before and after 1:1 PS-matching

Baseline characteristics	DPP-4 inhibitor	SGLT2 inhibitor	St. Diff.	DPP-4 inhibitor	SGLT2 inhibitor	St. Diff.
Number of patients	27,783	16,316	-	12,026	12,026	-
Demographics						
Age						
...mean (sd)	66.68 (11.58)	61.06 (11.54)	0.49	62.34 (11.31)	62.47 (11.40)	-0.01
...median [IQR]	68 [60, 75]	62 [53, 69]	-	65 [55, 70]	64 [55, 71]	-
Age squared						
...mean (sd)	4,580.34 (1,474.32)	3,861.37 (1,367.07)	0.51	4,013.50 (1,355.67)	4,032.10 (1,374.78)	-0.01
...median [IQR]	4,624 [3,600, 5,625]	3,844 [2,809, 4,761]	-	4,225 [3,025, 4,900]	4,096 [3,025, 5,041]	-
Age Categories						
...18 - 39; n (%)	600 (2.2%)	701 (4.3%)	-0.12	424 (3.5%)	403 (3.4%)	0.01
...40 - 44; n (%)	701 (2.5%)	763 (4.7%)	-0.12	490 (4.1%)	485 (4.0%)	0.01
...45 - 49; n (%)	1,201 (4.3%)	1,324 (8.1%)	-0.16	800 (6.7%)	852 (7.1%)	-0.02
...50 - 54; n (%)	1,818 (6.5%)	1,763 (10.8%)	-0.15	1,186 (9.9%)	1,129 (9.4%)	0.02
...55 - 59; n (%)	2,574 (9.3%)	2,224 (13.6%)	-0.14	1,548 (12.9%)	1,522 (12.7%)	0.01
...60 - 64; n (%)	2,867 (10.3%)	2,326 (14.3%)	-0.12	1,513 (12.6%)	1,690 (14.1%)	-0.04
...65 - 69; n (%)	5,760 (20.7%)	3,239 (19.9%)	0.02	2,705 (22.5%)	2,505 (20.8%)	0.04
...70 - 74; n (%)	5,173 (18.6%)	2,261 (13.9%)	0.13	1,920 (16.0%)	1,877 (15.6%)	0.01
...75 - 79; n (%)	3,616 (13.0%)	1,145 (7.0%)	0.20	952 (7.9%)	1,020 (8.5%)	-0.02
...80 - 84; n (%)	2,334 (8.4%)	427 (2.6%)	0.26	365 (3.0%)	401 (3.3%)	-0.02
...>= 85; n (%)	1,139 (4.1%)	143 (0.9%)	0.21	123 (1.0%)	142 (1.2%)	-0.02
Gender						
...Male; n (%)	12,942 (46.6%)	8,466 (51.9%)	-0.11	6,047 (50.3%)	6,034 (50.2%)	0.00
...Female; n (%)	14,841 (53.4%)	7,850 (48.1%)	0.11	5,979 (49.7%)	5,992 (49.8%)	0.00
Geographic region						
...Northeast; n (%)	3,369 (12.1%)	1,531 (9.4%)	0.09	1,170 (9.7%)	1,172 (9.7%)	0.00
...South; n (%)	15,581 (56.1%)	9,743 (59.7%)	-0.07	7,152 (59.5%)	7,164 (59.6%)	0.00
...Midwest; n (%)	1,874 (6.7%)	1,354 (8.3%)	-0.06	934 (7.8%)	949 (7.9%)	0.00
...West; n (%)	6,959 (25.0%)	3,688 (22.6%)	0.06	2,770 (23.0%)	2,741 (22.8%)	0.00
Race categories						
...White; n (%)	13,584 (48.9%)	9,144 (56.0%)	-0.14	6,436 (53.5%)	6,463 (53.7%)	0.00
...Black; n (%)	3,762 (13.5%)	1,902 (11.7%)	0.05	1,471 (12.2%)	1,483 (12.3%)	0.00
...Asian; n (%)	2,679 (9.6%)	1,098 (6.7%)	0.11	906 (7.5%)	914 (7.6%)	0.00
...Hispanic; n (%)	6,244 (22.5%)	3,252 (19.9%)	0.06	2,513 (20.9%)	2,482 (20.6%)	0.01
...Other or unknown; n (%)	1,514 (5.4%)	920 (5.6%)	-0.01	700 (5.8%)	684 (5.7%)	0.00
Quarter/Year of Cohort Entry Date						
...Q2 2013; n (%)	837 (3.0%)	50 (0.3%)	0.21	35 (0.3%)	50 (0.4%)	-0.02
...Q3 2013; n (%)	951 (3.4%)	70 (0.4%)	0.22	54 (0.4%)	68 (0.6%)	-0.03
...Q4 2013; n (%)	871 (3.1%)	117 (0.7%)	0.18	92 (0.8%)	109 (0.9%)	-0.01
...Q1 2014; n (%)	788 (2.8%)	123 (0.8%)	0.15	96 (0.8%)	115 (1.0%)	-0.02
...Q2 2014; n (%)	700 (2.5%)	181 (1.1%)	0.11	155 (1.3%)	160 (1.3%)	0.00
...Q3 2014; n (%)	817 (2.9%)	258 (1.6%)	0.09	214 (1.8%)	223 (1.9%)	-0.01
...Q4 2014; n (%)	791 (2.8%)	309 (1.9%)	0.06	270 (2.2%)	266 (2.2%)	0.00
...Q1 2015; n (%)	714 (2.6%)	316 (1.9%)	0.05	276 (2.3%)	276 (2.3%)	0.00
...Q2 2015; n (%)	723 (2.6%)	354 (2.2%)	0.03	298 (2.5%)	294 (2.4%)	0.01
...Q3 2015; n (%)	831 (3.0%)	344 (2.1%)	0.06	308 (2.6%)	306 (2.5%)	0.01
...Q4 2015; n (%)	723 (2.6%)	340 (2.1%)	0.03	296 (2.5%)	292 (2.4%)	0.01
...Q1 2016; n (%)	836 (3.0%)	299 (1.8%)	0.08	254 (2.1%)	262 (2.2%)	-0.01

© 2023 D'Andrea E et al. *JAMA Internal Medicine.*

...Q2 2016; n (%)	772 (2.8%)	320 (2.0%)	0.05	295 (2.5%)	287 (2.4%)	0.01
...Q3 2016; n (%)	984 (3.5%)	444 (2.7%)	0.05	377 (3.1%)	389 (3.2%)	-0.01
...Q4 2016; n (%)	854 (3.1%)	400 (2.5%)	0.04	354 (2.9%)	338 (2.8%)	0.01
...Q1 2017; n (%)	905 (3.3%)	442 (2.7%)	0.04	379 (3.2%)	370 (3.1%)	0.01
...Q2 2017; n (%)	864 (3.1%)	449 (2.8%)	0.02	391 (3.3%)	382 (3.2%)	0.01
...Q3 2017; n (%)	1,040 (3.7%)	569 (3.5%)	0.01	470 (3.9%)	467 (3.9%)	0.00
...Q4 2017; n (%)	935 (3.4%)	491 (3.0%)	0.02	427 (3.6%)	409 (3.4%)	0.01
...Q1 2018; n (%)	982 (3.5%)	457 (2.8%)	0.04	382 (3.2%)	375 (3.1%)	0.01
...Q2 2018; n (%)	922 (3.3%)	503 (3.1%)	0.01	409 (3.4%)	396 (3.3%)	0.01
...Q3 2018; n (%)	836 (3.0%)	516 (3.2%)	-0.01	407 (3.4%)	409 (3.4%)	0.00
...Q4 2018; n (%)	863 (3.1%)	478 (2.9%)	0.01	385 (3.2%)	378 (3.1%)	0.01
...Q1 2019; n (%)	929 (3.3%)	576 (3.5%)	-0.01	469 (3.9%)	443 (3.7%)	0.01
...Q2 2019; n (%)	887 (3.2%)	625 (3.8%)	-0.03	479 (4.0%)	483 (4.0%)	0.00
...Q3 2019; n (%)	797 (2.9%)	634 (3.9%)	-0.06	447 (3.7%)	437 (3.6%)	0.01
...Q4 2019; n (%)	743 (2.7%)	734 (4.5%)	-0.10	462 (3.8%)	476 (4.0%)	-0.01
...Q1 2020; n (%)	860 (3.1%)	884 (5.4%)	-0.11	591 (4.9%)	564 (4.7%)	0.01
...Q2 2020; n (%)	707 (2.5%)	567 (3.5%)	-0.06	416 (3.5%)	421 (3.5%)	0.00
...Q3 2020; n (%)	878 (3.2%)	915 (5.6%)	-0.12	585 (4.9%)	600 (5.0%)	0.00
...Q4 2020; n (%)	799 (2.9%)	1,039 (6.4%)	-0.17	620 (5.2%)	601 (5.0%)	0.01
...Q1 2021; n (%)	867 (3.1%)	1,131 (6.9%)	-0.18	656 (5.5%)	691 (5.7%)	-0.01
...Q2 2021; n (%)	777 (2.8%)	1,381 (8.5%)	-0.25	677 (5.6%)	689 (5.7%)	0.00
Burden of comorbidities						
Com. comorbidity score, mean (sd)	1.47 (2.26)	1.12 (1.97)	0.17	1.12 (1.99)	1.12 (2.02)	0.00
Frailty Score, mean (sd)	0.15 (0.05)	0.15 (0.04)	0.00	0.15 (0.04)	0.15 (0.04)	0.00
Frailty Score squared, mean (sd)	0.03 (0.02)	0.02 (0.01)	0.63	0.02 (0.01)	0.02 (0.01)	0.00
Frailty score; n (%)						
...Robust (< 0.15)	15,341 (55.2%)	10,243 (62.8%)	-0.15	7,463 (62.1%)	7,518 (62.5%)	-0.01
...Prefrail (0.15 - 0.25)	11,296 (40.7%)	5,723 (35.1%)	0.12	4,271 (35.5%)	4,241 (35.3%)	0.00
...Frail (>= 0.25)	1,146 (4.1%)	350 (2.1%)	0.12	292 (2.4%)	267 (2.2%)	0.01
Lifestyle factors, n (%)						
Overweight	2,551 (9.2%)	1,490 (9.1%)	0.00	1,162 (9.7%)	1,141 (9.5%)	0.01
Obesity	6,889 (24.8%)	6,254 (38.3%)	-0.29	3,959 (32.9%)	3,995 (33.2%)	-0.01
Smoking	3,498 (12.6%)	2,312 (14.2%)	-0.05	1,664 (13.8%)	1,623 (13.5%)	0.01
Diabetes-related conditions, n (%)						
Diabetic nephropathy	5,948 (21.4%)	2,353 (14.4%)	0.18	1,788 (14.9%)	1,818 (15.1%)	-0.01
Diabetic retinopathy	1,803 (6.5%)	964 (5.9%)	0.02	677 (5.6%)	672 (5.6%)	0.00
Diabetes with other ophthalmic manifestations	1,614 (5.8%)	649 (4.0%)	0.08	538 (4.5%)	526 (4.4%)	0.00
Diabetic neuropathy	5,288 (19.0%)	2,938 (18.0%)	0.03	2,119 (17.6%)	2,130 (17.7%)	0.00
Diabetes with peripheral circulatory disorders	449 (1.6%)	70 (0.4%)	0.12	45 (0.4%)	58 (0.5%)	-0.01
Diabetic foot	398 (1.4%)	208 (1.3%)	0.01	144 (1.2%)	148 (1.2%)	0.00
Infection of lower extremities	566 (2.0%)	325 (2.0%)	0.00	212 (1.8%)	211 (1.8%)	0.00
Lower-limb amputations	120 (0.4%)	66 (0.4%)	0.00	40 (0.3%)	44 (0.4%)	-0.02
Erectile dysfunction	708 (2.5%)	586 (3.6%)	-0.06	390 (3.2%)	386 (3.2%)	0.00
Hypoglycemia	3,814 (13.7%)	2,446 (15.0%)	-0.04	1,741 (14.5%)	1,755 (14.6%)	0.00
Hyperglycemia	7,072 (25.5%)	5,833 (35.8%)	-0.22	3,824 (31.8%)	3,856 (32.1%)	-0.01
Diabetic ketoacidosis	35 (0.1%)	22 (0.1%)	0.00	17 (0.1%)	14 (0.1%)	0.00
Hyperosmolar hyperglycemic nonketotic syndrome	130 (0.5%)	88 (0.5%)	0.00	63 (0.5%)	60 (0.5%)	0.00
Diabetes with other complications	1,655 (6.0%)	1,046 (6.4%)	-0.02	734 (6.1%)	745 (6.2%)	0.00
Diabetes mellitus without mention of complications	22,896 (82.4%)	12,870 (78.9%)	0.09	9,643 (80.2%)	9,571 (79.6%)	0.01

© 2023 D'Andrea E et al. *JAMA Internal Medicine.*

Diabetes treatment						
Number of anti-DM medications on index date						
...mean (sd)	0.98 (0.77)	1.13 (0.88)	-0.18	1.02 (0.79)	1.02 (0.81)	0.00
...median [IQR]	1.00 [0.00, 1.00]	1.00 [1.00, 2.00]	-	1.00 [0.00, 1.00]	1.00 [0.00, 1.00]	-
No use of anti-DM medications in prior 180 days; n (%)	6,053 (21.8%)	2,610 (16.0%)	0.15	2,276 (18.9%)	2,266 (18.8%)	0.00
Metformin (any use); n (%)	19,324 (69.6%)	11,989 (73.5%)	-0.09	8,815 (73.3%)	8,777 (73.0%)	0.01
Metformin (concurrent use); n (%)	16,545 (59.6%)	10,120 (62.0%)	-0.05	7,573 (63.0%)	7,400 (61.5%)	0.03
Sulfonylureas - 2nd gen. (any use); n (%)	7,866 (28.3%)	3,709 (22.7%)	0.13	2,917 (24.3%)	2,910 (24.2%)	0.00
Sulfonylureas - 2nd gen. (concurrent use); n (%)	6,334 (22.8%)	2,863 (17.5%)	0.13	2,311 (19.2%)	2,280 (19.0%)	0.01
Thiazolidinediones (any use); n (%)	2,313 (8.3%)	1,444 (8.9%)	-0.02	1,064 (8.8%)	1,022 (8.5%)	0.01
Thiazolidinediones (concurrent use); n (%)	1,811 (6.5%)	1,113 (6.8%)	-0.01	847 (7.0%)	796 (6.6%)	0.02
GLP-1RA (any use); n (%)	1,011 (3.6%)	3,243 (19.9%)	-0.52	919 (7.6%)	1,023 (8.5%)	-0.03
GLP-1RA (concurrent use); n (%)	580 (2.1%)	2,464 (15.1%)	-0.48	537 (4.5%)	754 (6.3%)	-0.08
Insulins (any use); n (%)	2,233 (8.0%)	2,378 (14.6%)	-0.21	1,215 (10.1%)	1,265 (10.5%)	-0.01
Insulins (concurrent use); n (%)	1,536 (5.5%)	1,750 (10.7%)	-0.19	838 (7.0%)	939 (7.8%)	-0.03
Long term use of insulin; n (%)	1,430 (5.1%)	1,541 (9.4%)	-0.17	774 (6.4%)	800 (6.7%)	-0.01
Miscellaneous anti-DM medications; n (%)	428 (1.5%)	188 (1.2%)	0.03	138 (1.1%)	135 (1.1%)	0.00
Other comorbidities at baseline, n (%)						
Hypertension	22,467 (80.9%)	12,786 (78.4%)	0.06	9,476 (78.8%)	9,425 (78.4%)	0.01
Hyperlipidemia	22,240 (80.0%)	13,276 (81.4%)	-0.04	9,678 (80.5%)	9,699 (80.7%)	-0.01
Acute MI	321 (1.2%)	248 (1.5%)	-0.03	153 (1.3%)	148 (1.2%)	0.01
MI sequelae/old MI	729 (2.6%)	477 (2.9%)	-0.02	311 (2.6%)	304 (2.5%)	0.01
Stable angina	1,152 (4.1%)	795 (4.9%)	-0.04	557 (4.6%)	544 (4.5%)	0.00
ACS unstable angina	413 (1.5%)	275 (1.7%)	-0.02	190 (1.6%)	170 (1.4%)	0.02
Coronary atherosclerosis	4,589 (16.5%)	2,689 (16.5%)	0.00	1,816 (15.1%)	1,875 (15.6%)	-0.01
Coronary procedure	285 (1.0%)	235 (1.4%)	-0.04	147 (1.2%)	143 (1.2%)	0.00
History of coronary procedure	1,275 (4.6%)	870 (5.3%)	-0.03	575 (4.8%)	563 (4.7%)	0.00
Congestive heart failure	2,378 (8.6%)	1,284 (7.9%)	0.03	861 (7.2%)	874 (7.3%)	0.00
Cardiomyopathy	816 (2.9%)	523 (3.2%)	-0.02	335 (2.8%)	335 (2.8%)	0.00
Atrial fibrillation	2,005 (7.2%)	1,005 (6.2%)	0.04	699 (5.8%)	727 (6.0%)	-0.01
Cardiac conduction disorders	770 (2.8%)	489 (3.0%)	-0.01	342 (2.8%)	330 (2.7%)	0.01
Other cardiac dysrhythmia	2,080 (7.5%)	1,141 (7.0%)	0.02	785 (6.5%)	828 (6.9%)	-0.02
Valve disorders	1,982 (7.1%)	1,066 (6.5%)	0.02	708 (5.9%)	784 (6.5%)	-0.02
Other cardiovascular disease	2,494 (9.0%)	1,525 (9.3%)	-0.01	968 (8.0%)	1,078 (9.0%)	-0.04
Ischemic stroke	1,610 (5.8%)	693 (4.2%)	0.07	527 (4.4%)	527 (4.4%)	0.00
TIA	382 (1.4%)	168 (1.0%)	0.04	102 (0.8%)	129 (1.1%)	-0.03
Other cerebrovascular conditions	1,226 (4.4%)	515 (3.2%)	0.06	384 (3.2%)	399 (3.3%)	-0.01
PAD and generalized/unspecified atherosclerosis	2,167 (7.8%)	1,007 (6.2%)	0.06	755 (6.3%)	762 (6.3%)	0.00
Acute Kidney Injury	1,235 (4.4%)	368 (2.3%)	0.12	302 (2.5%)	286 (2.4%)	0.01
CKD	4,930 (17.7%)	1,156 (7.1%)	0.33	1,653 (13.7%)	1,671 (13.9%)	-0.01
CKD Stage 1-2	1,664 (6.0%)	766 (4.7%)	0.06	596 (5.0%)	603 (5.0%)	0.00
CKD Stage 3-4	4,930 (17.7%)	1,156 (7.1%)	0.33	974 (8.1%)	1,009 (8.4%)	-0.01
CKD unspecified	1,672 (6.0%)	425 (2.6%)	0.17	341 (2.8%)	341 (2.8%)	0.00
Miscellaneous renal disease	2,313 (8.3%)	733 (4.5%)	0.16	580 (4.8%)	588 (4.9%)	0.00
Major bleeding	1,646 (5.9%)	834 (5.1%)	0.04	630 (5.2%)	627 (5.2%)	0.00
Urinary tract infections	2,842 (10.2%)	1,137 (7.0%)	0.11	889 (7.4%)	905 (7.5%)	0.00
Mycotic infections	2,638 (9.5%)	1,389 (8.5%)	0.03	975 (8.1%)	1,013 (8.4%)	-0.01
Disorder of Electrolyte	1,883 (6.8%)	856 (5.2%)	0.07	619 (5.1%)	644 (5.4%)	-0.01

© 2023 D'Andrea E et al. *JAMA Internal Medicine.*

Disorders of fluid balance	791 (2.8%)	318 (1.9%)	0.06	246 (2.0%)	243 (2.0%)	0.00
Edema	1,916 (6.9%)	1,009 (6.2%)	0.03	734 (6.1%)	737 (6.1%)	0.00
COPD	2,576 (9.3%)	1,153 (7.1%)	0.08	912 (7.6%)	910 (7.6%)	0.00
Asthma	1,764 (6.3%)	1,067 (6.5%)	-0.01	769 (6.4%)	763 (6.3%)	0.00
Obstructive sleep apnea	3,098 (11.2%)	2,757 (16.9%)	-0.16	1,717 (14.3%)	1,748 (14.5%)	-0.01
Pneumonia	762 (2.7%)	350 (2.1%)	0.04	265 (2.2%)	250 (2.1%)	0.01
Osteoarthritis	4,410 (15.9%)	2,319 (14.2%)	0.05	1,780 (14.8%)	1,756 (14.6%)	0.01
Osteoporosis without fractures	1,442 (5.2%)	511 (3.1%)	0.11	430 (3.6%)	444 (3.7%)	-0.01
Fractures	288 (1.0%)	138 (0.8%)	0.02	99 (0.8%)	105 (0.9%)	-0.01
Falls	802 (2.9%)	353 (2.2%)	0.04	260 (2.2%)	272 (2.3%)	-0.01
NASH/NAFLD	1,113 (4.0%)	949 (5.8%)	-0.08	628 (5.2%)	631 (5.2%)	0.00
Liver disease	987 (3.6%)	535 (3.3%)	0.02	391 (3.3%)	399 (3.3%)	0.00
Depression	3,247 (11.7%)	1,920 (11.8%)	0.00	1,453 (12.1%)	1,413 (11.7%)	0.01
Anxiety and sleep disorders	3,879 (14.0%)	2,699 (16.5%)	-0.07	1,928 (16.0%)	1,904 (15.8%)	0.01
Dementia	906 (3.3%)	213 (1.3%)	0.13	201 (1.7%)	181 (1.5%)	0.02
Psychosis	236 (0.8%)	89 (0.5%)	0.04	77 (0.6%)	70 (0.6%)	0.00
Delirium	194 (0.7%)	67 (0.4%)	0.04	45 (0.4%)	51 (0.4%)	0.00
Other medication use, n (%)						
ACEi and ARBs	19,726 (71.0%)	11,145 (68.3%)	0.06	8,296 (69.0%)	8,248 (68.6%)	0.01
Beta blockers	10,001 (36.0%)	5,387 (33.0%)	0.06	3,897 (32.4%)	3,899 (32.4%)	0.00
Calcium channel blockers	8,406 (30.3%)	4,124 (25.3%)	0.11	3,142 (26.1%)	3,137 (26.1%)	0.00
Nitrates and other antianginal agents	1,328 (4.8%)	712 (4.4%)	0.02	523 (4.3%)	507 (4.2%)	0.00
Thiazide and thiazide-like diuretics	3,442 (12.4%)	1,963 (12.0%)	0.01	1,439 (12.0%)	1,439 (12.0%)	0.00
Loop diuretics	3,387 (12.2%)	1,700 (10.4%)	0.06	1,204 (10.0%)	1,202 (10.0%)	0.00
MRA (potassium-sparing diuretics)	953 (3.4%)	711 (4.4%)	-0.05	432 (3.6%)	460 (3.8%)	-0.01
Other K-sparing diuretics and antihypertensive medications	2,791 (10.0%)	1,116 (6.8%)	0.12	871 (7.2%)	900 (7.5%)	-0.01
Antiarrhythmics	478 (1.7%)	244 (1.5%)	0.02	184 (1.5%)	186 (1.5%)	0.00
Anticoagulants (oral)	1,752 (6.3%)	952 (5.8%)	0.02	674 (5.6%)	669 (5.6%)	0.00
Anticoagulants (injectables)	73 (0.3%)	40 (0.2%)	0.02	21 (0.2%)	30 (0.2%)	0.00
Antiplatelet agents	2,580 (9.3%)	1,476 (9.0%)	0.01	1,039 (8.6%)	1,031 (8.6%)	0.00
Statins	19,655 (70.7%)	11,646 (71.4%)	-0.02	8,490 (70.6%)	8,509 (70.8%)	0.00
PCSK9 inhibitors and other lipid-lowering agents	4,152 (14.9%)	2,441 (15.0%)	0.00	1,753 (14.6%)	1,767 (14.7%)	0.00
COPD & asthma medications	4,009 (14.4%)	2,279 (14.0%)	0.01	1,676 (13.9%)	1,699 (14.1%)	-0.01
Corticosteroids (oral)	3,004 (10.8%)	1,731 (10.6%)	0.01	1,269 (10.6%)	1,292 (10.7%)	0.00
Antiosteoporosis agents	1,263 (4.5%)	408 (2.5%)	0.11	349 (2.9%)	361 (3.0%)	-0.01
NSAIDs	5,324 (19.2%)	3,258 (20.0%)	-0.02	2,394 (19.9%)	2,402 (20.0%)	0.00
Opioids	6,254 (22.5%)	3,227 (19.8%)	0.07	2,449 (20.4%)	2,463 (20.5%)	0.00
Gabapentinoids	3,827 (13.8%)	2,151 (13.2%)	0.02	1,614 (13.4%)	1,606 (13.4%)	0.00
UTI antibiotics	3,759 (13.5%)	1,708 (10.5%)	0.09	1,315 (10.9%)	1,333 (11.1%)	-0.01
Antidepressants	6,588 (23.7%)	4,124 (25.3%)	-0.04	3,023 (25.1%)	2,976 (24.7%)	0.01
Anxiolytics/hypnotics	1,724 (6.2%)	1,060 (6.5%)	-0.01	788 (6.6%)	755 (6.3%)	0.01
Benzodiazepines	3,187 (11.5%)	1,709 (10.5%)	0.03	1,341 (11.2%)	1,326 (11.0%)	0.01
Antipsychotics	793 (2.9%)	429 (2.6%)	0.02	333 (2.8%)	326 (2.7%)	0.01
Antiparkinsonian medications	617 (2.2%)	347 (2.1%)	0.01	260 (2.2%)	238 (2.0%)	0.01
Dementia medications	753 (2.7%)	156 (1.0%)	0.13	177 (1.5%)	129 (1.1%)	0.04
Measures of health care use						
Internist; n (%)	26,008 (93.6%)	14,715 (90.2%)	0.12	11,076 (92.1%)	10,996 (91.4%)	0.03
Internist (-30 days to CED); n (%)	20,309 (73.1%)	11,131 (68.2%)	0.11	8,579 (71.3%)	8,487 (70.6%)	0.02
Internist (-180 days to -31 days before CED); n (%)	23,211 (83.5%)	12,904 (79.1%)	0.11	9,757 (81.1%)	9,615 (80.0%)	0.03

© 2023 D'Andrea E et al. *JAMA Internal Medicine.*

Internist (Number of visits)						
...mean (sd)	12.53 (16.89)	10.15 (15.10)	0.15	10.95 (14.95)	10.79 (15.58)	0.01
Endocrinologist; n (%)	3,041 (10.9%)	3,029 (18.6%)	-0.22	1,598 (13.3%)	1,686 (14.0%)	-0.02
Endocrinologist (-30 days to CED); n (%)	1,961 (7.1%)	2,196 (13.5%)	-0.21	1,122 (9.3%)	1,150 (9.6%)	-0.01
Endocrinologist (-180 days to -31 days before CED); n (%)	2,180 (7.8%)	2,194 (13.4%)	-0.18	1,142 (9.5%)	1,219 (10.1%)	-0.02
Endocrinologist (Number of visits)						
...mean (sd)	0.41 (1.83)	0.70 (2.26)	-0.14	0.50 (2.02)	0.51 (1.90)	-0.01
Cardiologist; n (%)	7,298 (26.3%)	4,181 (25.6%)	0.02	2,934 (24.4%)	2,964 (24.6%)	0.00
Cardiologist (-30 days to CED); n (%)	2,845 (10.2%)	1,749 (10.7%)	-0.02	1,168 (9.7%)	1,201 (10.0%)	-0.01
Cardiologist (-180 days to -31 days before CED); n (%)	6,116 (22.0%)	3,476 (21.3%)	0.02	2,450 (20.4%)	2,457 (20.4%)	0.00
Cardiologist (Number of visits)						
...mean (sd)	1.39 (3.99)	1.44 (4.25)	-0.01	1.31 (3.87)	1.35 (4.17)	-0.01
Electrocardiogram; n (%)	9,104 (32.8%)	4,814 (29.5%)	0.07	3,582 (29.8%)	3,551 (29.5%)	0.01
Electrocardiogram (Number of tests)						
...mean (sd)	0.67 (1.62)	0.60 (1.43)	0.05	0.60 (1.45)	0.59 (1.40)	0.01
ECG & Other cardiac imaging; n (%)	3,745 (13.5%)	2,166 (13.3%)	0.01	1,464 (12.2%)	1,526 (12.7%)	-0.02
ECG & Other cardiac imaging (Number of tests)						
...mean (sd)	0.50 (3.00)	0.57 (3.24)	-0.02	0.49 (2.88)	0.53 (3.18)	-0.01
Cardiovascular stress test; n (%)	1,541 (5.5%)	896 (5.5%)	0.00	667 (5.5%)	645 (5.4%)	0.00
HbA1c test order (Number of tests)						
...mean (sd)	1.59 (0.82)	1.63 (0.81)	-0.05	1.60 (0.81)	1.60 (0.81)	0.00
Glucose test and monitoring; n (%)	6,077 (21.9%)	3,522 (21.6%)	0.01	2,426 (20.2%)	2,506 (20.8%)	-0.01
Glucose test and monitoring (Number of tests)						
...mean (sd)	0.48 (1.38)	0.46 (1.41)	0.01	0.42 (1.23)	0.43 (1.15)	-0.01
Microalbuminuria/proteinuria test order; n (%)	14,749 (53.1%)	8,786 (53.8%)	-0.01	6,376 (53.0%)	6,317 (52.5%)	0.01
Microalbuminuria/proteinuria test order (Number of tests)						
...mean (sd)	0.71 (0.84)	0.71 (0.79)	0.00	0.69 (0.80)	0.69 (0.79)	0.00
Metabolic or renal/creatinine panel test order; n (%)	24,742 (89.1%)	14,001 (85.8%)	0.10	10,475 (87.1%)	10,347 (86.0%)	0.03
Metabolic or renal/creatinine panel test order (Number of tests)						
...mean (sd)	1.87 (1.62)	1.75 (1.52)	0.08	1.73 (1.42)	1.73 (1.49)	0.00
Lipid test order; n (%)	23,838 (85.8%)	14,192 (87.0%)	-0.04	10,454 (86.9%)	10,422 (86.7%)	0.01
Lipid test order (Number of tests)						
...mean (sd)	1.36 (0.97)	1.37 (0.94)	-0.01	1.36 (0.92)	1.36 (0.94)	0.00
Uric acid test order; n (%)	3,684 (13.3%)	1,702 (10.4%)	0.09	1,305 (10.9%)	1,288 (10.7%)	0.01
Vitamin D test order; n (%)	8,855 (31.9%)	4,909 (30.1%)	0.04	3,597 (29.9%)	3,651 (30.4%)	-0.01
PTH test order; n (%)	2,112 (7.6%)	853 (5.2%)	0.10	705 (5.9%)	656 (5.5%)	0.02
Hospitalization event (Number of)						
...mean (sd)	0.10 (0.38)	0.07 (0.31)	0.09	0.07 (0.32)	0.07 (0.31)	0.00
LOS (-180 to -31 days before CED)						
...mean (sd)	0.30 (2.05)	0.25 (1.82)	0.03	0.25 (1.86)	0.24 (1.85)	0.01
LOS (-30 days to CED)						
...mean (sd)	0.17 (2.54)	0.07 (0.73)	0.05	0.06 (0.68)	0.07 (0.78)	-0.01
ED event (Number of)						
...mean (sd)	0.24 (0.88)	0.14 (0.59)	0.13	0.15 (0.64)	0.16 (0.63)	-0.02
Distinct medications ALL						
...mean (sd)	10.22 (5.11)	10.18 (5.23)	0.01	9.95 (5.15)	9.94 (5.10)	0.00

© 2023 D'Andrea E et al. *JAMA Internal Medicine.*

Distinct medications all BRAND						
...mean (sd)	2.12 (1.37)	2.30 (1.47)	-0.13	2.09 (1.35)	2.11 (1.35)	-0.01
Cost pharmacy out-of-pocket ($)						
...mean (sd)	382.93 (432.92)	442.84 (472.80)	-0.13	397.20 (504.64)	401.41 (410.92)	-0.01
Flu vaccine; n (%)	5,851 (21.1%)	3,254 (19.9%)	0.03	2,480 (20.6%)	2,426 (20.2%)	0.01
Pneumococcal vaccine; n (%)	1,680 (6.0%)	931 (5.7%)	0.01	727 (6.0%)	706 (5.9%)	0.00
Breast mammography or MRI; n (%)	3,808 (13.7%)	2,217 (13.6%)	0.00	1,692 (14.1%)	1,678 (14.0%)	0.00
Prostate DRE or PSA; n (%)	5,094 (18.3%)	3,199 (19.6%)	-0.03	2,428 (20.2%)	2,310 (19.2%)	0.03
Colonoscopy; n (%)	1,404 (5.1%)	790 (4.8%)	0.01	631 (5.2%)	609 (5.1%)	0.00
Pap smear; n (%)	1,137 (4.1%)	835 (5.1%)	-0.05	585 (4.9%)	580 (4.8%)	0.00
Bone mineral density; n (%)	1,410 (5.1%)	615 (3.8%)	0.06	491 (4.1%)	496 (4.1%)	0.00
Laboratory results						
Lab value HbA1c (%)						
...mean (sd)	6.77 (0.59)	6.83 (0.62)	-0.10	6.81 (0.62)	6.82 (0.59)	-0.02
...median [IQR]	6.80 [6.40, 7.20]	6.90 [6.45, 7.20]	-	6.85 [6.40, 7.20]	6.90 [6.45, 7.20]	-
eGFR (w/o race)						
...mean (sd)	67.93 (24.51)	76.11 (24.11)	-0.34	75.14 (23.64)	74.86 (23.64)	0.01
...median [IQR]	69.46 [49.14, 87.77]	79.63 [61.31, 93.96]	-	78.78 [59.69, 92.71]	78.00 [60.06, 92.57]	-
Lab result number - Creatinine						
...mean (sd)	1.18 (1.45)	1.12 (1.30)	0.04	1.10 (1.42)	1.11 (1.28)	-0.01
...median [IQR]	0.98 [0.80, 1.27]	0.92 [0.77, 1.12]	-	0.92 [0.77, 1.14]	0.92 [0.77, 1.13]	-
Lab result number - uACR						
...mean (sd)	135.58 (615.05)	104.82 (497.79)	0.05	101.51 (538.60)	108.41 (523.43)	-0.01
...Missing; n (%)	17,532 (63.1%)	9,997 (61.3%)	0.04	7,453 (62.0%)	7,533 (62.6%)	-0.01
Lab result number - Total cholesterol						
...mean (sd)	171.63 (47.59)	170.89 (50.19)	0.02	170.99 (47.16)	171.89 (50.28)	-0.02
...Missing; n (%)	3,893 (14.0%)	2,291 (14.0%)	0.00	1,563 (13.0%)	1,672 (13.9%)	-0.03
Lab result number - LDL cholesterol						
...mean (sd)	93.42 (41.21)	92.90 (42.81)	0.01	93.41 (40.90)	93.16 (42.52)	0.01
...Missing; n (%)	4,080 (14.7%)	2,453 (15.0%)	-0.01	1,680 (14.0%)	1,805 (15.0%)	-0.03
Lab result number - HDL cholesterol						
...mean (sd)	50.50 (19.54)	49.90 (22.02)	0.03	49.23 (19.33)	50.19 (21.48)	-0.05
...Missing; n (%)	4,246 (15.3%)	2,544 (15.6%)	-0.01	1,741 (14.5%)	1,844 (15.3%)	-0.02
Lab result number - Triglycerides						
...mean (sd)	165.52 (111.66)	181.33 (139.24)	-0.13	170.98 (119.12)	178.78 (133.96)	-0.06
...Missing; n (%)	3,911 (14.1%)	2,222 (13.6%)	0.01	1,563 (13.0%)	1,626 (13.5%)	-0.01
Lab result value - Glucose						
...mean (sd)	141.35 (71.61)	150.01 (98.60)	-0.10	143.99 (75.24)	147.97 (95.56)	-0.05
...Missing; n (%)	816 (2.9%)	441 (2.7%)	0.01	312 (2.6%)	316 (2.6%)	0.00

Abbreviations. St. Diff., standardized difference; SD, standard deviation; GLP-1 RA, Glucagon-like peptide-1 receptor agonists; MI, myocardial infarction; TIA, transient ischemic attack; peripheral artery disease; NASH/NAFLD, Nonalcoholic steatohepatitis/Nonalcoholic fatty liver disease; COPD, chronic obstructive pulmonary disease; CKD, chronic kidney disease; ARBs, Angiotensin II Receptor Blockers; ACEis, Angiotensin Converting Enzyme inhibitors; MRA, mineralocorticoid receptor antagonist; PCSK9, Proprotein convertase subtilisin/kexin type 9; NSAIDs, nonsteroidal anti-inflammatory drugs; MRI, magnetic resonance imaging; DRE, digital rectal examination; PSA, prostate-specific antigen; ED, emergency department, LOS, length of stay; ECG, electrocardiogram; eGFR, estimated glomerular filtration rate; uACR, urine albumin-creatinine ratio; LDL, low-density lipoprotein; HDL, high-density lipoprotein.

© 2023 D’Andrea E et al. *JAMA Internal Medicine.*

eTable 5. Baseline characteristics of patients with HbA_{1c} between 7.5% and 9% initiating SGLT2 inhibitor vs. DPP4 inhibitor therapy before and after 1:1 PS-matching

Baseline characteristics	DPP-4 inhibitor	SGLT2 inhibitor	St. Diff.	DPP-4 inhibitor	SGLT2 inhibitor	St. Diff.
Number of patients	30,674	22,312	-	16,145	16,145	-
Demographics						
Age						
...mean (sd)	64.86 (11.87)	60.80 (11.38)	0.35	61.96 (11.41)	62.03 (11.46)	-0.01
...median [IQR]	67 [57, 73]	62 [53, 69]	-	64 [54, 70]	64 [54, 70]	-
Age squared						
...mean (sd)	4,347.85 (1,480.73)	3,826.14 (1,349.10)	0.37	3,969.46 (1,367.59)	3,979.61 (1,376.03)	-0.01
...median [IQR]	4,489 [3,249, 5,329]	3,844 [2,809, 4,761]	-	4,096 [2,916, 4,900]	4,096 [2,916, 4,900]	-
Age Categories						
...18 - 39; n (%)	843 (2.7%)	899 (4.0%)	-0.07	566 (3.5%)	587 (3.6%)	-0.01
...40 - 44; n (%)	962 (3.1%)	1,142 (5.1%)	-0.10	672 (4.2%)	745 (4.6%)	-0.02
...45 - 49; n (%)	1,817 (5.9%)	1,813 (8.1%)	-0.09	1,256 (7.8%)	1,111 (6.9%)	0.03
...50 - 54; n (%)	2,536 (8.3%)	2,589 (11.6%)	-0.11	1,720 (10.7%)	1,663 (10.3%)	0.01
...55 - 59; n (%)	3,183 (10.4%)	3,129 (14.0%)	-0.11	2,003 (12.4%)	2,078 (12.9%)	-0.02
...60 - 64; n (%)	3,356 (10.9%)	3,165 (14.2%)	-0.10	2,081 (12.9%)	2,220 (13.8%)	-0.03
...65 - 69; n (%)	6,337 (20.7%)	4,354 (19.5%)	0.03	3,494 (21.6%)	3,304 (20.5%)	0.03
...70 - 74; n (%)	5,316 (17.3%)	2,999 (13.4%)	0.11	2,460 (15.2%)	2,436 (15.1%)	0.00
...75 - 79; n (%)	3,376 (11.0%)	1,480 (6.6%)	0.16	1,235 (7.6%)	1,295 (8.0%)	-0.01
...80 - 84; n (%)	2,031 (6.6%)	558 (2.5%)	0.20	483 (3.0%)	524 (3.2%)	-0.01
...>= 85; n (%)	917 (3.0%)	184 (0.8%)	0.16	175 (1.1%)	182 (1.1%)	0.00
Gender						
…Male; n (%)	15,567 (50.7%)	12,554 (56.3%)	-0.11	8,784 (54.4%)	8,794 (54.5%)	0.00
…Female; n (%)	15,107 (49.3%)	9,758 (43.7%)	0.11	7,361 (45.6%)	7,351 (45.5%)	0.00
Geographic region						
...Northeast; n (%)	3,275 (10.7%)	1,892 (8.5%)	0.07	1,425 (8.8%)	1,430 (8.9%)	0.00
...South; n (%)	16,973 (55.3%)	13,102 (58.7%)	-0.07	9,378 (58.1%)	9,346 (57.9%)	0.00
...Midwest; n (%)	2,445 (8.0%)	2,076 (9.3%)	-0.05	1,459 (9.0%)	1,440 (8.9%)	0.00
...West; n (%)	7,981 (26.0%)	5,242 (23.5%)	0.06	3,883 (24.1%)	3,929 (24.3%)	0.00
Race categories						
...White; n (%)	14,686 (47.9%)	12,186 (54.6%)	-0.13	8,380 (51.9%)	8,358 (51.8%)	0.00
...Black; n (%)	3,851 (12.6%)	2,495 (11.2%)	0.04	1,890 (11.7%)	1,930 (12.0%)	-0.01
...Asian; n (%)	2,548 (8.3%)	1,329 (6.0%)	0.09	1,113 (6.9%)	1,092 (6.8%)	0.00
...Hispanic; n (%)	7,838 (25.6%)	4,970 (22.3%)	0.08	3,768 (23.3%)	3,807 (23.6%)	-0.01
...Other or unknown; n (%)	1,751 (5.7%)	1,332 (6.0%)	-0.01	994 (6.2%)	958 (5.9%)	0.01
Quarter/Year of Cohort Entry Date						
...Q2 2013; n (%)	949 (3.1%)	64 (0.3%)	0.22	37 (0.2%)	64 (0.4%)	-0.04
...Q3 2013; n (%)	942 (3.1%)	155 (0.7%)	0.18	117 (0.7%)	145 (0.9%)	-0.02
...Q4 2013; n (%)	1,000 (3.3%)	167 (0.7%)	0.19	137 (0.8%)	159 (1.0%)	-0.02
...Q1 2014; n (%)	934 (3.0%)	173 (0.8%)	0.16	149 (0.9%)	159 (1.0%)	-0.01
...Q2 2014; n (%)	830 (2.7%)	286 (1.3%)	0.10	237 (1.5%)	244 (1.5%)	0.00
...Q3 2014; n (%)	961 (3.1%)	376 (1.7%)	0.09	307 (1.9%)	323 (2.0%)	-0.01
...Q4 2014; n (%)	992 (3.2%)	475 (2.1%)	0.07	388 (2.4%)	403 (2.5%)	-0.01
...Q1 2015; n (%)	810 (2.6%)	546 (2.4%)	0.01	454 (2.8%)	443 (2.7%)	0.01
...Q2 2015; n (%)	846 (2.8%)	527 (2.4%)	0.03	451 (2.8%)	429 (2.7%)	0.01
...Q3 2015; n (%)	919 (3.0%)	495 (2.2%)	0.05	427 (2.6%)	417 (2.6%)	0.00
...Q4 2015; n (%)	786 (2.6%)	465 (2.1%)	0.03	396 (2.5%)	391 (2.4%)	0.01
...Q1 2016; n (%)	976 (3.2%)	491 (2.2%)	0.06	443 (2.7%)	411 (2.5%)	0.01

© 2023 D'Andrea E et al. *JAMA Internal Medicine.*

...Q2 2016; n (%)	975 (3.2%)	510 (2.3%)	0.06	473 (2.9%)	432 (2.7%)	0.01
...Q3 2016; n (%)	1,103 (3.6%)	631 (2.8%)	0.05	516 (3.2%)	539 (3.3%)	-0.01
...Q4 2016; n (%)	999 (3.3%)	626 (2.8%)	0.03	562 (3.5%)	524 (3.2%)	0.02
...Q1 2017; n (%)	1,092 (3.6%)	654 (2.9%)	0.04	560 (3.5%)	557 (3.4%)	0.01
...Q2 2017; n (%)	1,081 (3.5%)	643 (2.9%)	0.03	557 (3.4%)	536 (3.3%)	0.01
...Q3 2017; n (%)	1,104 (3.6%)	750 (3.4%)	0.01	615 (3.8%)	606 (3.8%)	0.00
...Q4 2017; n (%)	963 (3.1%)	603 (2.7%)	0.02	501 (3.1%)	500 (3.1%)	0.00
...Q1 2018; n (%)	1,008 (3.3%)	658 (2.9%)	0.02	541 (3.4%)	538 (3.3%)	0.01
...Q2 2018; n (%)	1,023 (3.3%)	666 (3.0%)	0.02	549 (3.4%)	530 (3.3%)	0.01
...Q3 2018; n (%)	961 (3.1%)	743 (3.3%)	-0.01	569 (3.5%)	580 (3.6%)	-0.01
...Q4 2018; n (%)	827 (2.7%)	634 (2.8%)	-0.01	509 (3.2%)	498 (3.1%)	0.01
...Q1 2019; n (%)	943 (3.1%)	824 (3.7%)	-0.03	616 (3.8%)	607 (3.8%)	0.00
...Q2 2019; n (%)	955 (3.1%)	904 (4.1%)	-0.05	644 (4.0%)	649 (4.0%)	0.00
...Q3 2019; n (%)	901 (2.9%)	937 (4.2%)	-0.07	638 (4.0%)	638 (4.0%)	0.00
...Q4 2019; n (%)	744 (2.4%)	947 (4.2%)	-0.10	579 (3.6%)	585 (3.6%)	0.00
...Q1 2020; n (%)	906 (3.0%)	1,228 (5.5%)	-0.12	741 (4.6%)	735 (4.6%)	0.00
...Q2 2020; n (%)	760 (2.5%)	865 (3.9%)	-0.08	573 (3.5%)	591 (3.7%)	-0.01
...Q3 2020; n (%)	924 (3.0%)	1,157 (5.2%)	-0.11	713 (4.4%)	700 (4.3%)	0.00
...Q4 2020; n (%)	823 (2.7%)	1,213 (5.4%)	-0.14	679 (4.2%)	700 (4.3%)	0.00
...Q1 2021; n (%)	867 (2.8%)	1,399 (6.3%)	-0.17	761 (4.7%)	772 (4.8%)	0.00
...Q2 2021; n (%)	770 (2.5%)	1,500 (6.7%)	-0.20	706 (4.4%)	740 (4.6%)	-0.01
Burden of comorbidities						
Com. comorbidity score, mean (sd)	1.19 (2.03)	1.01 (1.80)	0.09	1.00 (1.82)	1.01 (1.86)	-0.01
Frailty Score, mean (sd)	0.15 (0.04)	0.14 (0.04)	0.25	0.14 (0.04)	0.14 (0.04)	0.00
Frailty Score squared, mean (sd)	0.02 (0.02)	0.02 (0.01)	0.00	0.02 (0.01)	0.02 (0.01)	0.00
Frailty score; n (%)						
...< 0.15	18,690 (60.9%)	14,686 (65.8%)	-0.10	10,530 (65.2%)	10,575 (65.5%)	-0.01
...0.15 - 0.25	11,163 (36.4%)	7,259 (32.5%)	0.08	5,338 (33.1%)	5,273 (32.7%)	0.01
...>= 0.25	821 (2.7%)	367 (1.6%)	0.08	277 (1.7%)	297 (1.8%)	-0.01
Lifestyle factors, n (%)						
Overweight	2,485 (8.1%)	1,864 (8.4%)	-0.01	1,351 (8.4%)	1,372 (8.5%)	0.00
Obesity	8,178 (26.7%)	8,107 (36.3%)	-0.21	5,209 (32.3%)	5,198 (32.2%)	0.00
Smoking	3,714 (12.1%)	2,897 (13.0%)	-0.03	2,036 (12.6%)	2,031 (12.6%)	0.00
Diabetes-related conditions, n (%)						
Diabetic nephropathy	5,884 (19.2%)	3,350 (15.0%)	0.11	2,460 (15.2%)	2,477 (15.3%)	0.00
Diabetic retinopathy	2,473 (8.1%)	1,842 (8.3%)	-0.01	1,261 (7.8%)	1,267 (7.8%)	0.00
Diabetes with other ophthalmic manifestations	1,686 (5.5%)	983 (4.4%)	0.05	746 (4.6%)	739 (4.6%)	0.00
Diabetic neuropathy	6,128 (20.0%)	4,494 (20.1%)	0.00	3,141 (19.5%)	3,089 (19.1%)	0.01
Diabetes with peripheral circulatory disorders	534 (1.7%)	133 (0.6%)	0.10	126 (0.8%)	117 (0.7%)	0.01
Diabetic foot	445 (1.5%)	330 (1.5%)	0.00	216 (1.3%)	221 (1.4%)	-0.01
Infection of lower extremities	652 (2.1%)	470 (2.1%)	0.00	315 (2.0%)	320 (2.0%)	0.00
Lower-limb amputations	146 (0.5%)	104 (0.5%)	0.00	76 (0.5%)	73 (0.5%)	0.00
Erectile dysfunction	921 (3.0%)	845 (3.8%)	-0.04	556 (3.4%)	539 (3.3%)	0.01
Hypoglycemia	4,124 (13.4%)	3,420 (15.3%)	-0.05	2,395 (14.8%)	2,375 (14.7%)	0.00
Hyperglycemia	9,957 (32.5%)	9,770 (43.8%)	-0.23	6,318 (39.1%)	6,302 (39.0%)	0.00
Diabetic ketoacidosis	42 (0.1%)	32 (0.1%)	0.00	19 (0.1%)	21 (0.1%)	0.00
Hyperosmolar hyperglycemic nonketotic syndrome	137 (0.4%)	89 (0.4%)	0.00	71 (0.4%)	59 (0.4%)	0.00
Diabetes with other complications	1,734 (5.7%)	1,533 (6.9%)	-0.05	996 (6.2%)	991 (6.1%)	0.00
Diabetes mellitus without mention of complications	24,867 (81.1%)	16,980 (76.1%)	0.12	12,590 (78.0%)	12,600 (78.0%)	0.00

© 2023 D'Andrea E et al. *JAMA Internal Medicine.*

Diabetes treatment						
Number of anti-DM medications on index date						
...mean (sd)	1.24 (0.79)	1.40 (0.92)	-0.19	1.27 (0.80)	1.28 (0.85)	-0.01
...median [IQR]	1.00 [1.00, 2.00]	1.00 [1.00, 2.00]	-	1.00 [1.00, 2.00]	1.00 [1.00, 2.00]	-
No use of anti-DM medications in prior 180 days; n (%)	3,843 (12.5%)	1,898 (8.5%)	0.13	1,687 (10.4%)	1,699 (10.5%)	0.00
Metformin (any use); n (%)	23,323 (76.0%)	17,420 (78.1%)	-0.05	12,657 (78.4%)	12,615 (78.1%)	0.01
Metformin (concurrent use); n (%)	20,337 (66.3%)	14,915 (66.8%)	-0.01	11,062 (68.5%)	10,788 (66.8%)	0.04
Sulfonylureas - 2nd gen. (any use); n (%)	13,498 (44.0%)	8,316 (37.3%)	0.14	6,422 (39.8%)	6,424 (39.8%)	0.00
Sulfonylureas - 2nd gen. (concurrent use); n (%)	11,174 (36.4%)	6,756 (30.3%)	0.13	5,300 (32.8%)	5,269 (32.6%)	0.00
Thiazolidinediones (any use); n (%)	2,386 (7.8%)	2,033 (9.1%)	-0.05	1,369 (8.5%)	1,398 (8.7%)	-0.01
Thiazolidinediones (concurrent use); n (%)	1,886 (6.1%)	1,599 (7.2%)	-0.04	1,096 (6.8%)	1,111 (6.9%)	0.00
GLP-1RA (any use); n (%)	1,313 (4.3%)	4,740 (21.2%)	-0.52	1,234 (7.6%)	1,360 (8.4%)	-0.03
GLP-1RA (concurrent use); n (%)	802 (2.6%)	3,637 (16.3%)	-0.48	760 (4.7%)	1,024 (6.3%)	-0.07
Insulins (any use); n (%)	4,483 (14.6%)	5,421 (24.3%)	-0.25	2,911 (18.0%)	2,951 (18.3%)	-0.01
Insulins (concurrent use); n (%)	3,405 (11.1%)	4,226 (18.9%)	-0.22	2,199 (13.6%)	2,268 (14.0%)	-0.01
Long term use of insulin; n (%)	2,286 (7.5%)	2,847 (12.8%)	-0.18	1,491 (9.2%)	1,498 (9.3%)	0.00
Miscellaneous anti-DM medications; n (%)	548 (1.8%)	297 (1.3%)	0.04	232 (1.4%)	230 (1.4%)	0.00
Other comorbidities at baseline, n (%)						
Hypertension	24,135 (78.7%)	17,258 (77.3%)	0.03	12,454 (77.1%)	12,478 (77.3%)	0.00
Hyperlipidemia	24,139 (78.7%)	17,972 (80.5%)	-0.04	12,829 (79.5%)	12,789 (79.2%)	0.01
Acute MI	330 (1.1%)	229 (1.0%)	0.01	156 (1.0%)	157 (1.0%)	0.00
MI sequelae/old MI	775 (2.5%)	494 (2.2%)	0.02	335 (2.1%)	365 (2.3%)	-0.01
Stable angina	1,053 (3.4%)	828 (3.7%)	-0.02	535 (3.3%)	557 (3.4%)	-0.01
ACS unstable angina	348 (1.1%)	270 (1.2%)	-0.01	185 (1.1%)	177 (1.1%)	0.00
Coronary atherosclerosis	4,539 (14.8%)	3,235 (14.5%)	0.01	2,261 (14.0%)	2,262 (14.0%)	0.00
Coronary procedure	250 (0.8%)	201 (0.9%)	-0.01	132 (0.8%)	126 (0.8%)	0.00
History of coronary procedure	1,303 (4.2%)	1,000 (4.5%)	-0.01	669 (4.1%)	680 (4.2%)	-0.01
Congestive heart failure	2,093 (6.8%)	1,325 (5.9%)	0.04	929 (5.8%)	951 (5.9%)	0.00
Cardiomyopathy	707 (2.3%)	520 (2.3%)	0.00	370 (2.3%)	354 (2.2%)	0.01
Atrial fibrillation	1,725 (5.6%)	1,127 (5.1%)	0.02	780 (4.8%)	794 (4.9%)	0.00
Cardiac conduction disorders	667 (2.2%)	452 (2.0%)	0.01	313 (1.9%)	308 (1.9%)	0.00
Other cardiac dysrhythmia	1,851 (6.0%)	1,273 (5.7%)	0.01	894 (5.5%)	914 (5.7%)	-0.01
Valve disorders	1,675 (5.5%)	1,006 (4.5%)	0.05	774 (4.8%)	760 (4.7%)	0.00
Other cardiovascular disease	2,235 (7.3%)	1,554 (7.0%)	0.01	1,085 (6.7%)	1,110 (6.9%)	-0.01
Ischemic stroke	1,425 (4.6%)	851 (3.8%)	0.04	616 (3.8%)	633 (3.9%)	-0.01
TIA	327 (1.1%)	201 (0.9%)	0.02	130 (0.8%)	154 (1.0%)	-0.02
Other cerebrovascular conditions	1,112 (3.6%)	617 (2.8%)	0.05	456 (2.8%)	466 (2.9%)	-0.01
PAD and generalized/unspecified atherosclerosis	2,250 (7.3%)	1,296 (5.8%)	0.06	977 (6.1%)	991 (6.1%)	0.00
Acute Kidney Injury	841 (2.7%)	368 (1.6%)	0.08	288 (1.8%)	289 (1.8%)	0.00
CKD	3,868 (12.6%)	1,333 (6.0%)	0.23	2,024 (12.5%)	2,029 (12.6%)	0.00
CKD Stage 1-2	1,754 (5.7%)	1,087 (4.9%)	0.04	822 (5.1%)	818 (5.1%)	0.00
CKD Stage 3-4	3,868 (12.6%)	1,333 (6.0%)	0.23	1,121 (6.9%)	1,158 (7.2%)	-0.01
CKD unspecified	1,153 (3.8%)	487 (2.2%)	0.09	368 (2.3%)	386 (2.4%)	-0.01
Miscellaneous renal disease	1,899 (6.2%)	790 (3.5%)	0.13	585 (3.6%)	623 (3.9%)	-0.02
Major bleeding	1,367 (4.5%)	972 (4.4%)	0.00	706 (4.4%)	687 (4.3%)	0.00
UTI	2,470 (8.1%)	1,279 (5.7%)	0.09	1,036 (6.4%)	1,009 (6.2%)	0.01
Mycotic infections	2,912 (9.5%)	1,857 (8.3%)	0.04	1,383 (8.6%)	1,365 (8.5%)	0.00
Disorder of Electrolyte	1,561 (5.1%)	834 (3.7%)	0.07	654 (4.1%)	627 (3.9%)	0.01

© 2023 D'Andrea E et al. *JAMA Internal Medicine.*

Disorders of fluid balance	618 (2.0%)	336 (1.5%)	0.04	243 (1.5%)	249 (1.5%)	0.00
Edema	1,855 (6.0%)	1,176 (5.3%)	0.03	838 (5.2%)	868 (5.4%)	-0.01
COPD	2,353 (7.7%)	1,388 (6.2%)	0.06	1,079 (6.7%)	1,052 (6.5%)	0.01
Asthma	1,715 (5.6%)	1,298 (5.8%)	-0.01	878 (5.4%)	920 (5.7%)	-0.01
Obstructive sleep apnea	3,006 (9.8%)	3,095 (13.9%)	-0.13	1,943 (12.0%)	1,921 (11.9%)	0.00
Pneumonia	696 (2.3%)	400 (1.8%)	0.04	293 (1.8%)	297 (1.8%)	0.00
Osteoarthritis	4,052 (13.2%)	2,726 (12.2%)	0.03	2,041 (12.6%)	2,028 (12.6%)	0.00
Osteoporosis without fractures	1,083 (3.5%)	479 (2.1%)	0.08	404 (2.5%)	398 (2.5%)	0.00
Fractures	258 (0.8%)	144 (0.6%)	0.02	103 (0.6%)	111 (0.7%)	-0.01
Falls	736 (2.4%)	441 (2.0%)	0.03	353 (2.2%)	341 (2.1%)	0.01
NASH/NAFLD	1,118 (3.6%)	1,144 (5.1%)	-0.07	732 (4.5%)	735 (4.6%)	0.00
Liver disease	836 (2.7%)	586 (2.6%)	0.01	440 (2.7%)	433 (2.7%)	0.00
Depression	3,207 (10.5%)	2,444 (11.0%)	-0.02	1,691 (10.5%)	1,731 (10.7%)	-0.01
Anxiety and sleep disorders	3,618 (11.8%)	3,074 (13.8%)	-0.06	2,091 (13.0%)	2,116 (13.1%)	0.00
Dementia	684 (2.2%)	270 (1.2%)	0.08	217 (1.3%)	234 (1.4%)	-0.01
Psychosis	213 (0.7%)	126 (0.6%)	0.01	111 (0.7%)	88 (0.5%)	0.03
Delirium	139 (0.5%)	74 (0.3%)	0.03	51 (0.3%)	64 (0.4%)	-0.02
Other medication use, n (%)						
ACEi and ARBs	22,007 (71.7%)	15,897 (71.2%)	0.01	11,460 (71.0%)	11,469 (71.0%)	0.00
Beta blockers	10,166 (33.1%)	6,950 (31.1%)	0.04	5,029 (31.1%)	5,021 (31.1%)	0.00
Calcium channel blockers - DHP & Non-DHP	8,455 (27.6%)	5,413 (24.3%)	0.08	4,040 (25.0%)	4,112 (25.5%)	-0.01
Nitrates and other antianginal agents	1,243 (4.1%)	829 (3.7%)	0.02	581 (3.6%)	583 (3.6%)	0.00
Thiazide and thiazide-like diuretics	3,604 (11.7%)	2,691 (12.1%)	-0.01	1,950 (12.1%)	1,888 (11.7%)	0.01
Loop diuretics	3,234 (10.5%)	1,912 (8.6%)	0.06	1,383 (8.6%)	1,430 (8.9%)	-0.01
MRA (potassium-sparing diuretics)	837 (2.7%)	660 (3.0%)	-0.02	423 (2.6%)	445 (2.8%)	-0.01
Other K-sparing diuretics and antihypertensive medications	2,411 (7.9%)	1,383 (6.2%)	0.07	1,014 (6.3%)	1,061 (6.6%)	-0.01
Antiarrhythmics	294 (1.0%)	221 (1.0%)	0.00	147 (0.9%)	148 (0.9%)	0.00
Anticoagulants (oral)	1,619 (5.3%)	1,154 (5.2%)	0.00	779 (4.8%)	801 (5.0%)	-0.01
Anticoagulants (injectables)	53 (0.2%)	46 (0.2%)	0.00	28 (0.2%)	29 (0.2%)	0.00
Antiplatelet agents	2,703 (8.8%)	1,944 (8.7%)	0.00	1,321 (8.2%)	1,393 (8.6%)	-0.01
Statins	21,830 (71.2%)	16,174 (72.5%)	-0.03	11,554 (71.6%)	11,573 (71.7%)	0.00
PCSK9 inhibitors and other lipid-lowering agents	3,977 (13.0%)	3,185 (14.3%)	-0.04	2,151 (13.3%)	2,120 (13.1%)	0.01
COPD & asthma medications	3,934 (12.8%)	2,834 (12.7%)	0.00	2,000 (12.4%)	2,019 (12.5%)	0.00
Corticosteroids (oral)	2,963 (9.7%)	2,214 (9.9%)	-0.01	1,555 (9.6%)	1,542 (9.6%)	0.00
Antiosteoporosis agents	1,020 (3.3%)	393 (1.8%)	0.10	319 (2.0%)	354 (2.2%)	-0.01
NSAIDs	5,579 (18.2%)	4,343 (19.5%)	-0.03	3,098 (19.2%)	3,086 (19.1%)	0.00
Opioids	6,290 (20.5%)	4,258 (19.1%)	0.04	3,102 (19.2%)	3,137 (19.4%)	-0.01
Gabapentinoids	4,125 (13.4%)	3,025 (13.6%)	-0.01	2,166 (13.4%)	2,149 (13.3%)	0.00
UTI antibiotics	3,779 (12.3%)	2,241 (10.0%)	0.07	1,670 (10.3%)	1,673 (10.4%)	0.00
Antidepressants	6,502 (21.2%)	5,014 (22.5%)	-0.03	3,471 (21.5%)	3,523 (21.8%)	-0.01
Anxiolytics/hypnotics	1,616 (5.3%)	1,296 (5.8%)	-0.02	898 (5.6%)	880 (5.5%)	0.00
Benzodiazepines	2,827 (9.2%)	1,883 (8.4%)	0.03	1,391 (8.6%)	1,417 (8.8%)	-0.01
Antipsychotics	731 (2.4%)	521 (2.3%)	0.01	385 (2.4%)	372 (2.3%)	0.01
Antiparkinsonian medications	573 (1.9%)	429 (1.9%)	0.00	276 (1.7%)	295 (1.8%)	-0.01
Dementia medications	575 (1.9%)	194 (0.9%)	0.09	197 (1.2%)	165 (1.0%)	0.02
Measures of health care use						
Internist; n (%)	28,619 (93.3%)	20,190 (90.5%)	0.10	14,873 (92.1%)	14,845 (91.9%)	0.01
Internist (-30 days to CED); n (%)	23,960 (78.1%)	16,168 (72.5%)	0.13	12,280 (76.1%)	12,278 (76.0%)	0.00

© 2023 D'Andrea E et al. *JAMA Internal Medicine.*

Internist (-180 days to -31 days before CED); n (%)	24,673 (80.4%)	17,246 (77.3%)	0.08	12,702 (78.7%)	12,609 (78.1%)	0.01
Internist (Number of visits)						
...mean (sd)	11.88 (16.13)	10.02 (14.52)	0.12	10.58 (13.95)	10.63 (14.99)	0.00
Endocrinologist; n (%)	2,609 (8.5%)	3,877 (17.4%)	-0.27	1,731 (10.7%)	1,812 (11.2%)	-0.02
Endocrinologist (-30 days to CED); n (%)	1,826 (6.0%)	3,035 (13.6%)	-0.26	1,302 (8.1%)	1,303 (8.1%)	0.00
Endocrinologist (-180 days to -31 days before CED); n (%)	1,735 (5.7%)	2,739 (12.3%)	-0.23	1,125 (7.0%)	1,272 (7.9%)	-0.03
Endocrinologist (Number of visits)						
...mean (sd)	0.32 (1.55)	0.66 (2.17)	-0.18	0.40 (1.71)	0.40 (1.64)	0.00
Cardiologist; n (%)	6,726 (21.9%)	4,706 (21.1%)	0.02	3,278 (20.3%)	3,348 (20.7%)	-0.01
Cardiologist (-30 days to CED); n (%)	2,492 (8.1%)	1,771 (7.9%)	0.01	1,222 (7.6%)	1,230 (7.6%)	0.00
Cardiologist (-180 days to -31 days before CED); n (%)	5,619 (18.3%)	3,989 (17.9%)	0.01	2,731 (16.9%)	2,814 (17.4%)	-0.01
Cardiologist (Number of visits)						
...mean (sd)	1.10 (3.51)	1.07 (3.48)	0.01	1.02 (3.42)	1.02 (3.38)	0.00
Electrocardiogram; n (%)	8,654 (28.2%)	5,786 (25.9%)	0.05	4,175 (25.9%)	4,238 (26.2%)	-0.01
Electrocardiogram (Number of tests)						
...mean (sd)	0.53 (1.29)	0.49 (1.25)	0.03	0.48 (1.19)	0.49 (1.26)	-0.01
ECG & Other cardiac imaging; n (%)	3,366 (11.0%)	2,317 (10.4%)	0.02	1,604 (9.9%)	1,653 (10.2%)	-0.01
ECG & Other cardiac imaging (Number of tests)						
...mean (sd)	0.38 (2.65)	0.38 (2.54)	0.00	0.36 (2.51)	0.37 (2.49)	0.00
Cardiovascular stress test; n (%)	1,404 (4.6%)	1,064 (4.8%)	-0.01	678 (4.2%)	760 (4.7%)	-0.02
HbA1c test order (Number of tests)						
...mean (sd)	1.60 (0.84)	1.63 (0.81)	-0.04	1.60 (0.81)	1.60 (0.81)	0.00
Glucose test and monitoring; n (%)	6,721 (21.9%)	4,906 (22.0%)	0.00	3,448 (21.4%)	3,319 (20.6%)	0.02
Glucose test and monitoring (Number of tests)						
...mean (sd)	0.47 (1.29)	0.47 (1.26)	0.00	0.44 (1.14)	0.44 (1.25)	0.00
Microalbuminuria/proteinuria test order; n (%)	16,781 (54.7%)	12,635 (56.6%)	-0.04	8,939 (55.4%)	8,954 (55.5%)	0.00
Microalbuminuria/proteinuria test order (Number of tests)						
...mean (sd)	0.71 (0.80)	0.72 (0.76)	-0.01	0.70 (0.77)	0.70 (0.75)	0.00
Metabolic or renal/creatinine panel test order; n (%)	26,802 (87.4%)	19,125 (85.7%)	0.05	13,955 (86.4%)	13,784 (85.4%)	0.03
Metabolic or renal/creatinine panel test order (Number of tests)						
...mean (sd)	1.70 (1.41)	1.63 (1.35)	0.05	1.61 (1.29)	1.62 (1.37)	-0.01
Lipid test order; n (%)	26,104 (85.1%)	19,269 (86.4%)	-0.04	13,895 (86.1%)	13,841 (85.7%)	0.01
Lipid test order (Number of tests)						
...mean (sd)	1.31 (0.93)	1.32 (0.91)	-0.01	1.31 (0.90)	1.30 (0.88)	0.01
Uric acid test order; n (%)	2,869 (9.4%)	1,775 (8.0%)	0.05	1,291 (8.0%)	1,351 (8.4%)	-0.01
Vitamin D test order; n (%)	8,297 (27.0%)	5,548 (24.9%)	0.05	4,110 (25.5%)	4,035 (25.0%)	0.01
PTH test order; n (%)	1,795 (5.9%)	923 (4.1%)	0.08	777 (4.8%)	689 (4.3%)	0.02
Hospitalization event (Number of)						
...mean (sd)	0.07 (0.31)	0.05 (0.27)	0.07	0.06 (0.29)	0.05 (0.28)	0.04
LOS (-180 days to -31 days before CED)						
...mean (sd)	0.20 (1.66)	0.17 (1.39)	0.02	0.18 (1.44)	0.17 (1.43)	0.01
LOS (-30 days to CED)						
...mean (sd)	0.08 (0.87)	0.04 (0.61)	0.05	0.05 (0.66)	0.05 (0.61)	0.00
ED event (Number of)						
...mean (sd)	0.21 (0.87)	0.13 (0.58)	0.11	0.14 (0.58)	0.14 (0.62)	0.00

© 2023 D’Andrea E et al. *JAMA Internal Medicine.*

Distinct medications ALL						
...mean (sd)	9.90 (4.81)	10.13 (5.00)	-0.05	9.77 (4.76)	9.80 (4.89)	-0.01
Distinct medications all BRAND						
...mean (sd)	2.04 (1.33)	2.32 (1.49)	-0.20	2.06 (1.33)	2.08 (1.37)	-0.01
Cost pharmacy OOP ($)						
...mean (sd)	367.48 (382.64)	447.51 (488.59)	-0.18	387.44 (422.39)	391.10 (399.93)	-0.01
Flu vaccine; n (%)	6,249 (20.4%)	4,537 (20.3%)	0.00	3,192 (19.8%)	3,282 (20.3%)	-0.01
Pneumococcal vaccine; n (%)	1,837 (6.0%)	1,235 (5.5%)	0.02	875 (5.4%)	935 (5.8%)	-0.02
Breast mammography or MRI; n (%)	3,777 (12.3%)	2,660 (11.9%)	0.01	1,995 (12.4%)	1,945 (12.0%)	0.01
Prostate DRE or PSA; n (%)	5,679 (18.5%)	4,391 (19.7%)	-0.03	3,173 (19.7%)	3,098 (19.2%)	0.01
Colonoscopy; n (%)	1,244 (4.1%)	976 (4.4%)	-0.01	684 (4.2%)	694 (4.3%)	0.00
Pap smear; n (%)	1,095 (3.6%)	922 (4.1%)	-0.03	613 (3.8%)	628 (3.9%)	-0.01
Bone mineral density; n (%)	1,141 (3.7%)	625 (2.8%)	0.05	526 (3.3%)	487 (3.0%)	0.02
Laboratory results						
Lab value HbA1c (%)						
...mean (sd)	8.15 (0.57)	8.21 (0.59)	-0.10	8.18 (0.58)	8.18 (0.58)	0.00
...median [IQR]	8.10 [7.70, 8.50]	8.15 [7.80, 8.60]	-	8.10 [7.75, 8.55]	8.10 [7.75, 8.55]	-
eGFR (w/o race)						
...mean (sd)	73.33 (24.32)	78.65 (23.51)	-0.22	77.80 (23.51)	77.44 (23.26)	0.02
...median [IQR]	76.23 [56.03, 92.00]	82.34 [64.23, 95.90]	-	81.90 [62.83, 95.09]	80.51 [62.78, 94.69]	-
Lab result number - Creatinine						
...mean (sd)	1.09 (1.03)	1.07 (1.12)	0.02	1.06 (1.16)	1.06 (1.09)	0.00
...median [IQR]	0.92 [0.77, 1.16]	0.90 [0.76, 1.09]	-	0.90 [0.75, 1.10]	0.91 [0.76, 1.10]	-
Lab result number - UACR						
...mean (sd)	134.23 (550.59)	106.06 (435.57)	0.06	123.14 (517.09)	101.81 (399.81)	0.05
...Missing; n (%)	18,535 (60.4%)	13,099 (58.7%)	0.03	9,551 (59.2%)	9,747 (60.4%)	-0.02
Lab result number - Total cholesterol						
...mean (sd)	174.12 (47.94)	173.72 (51.51)	0.01	173.41 (48.59)	174.65 (51.29)	-0.02
...Missing; n (%)	4,378 (14.3%)	3,236 (14.5%)	-0.01	2,177 (13.5%)	2,330 (14.4%)	-0.03
Lab result number - LDL cholesterol						
...mean (sd)	94.26 (40.99)	93.96 (42.92)	0.01	93.97 (41.25)	94.46 (42.96)	-0.01
...Missing; n (%)	4,914 (16.0%)	3,752 (16.8%)	-0.02	2,526 (15.6%)	2,671 (16.5%)	-0.02
Lab result number - HDL cholesterol						
...mean (sd)	47.82 (18.80)	47.45 (20.75)	0.02	47.10 (19.41)	47.77 (20.16)	-0.03
...Missing; n (%)	4,685 (15.3%)	3,537 (15.9%)	-0.02	2,365 (14.6%)	2,555 (15.8%)	-0.03
Lab result number - Triglycerides						
...mean (sd)	189.85 (136.03)	205.23 (160.48)	-0.10	196.16 (146.87)	201.17 (155.41)	-0.03
...Missing; n (%)	4,412 (14.4%)	3,180 (14.3%)	0.00	2,168 (13.4%)	2,300 (14.2%)	-0.02
Lab result value - Glucose						
...mean (sd)	179.73 (84.21)	186.89 (103.65)	-0.08	182.98 (90.96)	184.65 (96.67)	-0.02
...Missing; n (%)	977 (3.2%)	612 (2.7%)	0.03	472 (2.9%)	435 (2.7%)	0.01

Abbreviations. St. Diff., standardized difference; SD, standard deviation; GLP-1 RA, Glucagon-like peptide-1 receptor agonists; MI, myocardial infarction; TIA, transient ischemic attack; peripheral artery disease; NASH/NAFLD, Nonalcoholic steatohepatitis/Nonalcoholic fatty liver disease; COPD, chronic obstructive pulmonary disease; CKD, chronic kidney disease; ARBs, Angiotensin II Receptor Blockers; ACEis, Angiotensin Converting Enzyme inhibitors; MRA, mineralocorticoid receptor antagonist; PCSK9, Proprotein convertase subtilisin/kexin type 9; NSAIDs, nonsteroidal anti-inflammatory drugs; MRI, magnetic resonance imaging; DRE, digital rectal examination; PSA, prostate-specific antigen; ED, emergency department, LOS, length of stay; ECG, electrocardiogram; eGFR, estimated glomerular filtration rate; uACR, urine albumin-creatinine ratio; LDL, low-density lipoprotein; HDL, high-density lipoprotein.

© 2023 D’Andrea E et al. *JAMA Internal Medicine.*

eTable 6. Baseline characteristics of patients with HbA_{1c} > 9% initiating SGLT2 inhibitor vs. DPP4 inhibitor therapy before and after 1:1 PS-matching

Baseline characteristics	DPP-4 inhibitor	SGLT2 inhibitor	St. Diff.	DPP-4 inhibitor	SGLT2 inhibitor	St. Diff.
Number of patients	25634	21895	-	15,466	15,466	-
Demographics						
Age						
...mean (sd)	59.96 (12.98)	56.68 (11.86)	0.26	57.74 (12.13)	57.64 (12.17)	0.01
...median [IQR]	61 [51, 69]	57 [48, 66]	-	58 [49, 67]	58 [49, 67]	-
Age squared						
...mean (sd)	3,764.17 (1,534.32)	3,353.03 (1,334.61)	0.29	3,481.33 (1,384.16)	3,470.31 (1,383.31)	0.01
...median [IQR]	3,721 [2,601, 4,761]	3,249 [2,304, 4,356]	-	3,364 [2,401, 4,489]	3,364 [2,401, 4,489]	-
Age Categories						
...18 - 39; n (%)	1,707 (6.7%)	1,746 (8.0%)	-0.05	1,170 (7.6%)	1,183 (7.6%)	0.00
...40 - 44; n (%)	1,643 (6.4%)	1,727 (7.9%)	-0.06	1,160 (7.5%)	1,124 (7.3%)	0.01
...45 - 49; n (%)	2,382 (9.3%)	2,630 (12.0%)	-0.09	1,678 (10.8%)	1,683 (10.9%)	0.00
...50 - 54; n (%)	3,032 (11.8%)	3,209 (14.7%)	-0.09	2,101 (13.6%)	2,087 (13.5%)	0.00
...55 - 59; n (%)	3,288 (12.8%)	3,386 (15.5%)	-0.08	2,234 (14.4%)	2,292 (14.8%)	-0.01
...60 - 64; n (%)	2,866 (11.2%)	2,822 (12.9%)	-0.05	1,808 (11.7%)	2,011 (13.0%)	-0.04
...65 - 69; n (%)	4,404 (17.2%)	3,266 (14.9%)	0.06	2,646 (17.1%)	2,449 (15.8%)	0.04
...70 - 74; n (%)	3,090 (12.1%)	1,859 (8.5%)	0.12	1,612 (10.4%)	1,521 (9.8%)	0.02
...75 - 79; n (%)	1,713 (6.7%)	841 (3.8%)	0.13	687 (4.4%)	728 (4.7%)	-0.01
...80 - 84; n (%)	1,001 (3.9%)	308 (1.4%)	0.16	281 (1.8%)	290 (1.9%)	-0.01
...>= 85; n (%)	508 (2.0%)	101 (0.5%)	0.14	89 (0.6%)	98 (0.6%)	0.00
Gender						
…Male; n (%)	14,047 (54.8%)	12,782 (58.4%)	-0.07	8,859 (57.3%)	8,846 (57.2%)	0.00
…Female; n (%)	11,587 (45.2%)	9,113 (41.6%)	0.07	6,607 (42.7%)	6,620 (42.8%)	0.00
Geographic region						
...Northeast; n (%)	2,491 (9.7%)	1,591 (7.3%)	0.09	1,194 (7.7%)	1,206 (7.8%)	0.00
...South; n (%)	15,000 (58.5%)	13,524 (61.8%)	-0.07	9,378 (60.6%)	9,421 (60.9%)	-0.01
...Midwest; n (%)	1,995 (7.8%)	1,899 (8.7%)	-0.03	1,307 (8.5%)	1,270 (8.2%)	0.01
...West; n (%)	6,148 (24.0%)	4,881 (22.3%)	0.04	3,587 (23.2%)	3,569 (23.1%)	0.00
Race categories						
...White; n (%)	11,060 (43.1%)	10,712 (48.9%)	-0.12	7,141 (46.2%)	7,138 (46.2%)	0.00
...Black; n (%)	3,859 (15.1%)	2,946 (13.5%)	0.05	2,184 (14.1%)	2,193 (14.2%)	0.00
...Asian; n (%)	1,545 (6.0%)	991 (4.5%)	0.07	785 (5.1%)	795 (5.1%)	0.00
...Hispanic; n (%)	7,749 (30.2%)	5,932 (27.1%)	0.07	4,439 (28.7%)	4,418 (28.6%)	0.00
...Other or unknown; n (%)	1,421 (5.5%)	1,314 (6.0%)	-0.02	917 (5.9%)	922 (6.0%)	0.00
Quarter/Year of Cohort Entry Date						
...Q2 2013; n (%)	799 (3.1%)	44 (0.2%)	0.23	31 (0.2%)	44 (0.3%)	-0.02
...Q3 2013; n (%)	825 (3.2%)	141 (0.6%)	0.19	111 (0.7%)	135 (0.9%)	-0.02
...Q4 2013; n (%)	779 (3.0%)	182 (0.8%)	0.16	140 (0.9%)	174 (1.1%)	-0.02
...Q1 2014; n (%)	803 (3.1%)	189 (0.9%)	0.16	165 (1.1%)	179 (1.2%)	-0.01
...Q2 2014; n (%)	736 (2.9%)	273 (1.2%)	0.12	244 (1.6%)	240 (1.6%)	0.00
...Q3 2014; n (%)	814 (3.2%)	421 (1.9%)	0.08	355 (2.3%)	357 (2.3%)	0.00
...Q4 2014; n (%)	810 (3.2%)	488 (2.2%)	0.06	411 (2.7%)	432 (2.8%)	-0.01
...Q1 2015; n (%)	727 (2.8%)	567 (2.6%)	0.01	473 (3.1%)	465 (3.0%)	0.01
...Q2 2015; n (%)	769 (3.0%)	580 (2.6%)	0.02	485 (3.1%)	477 (3.1%)	0.00
...Q3 2015; n (%)	787 (3.1%)	483 (2.2%)	0.06	426 (2.8%)	410 (2.7%)	0.01
...Q4 2015; n (%)	665 (2.6%)	458 (2.1%)	0.03	417 (2.7%)	403 (2.6%)	0.01
...Q1 2016; n (%)	845 (3.3%)	550 (2.5%)	0.05	475 (3.1%)	462 (3.0%)	0.01

© 2023 D'Andrea E et al. *JAMA Internal Medicine.*

...Q2 2016; n (%)	831 (3.2%)	545 (2.5%)	0.04	490 (3.2%)	474 (3.1%)	0.01
...Q3 2016; n (%)	907 (3.5%)	615 (2.8%)	0.04	548 (3.5%)	536 (3.5%)	0.00
...Q4 2016; n (%)	836 (3.3%)	604 (2.8%)	0.03	495 (3.2%)	490 (3.2%)	0.00
...Q1 2017; n (%)	907 (3.5%)	659 (3.0%)	0.03	565 (3.7%)	551 (3.6%)	0.01
...Q2 2017; n (%)	934 (3.6%)	693 (3.2%)	0.02	598 (3.9%)	581 (3.8%)	0.01
...Q3 2017; n (%)	968 (3.8%)	737 (3.4%)	0.02	597 (3.9%)	585 (3.8%)	0.01
...Q4 2017; n (%)	755 (2.9%)	613 (2.8%)	0.01	492 (3.2%)	481 (3.1%)	0.01
...Q1 2018; n (%)	838 (3.3%)	642 (2.9%)	0.02	510 (3.3%)	520 (3.4%)	-0.01
...Q2 2018; n (%)	884 (3.4%)	685 (3.1%)	0.02	546 (3.5%)	538 (3.5%)	0.00
...Q3 2018; n (%)	787 (3.1%)	755 (3.4%)	-0.02	573 (3.7%)	554 (3.6%)	0.01
...Q4 2018; n (%)	670 (2.6%)	657 (3.0%)	-0.02	497 (3.2%)	467 (3.0%)	0.01
...Q1 2019; n (%)	792 (3.1%)	831 (3.8%)	-0.04	589 (3.8%)	590 (3.8%)	0.00
...Q2 2019; n (%)	821 (3.2%)	978 (4.5%)	-0.07	645 (4.2%)	655 (4.2%)	0.00
...Q3 2019; n (%)	693 (2.7%)	909 (4.2%)	-0.08	544 (3.5%)	572 (3.7%)	-0.01
...Q4 2019; n (%)	595 (2.3%)	847 (3.9%)	-0.09	512 (3.3%)	515 (3.3%)	0.00
...Q1 2020; n (%)	755 (2.9%)	1,151 (5.3%)	-0.12	650 (4.2%)	646 (4.2%)	0.00
...Q2 2020; n (%)	580 (2.3%)	761 (3.5%)	-0.07	476 (3.1%)	489 (3.2%)	-0.01
...Q3 2020; n (%)	746 (2.9%)	1,045 (4.8%)	-0.10	617 (4.0%)	630 (4.1%)	-0.01
...Q4 2020; n (%)	652 (2.5%)	1,124 (5.1%)	-0.14	567 (3.7%)	588 (3.8%)	-0.01
...Q1 2021; n (%)	650 (2.5%)	1,237 (5.6%)	-0.16	588 (3.8%)	598 (3.9%)	-0.01
...Q2 2021; n (%)	674 (2.6%)	1,431 (6.5%)	-0.19	634 (4.1%)	628 (4.1%)	0.00
Burden of comorbidities						
Com. comorbidity score, mean (sd)	1.00 (1.87)	0.95 (1.71)	0.03	0.91 (1.70)	0.92 (1.74)	-0.01
Frailty Score, mean (sd)	0.14 (0.04)	0.14 (0.04)	0.00	0.14 (0.04)	0.14 (0.04)	0.00
Frailty Score squared, mean (sd)	0.02 (0.01)	0.02 (0.01)	0.00	0.02 (0.01)	0.02 (0.01)	0.00
Frailty score; n (%)			-			
...< 0.15	17,370 (67.8%)	15,252 (69.7%)	-0.04	10,878 (70.3%)	10,897 (70.5%)	0.00
...0.15 - 0.25	7,728 (30.1%)	6,235 (28.5%)	0.04	4,349 (28.1%)	4,282 (27.7%)	0.01
...>= 0.25	536 (2.1%)	408 (1.9%)	0.01	239 (1.5%)	287 (1.9%)	-0.03
Lifestyle factors, n (%)						
Overweight	1,989 (7.8%)	1,802 (8.2%)	-0.01	1,283 (8.3%)	1,267 (8.2%)	0.00
Obesity	6,932 (27.0%)	7,952 (36.3%)	-0.20	4,922 (31.8%)	4,941 (31.9%)	0.00
Smoking	3,181 (12.4%)	2,915 (13.3%)	-0.03	1,991 (12.9%)	1,985 (12.8%)	0.00
Diabetes-related conditions, n (%)						
Diabetic nephropathy	4,019 (15.7%)	2,830 (12.9%)	0.08	2,051 (13.3%)	2,042 (13.2%)	0.00
Diabetic retinopathy	1,986 (7.7%)	1,718 (7.8%)	0.00	1,139 (7.4%)	1,134 (7.3%)	0.00
Diabetes with other ophthalmic manifestations	1,065 (4.2%)	768 (3.5%)	0.04	545 (3.5%)	557 (3.6%)	-0.01
Diabetic neuropathy	4,787 (18.7%)	4,459 (20.4%)	-0.04	2,934 (19.0%)	2,945 (19.0%)	0.00
Diabetes with peripheral circulatory disorders	365 (1.4%)	130 (0.6%)	0.08	120 (0.8%)	116 (0.8%)	0.00
Diabetic foot	452 (1.8%)	387 (1.8%)	0.00	261 (1.7%)	263 (1.7%)	0.00
Infection of lower extremities	600 (2.3%)	528 (2.4%)	-0.01	346 (2.2%)	358 (2.3%)	-0.01
Lower-limb amputations	169 (0.7%)	145 (0.7%)	0.00	94 (0.6%)	90 (0.6%)	0.00
Erectile dysfunction	811 (3.2%)	895 (4.1%)	-0.05	540 (3.5%)	556 (3.6%)	-0.01
Hypoglycemia	3,197 (12.5%)	3,137 (14.3%)	-0.05	2,054 (13.3%)	2,060 (13.3%)	0.00
Hyperglycemia	10,196 (39.8%)	11,238 (51.3%)	-0.23	7,272 (47.0%)	7,168 (46.3%)	0.01
Diabetic ketoacidosis	78 (0.3%)	66 (0.3%)	0.00	49 (0.3%)	43 (0.3%)	0.00
Hyperosmolar hyperglycemic nonketotic syndrome	162 (0.6%)	132 (0.6%)	0.00	92 (0.6%)	96 (0.6%)	0.00
Diabetes with other complications	1,480 (5.8%)	1,480 (6.8%)	-0.04	954 (6.2%)	943 (6.1%)	0.00
Diabetes mellitus without mention of complications	19,732 (77.0%)	15,865 (72.5%)	0.10	11,367 (73.5%)	11,366 (73.5%)	0.00

© 2023 D'Andrea E et al. *JAMA Internal Medicine.*

Diabetes treatment						
Number of anti-DM medications on index date						
...mean (sd)	1.32 (0.78)	1.42 (0.91)	-0.12	1.33 (0.80)	1.32 (0.85)	0.01
...median [IQR]	1.00 [1.00, 2.00]	1.00 [1.00, 2.00]	-	1.00 [1.00, 2.00]	1.00 [1.00, 2.00]	-
No use of anti-DM medications in prior 180 days; n (%)	4,630 (18.1%)	2,584 (11.8%)	0.18	2,290 (14.8%)	2,308 (14.9%)	0.00
Metformin (any use); n (%)	19,777 (77.2%)	16,773 (76.6%)	0.01	12,013 (77.7%)	11,957 (77.3%)	0.01
Metformin (concurrent use); n (%)	17,562 (68.5%)	14,364 (65.6%)	0.06	10,584 (68.4%)	10,360 (67.0%)	0.03
Sulfonylureas - 2nd gen. (any use); n (%)	11,041 (43.1%)	7,813 (35.7%)	0.15	6,025 (39.0%)	6,062 (39.2%)	0.00
Sulfonylureas - 2nd gen. (concurrent use); n (%)	9,159 (35.7%)	6,263 (28.6%)	0.15	4,937 (31.9%)	4,937 (31.9%)	0.00
Thiazolidinediones (any use); n (%)	1,883 (7.3%)	1,773 (8.1%)	-0.03	1,191 (7.7%)	1,196 (7.7%)	0.00
Thiazolidinediones (concurrent use); n (%)	1,579 (6.2%)	1,404 (6.4%)	-0.01	994 (6.4%)	962 (6.2%)	0.01
GLP-1RA (any use); n (%)	1,247 (4.9%)	4,544 (20.8%)	-0.49	1,190 (7.7%)	1,252 (8.1%)	-0.01
GLP-1RA (concurrent use); n (%)	806 (3.1%)	3,510 (16.0%)	-0.45	772 (5.0%)	976 (6.3%)	-0.06
Insulins (any use); n (%)	5,683 (22.2%)	6,957 (31.8%)	-0.22	3,957 (25.6%)	3,961 (25.6%)	0.00
Insulins (concurrent use); n (%)	4,476 (17.5%)	5,433 (24.8%)	-0.18	3,086 (20.0%)	3,109 (20.1%)	0.00
Long term use of insulin; n (%)	2,527 (9.9%)	3,448 (15.7%)	-0.17	1,850 (12.0%)	1,836 (11.9%)	0.00
Miscellaneous anti-DM medications; n (%)	373 (1.5%)	251 (1.1%)	0.04	192 (1.2%)	188 (1.2%)	0.00
Other comorbidities at baseline, n (%)						
Hypertension	17,523 (68.4%)	15,251 (69.7%)	-0.03	10,487 (67.8%)	10,525 (68.1%)	-0.01
Hyperlipidemia	18,369 (71.7%)	16,374 (74.8%)	-0.07	11,287 (73.0%)	11,251 (72.7%)	0.01
Acute MI	192 (0.7%)	216 (1.0%)	-0.03	116 (0.8%)	118 (0.8%)	0.00
MI sequelae/old MI	494 (1.9%)	538 (2.5%)	-0.04	302 (2.0%)	321 (2.1%)	-0.01
Stable angina	690 (2.7%)	670 (3.1%)	-0.02	415 (2.7%)	415 (2.7%)	0.00
ACS unstable angina	227 (0.9%)	259 (1.2%)	-0.03	140 (0.9%)	139 (0.9%)	0.00
Coronary atherosclerosis	2,766 (10.8%)	2,530 (11.6%)	-0.03	1,613 (10.4%)	1,647 (10.6%)	-0.01
Coronary procedure	157 (0.6%)	184 (0.8%)	-0.02	104 (0.7%)	104 (0.7%)	0.00
History of coronary procedure	743 (2.9%)	809 (3.7%)	-0.04	463 (3.0%)	471 (3.0%)	0.00
Congestive heart failure	1,415 (5.5%)	1,162 (5.3%)	0.01	731 (4.7%)	767 (5.0%)	-0.01
Cardiomyopathy	494 (1.9%)	490 (2.2%)	-0.02	286 (1.8%)	299 (1.9%)	-0.01
Atrial fibrillation	996 (3.9%)	761 (3.5%)	0.02	509 (3.3%)	523 (3.4%)	-0.01
Cardiac conduction disorders	393 (1.5%)	323 (1.5%)	0.00	217 (1.4%)	221 (1.4%)	0.00
Other cardiac dysrhythmia	1,201 (4.7%)	1,063 (4.9%)	-0.01	683 (4.4%)	685 (4.4%)	0.00
Valve disorders	964 (3.8%)	710 (3.2%)	0.03	491 (3.2%)	474 (3.1%)	0.01
Other cardiovascular disease	1,425 (5.6%)	1,311 (6.0%)	-0.02	837 (5.4%)	870 (5.6%)	-0.01
Ischemic stroke	904 (3.5%)	676 (3.1%)	0.02	467 (3.0%)	470 (3.0%)	0.00
TIA	226 (0.9%)	158 (0.7%)	0.02	118 (0.8%)	119 (0.8%)	0.00
Other cerebrovascular conditions	828 (3.2%)	581 (2.7%)	0.03	411 (2.7%)	412 (2.7%)	0.00
PAD and generalized/unspecified atherosclerosis	1,539 (6.0%)	1,226 (5.6%)	0.02	849 (5.5%)	839 (5.4%)	0.00
Acute Kidney Injury	596 (2.3%)	328 (1.5%)	0.06	250 (1.6%)	254 (1.6%)	0.00
CKD	2,243 (8.8%)	941 (4.3%)	0.18	1,548 (10.0%)	1,570 (10.2%)	-0.01
CKD Stage 1-2	1,296 (5.1%)	930 (4.2%)	0.04	719 (4.6%)	701 (4.5%)	0.00
CKD Stage 3-4	2,243 (8.8%)	941 (4.3%)	0.18	777 (5.0%)	817 (5.3%)	-0.01
CKD unspecified	692 (2.7%)	318 (1.5%)	0.08	240 (1.6%)	256 (1.7%)	-0.01
Miscellaneous renal disease	1,144 (4.5%)	646 (3.0%)	0.08	488 (3.2%)	509 (3.3%)	-0.01
Major bleeding	952 (3.7%)	861 (3.9%)	-0.01	602 (3.9%)	601 (3.9%)	0.00
UTI	1,972 (7.7%)	1,231 (5.6%)	0.08	941 (6.1%)	945 (6.1%)	0.00
Mycotic infections	2,440 (9.5%)	1,986 (9.1%)	0.01	1,421 (9.2%)	1,418 (9.2%)	0.00
Disorder of Electrolyte	1,090 (4.3%)	752 (3.4%)	0.05	547 (3.5%)	507 (3.3%)	0.01

© 2023 D'Andrea E et al. *JAMA Internal Medicine.*

Disorders of fluid balance	575 (2.2%)	391 (1.8%)	0.03	301 (1.9%)	262 (1.7%)	0.02
Edema	1,139 (4.4%)	956 (4.4%)	0.00	628 (4.1%)	634 (4.1%)	0.00
COPD	1,568 (6.1%)	1,245 (5.7%)	0.02	860 (5.6%)	875 (5.7%)	0.00
Asthma	1,208 (4.7%)	1,113 (5.1%)	-0.02	743 (4.8%)	752 (4.9%)	0.00
Obstructive sleep apnea	2,077 (8.1%)	2,423 (11.1%)	-0.10	1,448 (9.4%)	1,452 (9.4%)	0.00
Pneumonia	480 (1.9%)	424 (1.9%)	0.00	265 (1.7%)	273 (1.8%)	-0.01
Osteoarthritis	2,453 (9.6%)	2,156 (9.8%)	-0.01	1,447 (9.4%)	1,468 (9.5%)	0.00
Osteoporosis without fractures	562 (2.2%)	309 (1.4%)	0.06	250 (1.6%)	244 (1.6%)	0.00
Fractures	191 (0.7%)	144 (0.7%)	0.00	102 (0.7%)	108 (0.7%)	0.00
Falls	540 (2.1%)	437 (2.0%)	0.01	296 (1.9%)	306 (2.0%)	-0.01
NASH/NAFLD	903 (3.5%)	999 (4.6%)	-0.06	621 (4.0%)	626 (4.0%)	0.00
Liver disease	674 (2.6%)	511 (2.3%)	0.02	379 (2.5%)	369 (2.4%)	0.01
Depression	2,548 (9.9%)	2,498 (11.4%)	-0.05	1,605 (10.4%)	1,636 (10.6%)	-0.01
Anxiety and sleep disorders	2,824 (11.0%)	2,839 (13.0%)	-0.06	1,853 (12.0%)	1,866 (12.1%)	0.00
Dementia	523 (2.0%)	227 (1.0%)	0.08	199 (1.3%)	185 (1.2%)	0.01
Psychosis	203 (0.8%)	140 (0.6%)	0.02	126 (0.8%)	100 (0.6%)	0.02
Delirium	93 (0.4%)	62 (0.3%)	0.02	42 (0.3%)	43 (0.3%)	0.00
Other medication use, n (%)						
ACEi and ARBs	16,756 (65.4%)	14,581 (66.6%)	-0.03	10,125 (65.5%)	10,151 (65.6%)	0.00
Beta blockers	6,665 (26.0%)	5,730 (26.2%)	0.00	3,852 (24.9%)	3,886 (25.1%)	0.00
Calcium channel blockers - DHP & Non-DHP	5,800 (22.6%)	4,545 (20.8%)	0.04	3,211 (20.8%)	3,262 (21.1%)	-0.01
Nitrates and other antianginal agents	801 (3.1%)	732 (3.3%)	-0.01	443 (2.9%)	488 (3.2%)	-0.02
Thiazide and thiazide-like diuretics	2,552 (10.0%)	2,220 (10.1%)	0.00	1,524 (9.9%)	1,504 (9.7%)	0.01
Loop diuretics	2,090 (8.2%)	1,699 (7.8%)	0.01	1,090 (7.0%)	1,133 (7.3%)	-0.01
MRA (potassium-sparing diuretics)	569 (2.2%)	603 (2.8%)	-0.04	373 (2.4%)	371 (2.4%)	0.00
Other K-sparing diuretics and antihypertensive medications	1,560 (6.1%)	1,136 (5.2%)	0.04	825 (5.3%)	832 (5.4%)	0.00
Antiarrhythmics	181 (0.7%)	130 (0.6%)	0.01	93 (0.6%)	88 (0.6%)	0.00
Anticoagulants (oral)	980 (3.8%)	850 (3.9%)	-0.01	539 (3.5%)	565 (3.7%)	-0.01
Anticoagulants (injectables)	26 (0.1%)	19 (0.1%)	0.00	18 (0.1%)	9 (0.1%)	0.00
Antiplatelet agents	1,979 (7.7%)	1,742 (8.0%)	-0.01	1,162 (7.5%)	1,173 (7.6%)	0.00
Statins	16,126 (62.9%)	14,360 (65.6%)	-0.06	9,907 (64.1%)	9,877 (63.9%)	0.00
PCSK9 inhibitors and other lipid-lowering agents	2,872 (11.2%)	2,776 (12.7%)	-0.05	1,758 (11.4%)	1,784 (11.5%)	0.00
COPD & asthma medications	2,745 (10.7%)	2,356 (10.8%)	0.00	1,607 (10.4%)	1,652 (10.7%)	-0.01
Corticosteroids (oral)	2,068 (8.1%)	1,960 (9.0%)	-0.03	1,311 (8.5%)	1,312 (8.5%)	0.00
Antiosteoporosis agents	497 (1.9%)	261 (1.2%)	0.06	222 (1.4%)	224 (1.4%)	0.00
NSAIDs	4,473 (17.4%)	4,238 (19.4%)	-0.05	2,913 (18.8%)	2,909 (18.8%)	0.00
Opioids	4,986 (19.5%)	4,210 (19.2%)	0.01	2,950 (19.1%)	2,967 (19.2%)	0.00
Gabapentinoids	3,281 (12.8%)	3,036 (13.9%)	-0.03	2,012 (13.0%)	2,036 (13.2%)	-0.01
UTI antibiotics	3,107 (12.1%)	2,279 (10.4%)	0.05	1,659 (10.7%)	1,657 (10.7%)	0.00
Antidepressants	4,901 (19.1%)	4,803 (21.9%)	-0.07	3,110 (20.1%)	3,167 (20.5%)	-0.01
Anxiolytics/hypnotics	1,192 (4.7%)	1,183 (5.4%)	-0.03	770 (5.0%)	786 (5.1%)	0.00
Benzodiazepines	1,898 (7.4%)	1,697 (7.8%)	-0.02	1,193 (7.7%)	1,177 (7.6%)	0.00
Antipsychotics	631 (2.5%)	538 (2.5%)	0.00	377 (2.4%)	381 (2.5%)	-0.01
Antiparkinsonian medications	372 (1.5%)	393 (1.8%)	-0.02	221 (1.4%)	264 (1.7%)	-0.02
Dementia medications	408 (1.6%)	145 (0.7%)	0.08	172 (1.1%)	112 (0.7%)	0.04
Measures of health care use						
Internist; n (%)	23,517 (91.7%)	19,668 (89.8%)	0.07	14,015 (90.6%)	14,050 (90.8%)	-0.01
Internist (-30 days to CED); n (%)	20,170 (78.7%)	16,206 (74.0%)	0.11	11,885 (76.8%)	11,899 (76.9%)	0.00

© 2023 D'Andrea E et al. *JAMA Internal Medicine.*

Internist (-180 days to -31 days before CED); n (%)	17,893 (69.8%)	15,293 (69.8%)	0.00	10,622 (68.7%)	10,732 (69.4%)	-0.02
Internist (Number of visits)						
...mean (sd)	10.55 (15.03)	9.28 (14.05)	0.09	9.61 (13.61)	9.61 (14.26)	0.00
Endocrinologist; n (%)	2,023 (7.9%)	3,385 (15.5%)	-0.24	1,454 (9.4%)	1,527 (9.9%)	-0.02
Endocrinologist (-30 days to CED); n (%)	1,581 (6.2%)	2,809 (12.8%)	-0.23	1,179 (7.6%)	1,183 (7.6%)	0.00
Endocrinologist (-180 days to -31 days before CED); n (%)	1,004 (3.9%)	1,829 (8.4%)	-0.19	710 (4.6%)	830 (5.4%)	-0.04
Endocrinologist (Number of visits)						
...mean (sd)	0.28 (1.45)	0.56 (1.97)	-0.16	0.34 (1.60)	0.34 (1.51)	0.00
Cardiologist; n (%)	4,386 (17.1%)	3,793 (17.3%)	-0.01	2,535 (16.4%)	2,513 (16.2%)	0.01
Cardiologist (-30 days to CED); n (%)	1,716 (6.7%)	1,492 (6.8%)	0.00	970 (6.3%)	956 (6.2%)	0.00
Cardiologist (-180 days to -31 days before CED); n (%)	3,504 (13.7%)	3,085 (14.1%)	-0.01	2,036 (13.2%)	2,045 (13.2%)	0.00
Cardiologist (Number of visits)						
...mean (sd)	0.80 (2.83)	0.86 (3.14)	-0.02	0.76 (2.75)	0.77 (3.00)	0.00
Electrocardiogram; n (%)	6,654 (26.0%)	5,341 (24.4%)	0.04	3,816 (24.7%)	3,714 (24.0%)	0.02
Electrocardiogram (Number of tests)						
...mean (sd)	0.47 (1.23)	0.47 (1.23)	0.00	0.44 (1.07)	0.45 (1.21)	-0.01
ECG & Other cardiac imaging; n (%)	2,210 (8.6%)	1,908 (8.7%)	0.00	1,233 (8.0%)	1,222 (7.9%)	0.00
ECG & Other cardiac imaging (Number of tests)						
...mean (sd)	0.29 (2.19)	0.35 (2.41)	-0.03	0.27 (2.00)	0.29 (2.11)	-0.01
Cardiovascular stress test; n (%)	895 (3.5%)	803 (3.7%)	-0.01	552 (3.6%)	491 (3.2%)	0.02
HbA1c test order (Number of tests)						
...mean (sd)	1.44 (0.81)	1.47 (0.80)	-0.04	1.44 (0.79)	1.44 (0.79)	0.00
Glucose test and monitoring; n (%)	6,517 (25.4%)	5,540 (25.3%)	0.00	3,743 (24.2%)	3,718 (24.0%)	0.00
Glucose test and monitoring (Number of tests)						
...mean (sd)	0.55 (1.39)	0.53 (1.36)	0.01	0.50 (1.21)	0.50 (1.36)	0.00
Microalbuminuria/proteinuria test order; n (%)	13,600 (53.1%)	12,002 (54.8%)	-0.03	8,286 (53.6%)	8,294 (53.6%)	0.00
Microalbuminuria/proteinuria test order (Number of tests)						
...mean (sd)	0.65 (0.74)	0.67 (0.71)	-0.03	0.65 (0.71)	0.65 (0.71)	0.00
Metabolic or renal/creatinine panel test order; n (%)	20,511 (80.0%)	17,229 (78.7%)	0.03	12,213 (79.0%)	12,129 (78.4%)	0.01
Metabolic or renal/creatinine panel test order (Number of tests)						
...mean (sd)	1.44 (1.38)	1.39 (1.28)	0.04	1.36 (1.23)	1.37 (1.27)	-0.01
Lipid test order; n (%)	21,774 (84.9%)	18,864 (86.2%)	-0.04	13,250 (85.7%)	13,278 (85.9%)	-0.01
Lipid test order (Number of tests)						
...mean (sd)	1.21 (0.86)	1.22 (0.83)	-0.01	1.20 (0.82)	1.20 (0.83)	0.00
Uric acid test order; n (%)	2,109 (8.2%)	1,551 (7.1%)	0.04	1,091 (7.1%)	1,131 (7.3%)	-0.01
Vitamin D test order; n (%)	6,789 (26.5%)	5,400 (24.7%)	0.04	3,968 (25.7%)	3,809 (24.6%)	0.03
PTH test order; n (%)	1,194 (4.7%)	762 (3.5%)	0.06	585 (3.8%)	564 (3.6%)	0.01
Hospitalization event (Number of)						
...mean (sd)	0.06 (0.28)	0.05 (0.27)	0.04	0.05 (0.25)	0.05 (0.25)	0.00
LOS (-180 days to -31 days before CED)						
...mean (sd)	0.15 (1.26)	0.18 (1.52)	-0.02	0.15 (1.36)	0.15 (1.26)	0.00
LOS (-30 days to CED)						
...mean (sd)	0.09 (0.86)	0.05 (0.60)	0.05	0.05 (0.54)	0.06 (0.65)	-0.02
ED event (Number of)						
...mean (sd)	0.20 (0.80)	0.14 (0.67)	0.08	0.15 (0.61)	0.15 (0.72)	0.00

© 2023 D'Andrea E et al. *JAMA Internal Medicine.*

Distinct medications ALL						
...mean (sd)	9.13 (4.90)	9.59 (5.07)	-0.09	9.15 (4.90)	9.16 (4.90)	0.00
Distinct medications all BRAND						
...mean (sd)	2.01 (1.33)	2.28 (1.48)	-0.19	2.03 (1.33)	2.05 (1.38)	-0.01
Cost pharmacy OOP ($)						
...mean (sd)	320.72 (383.46)	382.46 (419.49)	-0.15	334.12 (421.01)	337.07 (356.06)	-0.01
Flu vaccine; n (%)	4,304 (16.8%)	3,810 (17.4%)	-0.02	2,545 (16.5%)	2,635 (17.0%)	-0.01
Pneumococcal vaccine; n (%)	1,442 (5.6%)	1,169 (5.3%)	0.01	875 (5.7%)	869 (5.6%)	0.00
Breast mammography or MRI; n (%)	2,535 (9.9%)	2,172 (9.9%)	0.00	1,531 (9.9%)	1,534 (9.9%)	0.00
Prostate DRE or PSA; n (%)	5,000 (19.5%)	4,272 (19.5%)	0.00	3,055 (19.8%)	3,025 (19.6%)	0.01
Colonoscopy; n (%)	802 (3.1%)	661 (3.0%)	0.01	491 (3.2%)	461 (3.0%)	0.01
Pap smear; n (%)	990 (3.9%)	916 (4.2%)	-0.02	594 (3.8%)	634 (4.1%)	-0.02
Bone mineral density; n (%)	627 (2.4%)	429 (2.0%)	0.03	308 (2.0%)	318 (2.1%)	-0.01
Laboratory results						
Lab value HbA1c (%)						
...mean (sd)	10.63 (1.48)	10.61 (1.41)	0.01	10.63 (1.44)	10.63 (1.42)	0.00
...median [IQR]	10.30 [9.50, 11.50]	10.30 [9.50, 11.40]	-	10.30 [9.50, 11.50]	10.30 [9.50, 11.45]	-
eGFR (w/o race)						
...mean (sd)	80.06 (25.34)	84.03 (24.47)	-0.16	83.16 (24.53)	83.22 (24.44)	0.00
...median [IQR]	84.51 [62.95, 99.03]	88.84 [69.14, 101.8]	-	87.82 [67.75, 100.92]	87.87 [67.97, 101.09]	-
Lab result number - Creatinine						
...mean (sd)	1.05 (1.50)	1.05 (1.62)	0.00	1.04 (1.69)	1.06 (1.79)	-0.01
...median [IQR]	0.89 [0.74, 1.09]	0.87 [0.73, 1.05]	-	0.87 [0.73, 1.05]	0.87 [0.73, 1.05]	-
Lab result number - UACR						
...mean (sd)	183.21 (628.21)	177.96 (691.77)	0.01	165.19 (586.36)	176.54 (716.55)	-0.02
...Missing; n (%)	15,835 (61.8%)	13,269 (60.6%)	0.02	9,406 (60.8%)	9,646 (62.4%)	-0.03
Lab result number - Total cholesterol						
...mean (sd)	191.37 (54.82)	189.79 (57.21)	0.03	191.45 (55.48)	191.29 (56.87)	0.00
...Missing; n (%)	3,550 (13.8%)	3,092 (14.1%)	-0.01	2,068 (13.4%)	2,179 (14.1%)	-0.02
Lab result number - LDL cholesterol						
...mean (sd)	106.16 (45.91)	104.84 (47.12)	0.03	106.32 (45.88)	106.01 (47.30)	0.01
...Missing; n (%)	5,139 (20.0%)	4,555 (20.8%)	-0.02	3,083 (19.9%)	3,207 (20.7%)	-0.02
Lab result number - HDL cholesterol						
...mean (sd)	46.01 (18.35)	45.61 (20.62)	0.02	45.49 (18.69)	45.75 (19.47)	-0.01
...Missing; n (%)	3,818 (14.9%)	3,401 (15.5%)	-0.02	2,261 (14.6%)	2,404 (15.5%)	-0.03
Lab result number - Triglycerides						
...mean (sd)	233.12 (194.16)	250.23 (216.65)	-0.08	239.45 (202.52)	245.76 (211.18)	-0.03
...Missing; n (%)	3,601 (14.0%)	3,022 (13.8%)	0.01	2,099 (13.6%)	2,156 (13.9%)	-0.01
Lab result value - Glucose						
...mean (sd)	256.88 (112.87)	260.92 (135.19)	-0.03	259.05 (117.70)	259.39 (126.65)	0.00
...Missing; n (%)	712 (2.8%)	566 (2.6%)	0.01	431 (2.8%)	396 (2.6%)	0.01

Abbreviations. St. Diff., standardized difference; SD, standard deviation; GLP-1 RA, Glucagon-like peptide-1 receptor agonists; MI, myocardial infarction; TIA, transient ischemic attack; peripheral artery disease; NASH/NAFLD, Nonalcoholic steatohepatitis/Nonalcoholic fatty liver disease; COPD, chronic obstructive pulmonary disease; CKD, chronic kidney disease; ARBs, Angiotensin II Receptor Blockers; ACEis, Angiotensin Converting Enzyme inhibitors; MRA, mineralocorticoid receptor antagonist; PCSK9, Proprotein convertase subtilisin/kexin type 9; NSAIDs, nonsteroidal anti-inflammatory drugs; MRI, magnetic resonance imaging; DRE, digital rectal examination; PSA, prostate-specific antigen; ED, emergency department, LOS, length of stay; ECG, electrocardiogram; eGFR, estimated glomerular filtration rate; uACR, urine albumin-creatinine ratio; LDL, low-density lipoprotein; HDL, high-density lipoprotein.

© 2023 D’Andrea E et al. *JAMA Internal Medicine.*

eFigure 2. Propensity score distributions of SGLT2i and DPP4i initiators in the overall population.

A. Pre-matching propensity score overlap | **B.** Post-matching propensity score overlap

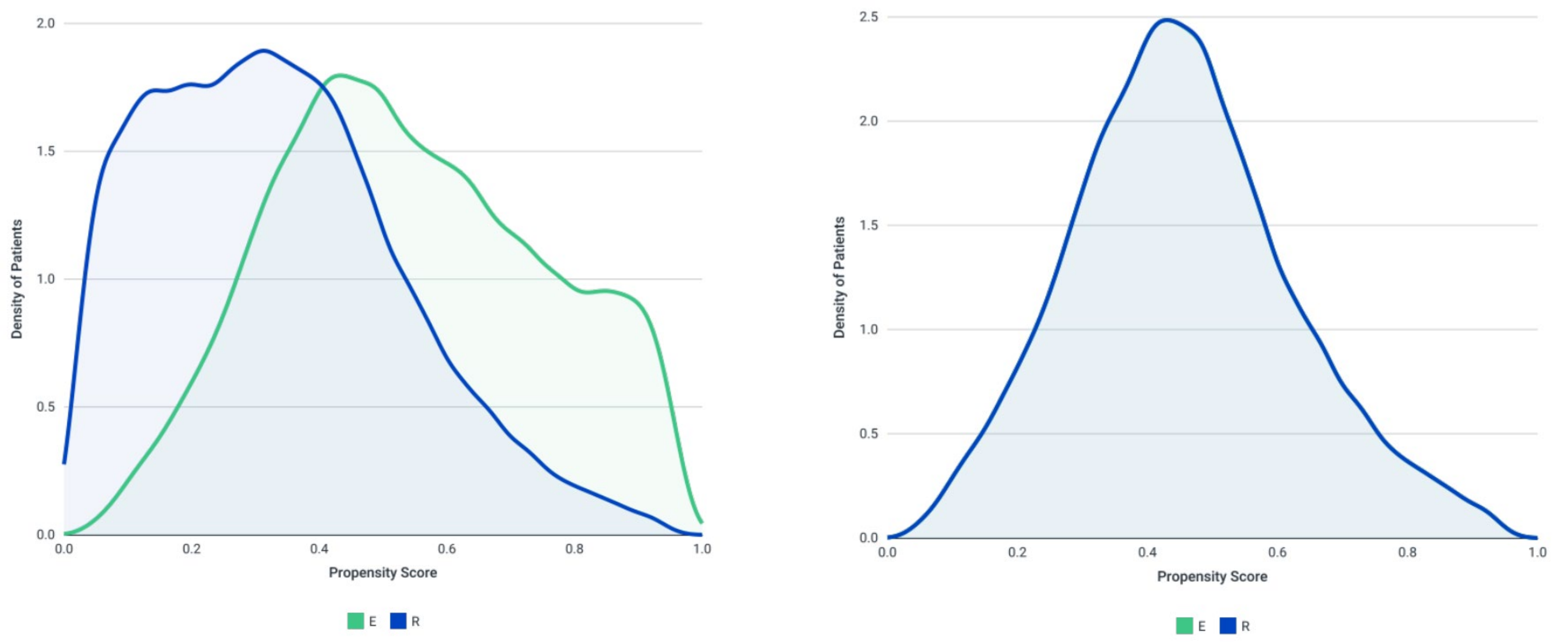

Legend E = SGLT2 inhibitors; R = DPP4 inhibitors.

Caption The figure displays the propensity score distribution overlap prior to (**A**) and after (**B**) matching. In both graphs (**A** and **B**), the x-axis shows the range of propensity scores (0 to 1), and the y-axis shows the density of patients. Patients in the DPP4i group (blue) have lower propensity scores, while those in the SGLT2i group (green) have higher propensity scores (**A**). The overlapped proportion of patients in **B** is included in the propensity score matched analysis.

© 2023 D’Andrea E et al. *JAMA Internal Medicine.*

eTable 7. Follow-up and censoring reasons after PS-matching (primary outcomes)

	Patients with HbA1c < 7.5%			Patients with HbA1c 7.5-9%			Patients with HbA1c > 9%		
	**Overall N = 24,052**	**DPP-4 inhibitor N = 12,026**	**SGLT2 inhibitor N = 12,026**	**Overall N = 32,290**	**DPP-4 inhibitor N = 16,145**	**SGLT2 inhibitor N = 16,145**	**Overall N = 30,932**	**DPP-4 inhibitor N = 15,446**	**SGLT2 inhibitor N = 15,446**
**Outcome: Modified MACE**									
Follow-up, median (IQR), days	133 [68, 300]	131 [70, 298]	136 [64, 303]	134 [66, 302]	136 [77, 299]	133 [58, 305]	118 [58, 262]	118 [58, 248]	119 [58, 277]
Follow-up, mean (SD), days	250 [296.4]	248 [291.6]	252 [301.2]	251 [297.1]	249 [289.7]	252 [304.3]	220 [266.2]	212 [253.0]	229 [278.6]
Censoring reasons, %									
Outcome occurrence	255 (1.06%)	139 (1.16%)	116 (0.96%)	383 (1.19%)	205 (1.27%)	178 (1.10%)	420 (1.36%)	222 (1.44%)	198 (1.28%)
Treatment switching	1,052 (4.37%)	544 (4.52%)	508 (4.22%)	1,960 (6.07%)	1,166 (7.22%)	794 (4.92%)	2,029 (6.56%)	1,272 (8.22%)	757 (4.89%)
Treatment discontinuation	15,061 (62.62%)	7,620 (63.36%)	7,441 (61.87%)	20,365 (63.07%)	10,190 (63.12%)	10,175 (63.02%)	20,044 (64.80%)	10,031 (64.86%)	10,013 (64.74%)
End of available data - 06/30/2021	4,179 (17.37%)	2,041 (16.97%)	2,138 (17.78%)	4,865 (15.07%)	2,342 (14.51%)	2,523 (15.63%)	3,590 (11.61%)	1,715 (11.09%)	1,875 (12.12%)
Disenrollment	3,464 (14.40%)	1,659 (13.80%)	1,805 (15.01%)	4,671 (14.47%)	2,221 (13.76%)	2,450 (15.17%)	4,808 (15.54%)	2,207 (14.27%)	2,601 (16.82%)
Initiation of SGLT-2i/DPP4i combinations	41 (0.17%)	23 (0.19%)	18 (0.15%)	46 (0.14%)	21 (0.13%)	25 (0.15%)	41 (0.13%)	19 (0.12%)	22 (0.14%)
**Outcome: Hospitalization for HF**									
Follow-up, median (IQR), days	134 [68, 301]	131 [70, 298]	136 [64, 304]	135 [66, 302]	137 [78, 300]	133 [58, 307]	118 [58, 263]	118 [58, 249]	119 [58, 279]
Follow-up, mean (SD), days	251 [297.1]	248 [291.9]	253 [302.2]	252 [297.9]	250 [290.4]	253 [305.1]	222 [267.9]	213 [254.7]	230 [280.1]
Censoring reasons, %									
Outcome occurrence	112 (0.47%)	75 (0.62%)	37 (0.31%)	674 (1.4%)	84 (0.52%)	37 (0.23%)	103 (0.33%)	69 (0.45%)	34 (0.22%)
All-cause mortality	96 (0.40%)	57 (0.47%)	39 (0.32%)	155 (0.48%)	81 (0.50%)	74 (0.46%)	148 (0.48%)	83 (0.54%)	65 (0.42%)
Treatment switching	1,054 (4.38%)	545 (4.53%)	509 (4.23%)	1.963 (6.08)	1,171 (7.25%)	792 (4.91%)	2,037 (6.59%)	1,273 (8.23%)	764 (4.94%)
Treatment discontinuation	15,092 (62.75%)	7,623 (63.39%)	7,469 (62.11%)	20,432 (63.28%)	10,209 (63.23%)	10,223 (63.32%)	20,165 (65.19%)	10,085 (65.21%)	10,080 (65.18%)
End of available data - 06/30/2021	4,191 (17.42%)	2,047 (17.02%)	2,144 (17.83%)	4,888 (15.14%)	2,356 (14.59%)	2,532 (15.68%)	3,606 (11.66%)	1,721 (11.13%)	1,885 (12.19%)
Disenrollment	3,467 (14.41%)	1,657 (13.78%)	1,810 (15.05%)	4,684 (14.51%)	2,222 (13.76%)	2,462 (15.25%)	4,831 (15.62%)	2,215 (14.32%)	2,616 (16.91%)
Initiation of SGLT-2i/DPP4i combinations	40 (0.17%)	22 (0.18%)	18 (0.15%)	47 (0.15%)	22 (0.15%)	25 (0.15%)	42 (0.14%)	22 (0.14%)	20 (0.13%)

Abbreviations: MACE, major adverse cardiovascular events; IQR, interquartile range; SD, standard deviation; HbA1c, HbA1c, glycated hemoglobin; DPP-4, dipeptidyl peptidase 4; SGLT2, Sodium-Glucose Cotransporter-2. Follow-up and censoring are strictly dependent on the outcome of interest. The table reports follow-up and censoring information specific to the primary outcomes. Follow-up and censoring reasons specific to secondary outcomes may include minor variations.

© 2023 D'Andrea E et al. *JAMA Internal Medicine.*

eFigure 3. Cumulative incidence of modified MACE and HHF comparing 1:1 propensity score–matched patients initiating SGLT2 inhibitor vs. DPP-4 inhibitor therapy stratified by HbA_{1c} levels.

A. Subgroup of patients with HbA1c < 7.5%

A1. Modified MACE (i.e., myocardial infarction, stroke, all-cause mortality) in patients with HbA1c < 7.5%

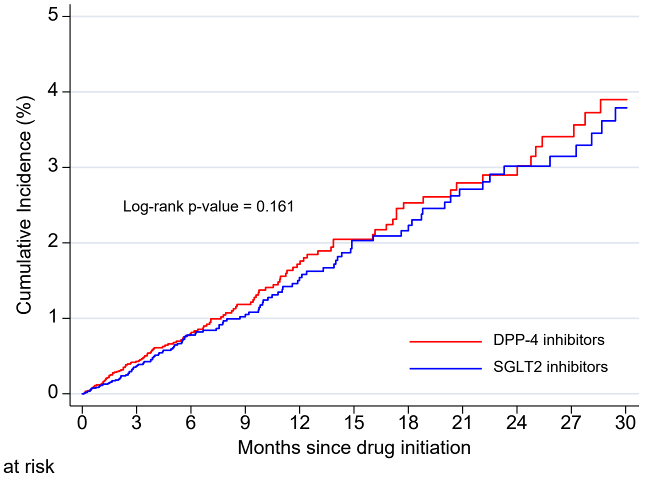

Number at risk

	0	3	6	9	12	15	18	21	24	27	30
DPP-4 inhibitors	12026	8356	4963	3318	2343	1729	1285	1008	807	631	497
SGLT2 inhibitors	12026	8219	4990	3367	2427	1790	1370	1091	849	674	534

A2. Hospitalization for HF in patients with HbA1c < 7.5%

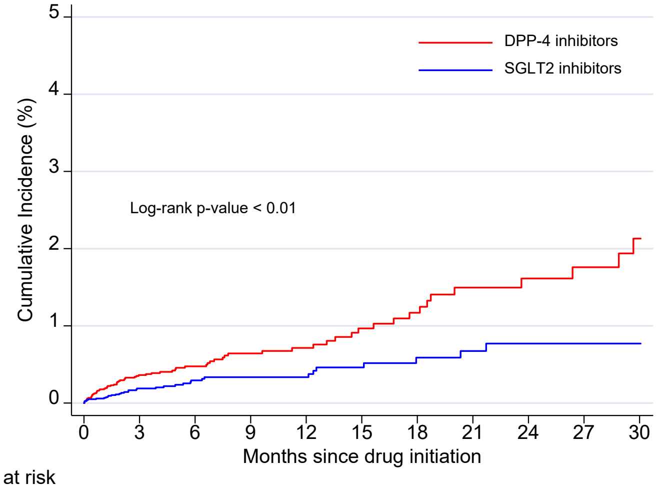

Number at risk

	0	3	6	9	12	15	18	21	24	27	30
DPP-4 inhibitors	12026	8354	4977	3326	2355	1736	1291	1014	812	634	496
SGLT2 inhibitors	12026	8227	5001	3377	2440	1804	1380	1101	855	677	538

B. Subgroup of patients with HbA1c 7.5-9%

B1. Modified MACE (i.e., myocardial infarction, stroke, all-cause mortality) in patients with HbA1c 7.5-9%

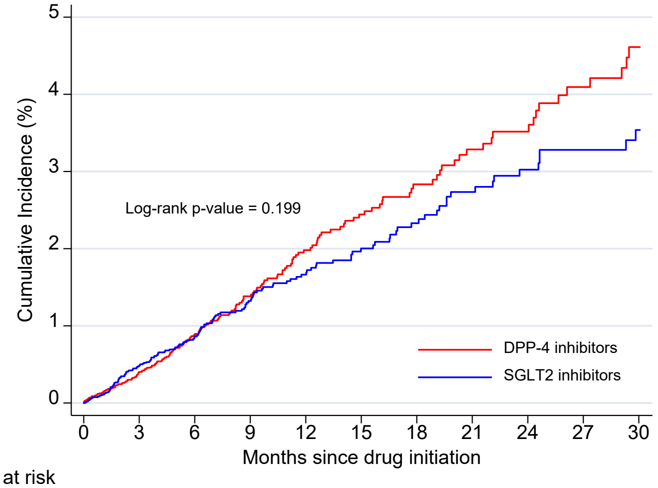

Number at risk

	0	3	6	9	12	15	18	21	24	27	30
DPP-4 inhibitors	16145	11312	6798	4481	3163	2331	1739	1345	1078	848	686
SGLT2 inhibitors	16145	10850	6610	4502	3340	2442	1851	1467	1181	933	726

B2. Hospitalization for HF in patients with HbA1c 7.5%-9%

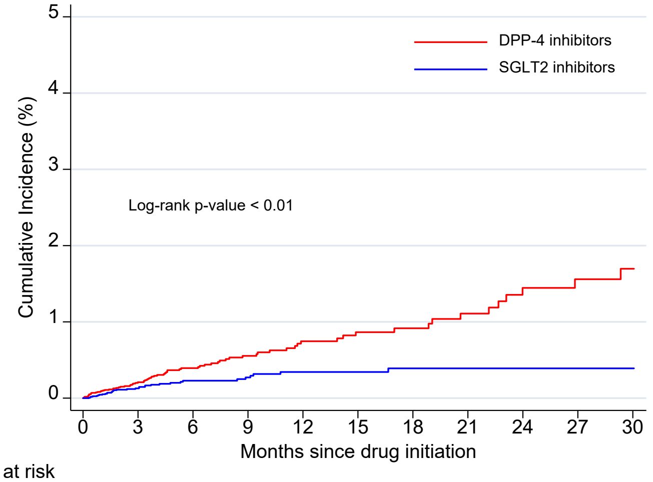

Number at risk

	0	3	6	9	12	15	18	21	24	27	30
DPP-4 inhibitors	16145	11317	6811	4497	3173	2345	1755	1356	1083	855	693
SGLT2 inhibitors	16145	10873	6632	4528	3360	2461	1867	1482	1192	942	734

C. Subgroup of patients with HbA1c > 9%

C1. Modified MACE (i.e., myocardial infarction, stroke, all-cause mortality) in patients with HbA1c > 9%

C2. Hospitalization for HF in patients with HbA1c > 9%

© 2023 D'Andrea E et al. *JAMA Internal Medicine.*

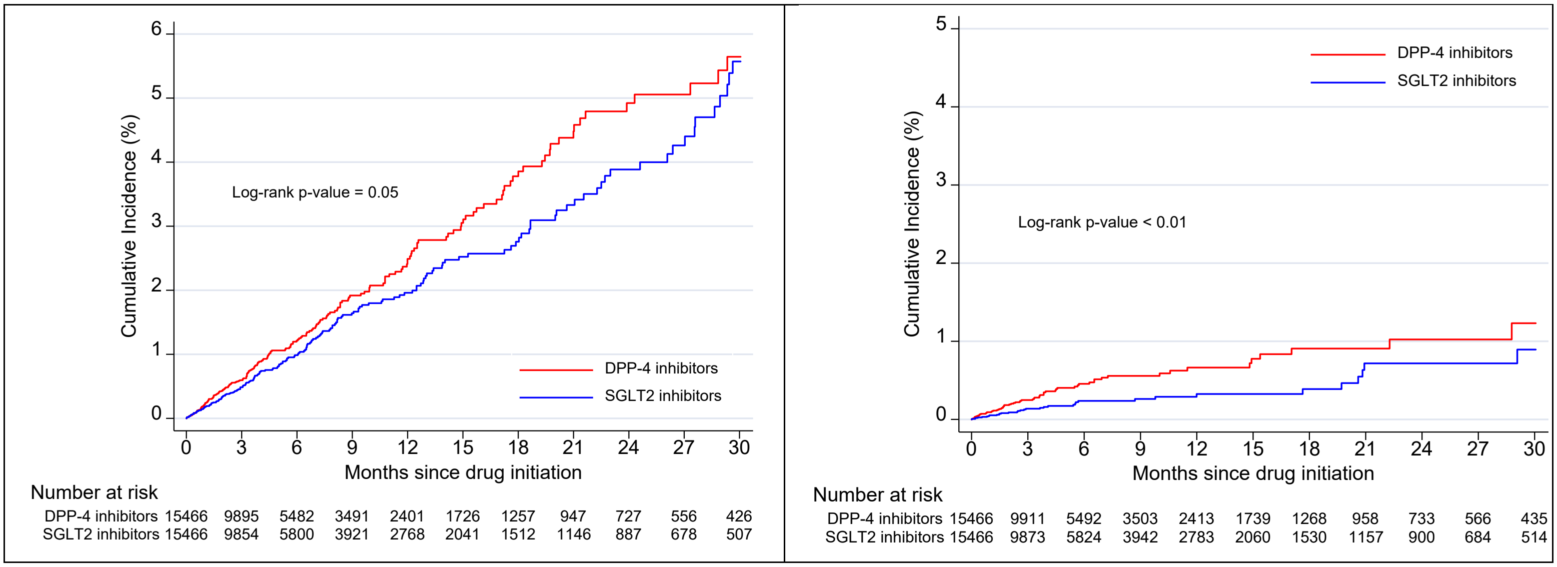

© 2023 D'Andrea E et al. *JAMA Internal Medicine.*

eTable 8. Time-limited "intent-to-treat" analysis and secondary analyses of the primary effectiveness outcomes in 1:1 propensity score-matched patients initiating SGLT2 inhibitor vs. DPP-4 inhibitor therapy and stratified by HbA_{1c} levels.

Sensitivity analyses for effectiveness outcomes	Events, N (IR/1,000 PY)	Events, N (IR/1,000 PY)	HR (95% CI)	RD/1,000 PY (95% CI)
1. Time-limited "intent-to-treat" analysis	**SGLT2 inhibitor**	**DPP-4 inhibitor**	**SGLT2 inhibitor vs DPP-4 inhibitor**	
**Modified MACE***				
Subgroup HbA1c < 7.5% (N = 24,078)	150 (15.77)	185 (19.35)	0.82 (0.66, 1.01)	-3.57 (-7.33, 0.19)
Subgroup HbA1c 7.5-9% (N = 32,356)	266 (20.48)	249 (18.94)	1.08 (0.91, 1.29)	1.54 (-1.86, 4.94)
Subgroup HbA1c > 9% (N = 31,052)	278 (22.61)	316 (25.40)	0.89 (0.76, 1.05)	-2.79 (-6.65, 1.08)
Overall population (N = 87,486)	694 (19.95)	750 (21.33)	0.92 (0.82, 1.01)	-1.37 (-3.48, 0.75)
**Hospitalization for HF**				
Subgroup HbA1c < 7.5% (N = 24,078)	46 (4.82)	92 (9.62)	0.50 (0.35, 0.71)	-4.80 (-7.21, -2.39)
Subgroup HbA1c 7.5-9% (N = 32,356)	58 (4.45)	93 (7.06)	0.63 (0.45, 0.87)	-2.61 (-4.45, -0.78)
Subgroup HbA1c > 9% (N = 31,052)	67 (5.43)	113 (9.06)	0.60 (0.44, 0.81)	-3.63 (-5.74, -1.51)
Overall population (N = 87,486)	171 (4.89)	298 (8.46)	0.57 (0.46, 0.68)	-3.48 (-4.69, -2.28)
2. Secondary analysis restricted to patients with concurrent use of metformin and no use of insulin at baseline	**SGLT2 inhibitor**	**DPP-4 inhibitor**	**SGLT2 inhibitor vs DPP-4 inhibitor**	
**Modified MACE***				
Subgroup HbA1c < 7.5% (N = 10,948)	50 (12.16)	60 (14.59)	0.83 (0.57, 1.21)	-2.43 (-7.43, 2.56)
Subgroup HbA1c 7.5-9% (N = 16,066)	74 (12.51)	76 (12.75)	0.99 (0.72, 1.36)	-0.24 (-4.28, 3.80)
Subgroup HbA1c > 9% (N = 11,696)	65 (16.28)	65 (17.16)	0.95 (0.67, 1.33)	-0.88 (-6.63, 4.87)
Overall population (N = 38,710)	189 (13.48)	201 (14.50)	0.92 (0.74, 1.11)	-1.05 (-3.81, 1.70)
**Hospitalization for HF**				
Subgroup HbA1c < 7.5% (N = 10,948)	8 (1.94)	29 (7.04)	0.27 (0.12, 0.59)	-5.10 (-8.00, -2.21)
Subgroup HbA1c 7.5-9% (N = 16,066)	14 (2.36)	14 (2.34)	1.00 (0.48, 2.11)	0.02 (-1.72, 1.76)
Subgroup HbA1c > 9% (N = 11,696)	4 (0.99)	10 (2.63)	0.39 (0.12, 1.25)	-1.63 (-3.53, 0.27)
Overall population (N = 38,710)	26 (1.85)	53 (3.81)	0.34 (0.13, 0.55)	-1.45 (-2.62, -0.28)
3. Secondary analysis restricted to patients initiating a SGLT2 inhibitor or sitagliptin	**SGLT2 inhibitor**	**Sitagliptin**	**SGLT2 inhibitor vs Sitagliptin**	
**Hospitalization for HF**				
Subgroup HbA1c < 7.5% (N = 19,512)	37 (5.11)	61 (9.09)	0.56 (0.37, 0.85)	-3.97 (-6.79, -1.16)
Subgroup HbA1c 7.5-9% (N = 25,610)	34 (3.66)	71 (8.09)	0.45 (0.30, 0.68)	-4.43 (-6.68, -2.18)
Subgroup HbA1c > 9% (N = 21,576)	25 (3.53)	73 (11.59)	0.30 (0.19, 0.48)	-8.06 (-11.06, -5.07)
Overall population (N = 66,698)	96 (4.06)	205 (9.41)	0.40 (0.29, 0.50)	-5.23 (-6.74, -3.71)

Abbreviations: DPP-4, dipeptidyl peptidase 4; SGLT2, Sodium-Glucose Cotransporter-2; N, number; IR, incidence rate; PY, person-years; RD, rate difference; HR, hazard ratio; CI, confidence interval; HF, heart failure; MACE, major adverse cardiac events (i.e., myocardial infarction, stroke, and all-cause mortality).

© 2023 D'Andrea E et al. *JAMA Internal Medicine.*

eTable 9. Number of events, incidence rates, relative and absolute estimates for sensitivity analysis of incident safety outcomes in 1:1 PS-matched initiators of SGLT2 inhibitors vs. DPP-4 inhibitors

	SGLT2 inhibitor			DPP-4 inhibitor			SGLT2 inhibitor vs DPP-4 inhibitor - relative scale -	SGLT2 inhibitor vs DPP-4 inhibitor - absolute scale -
	N.	N. events	IR/1,000 PY	N.	N. events	IR/1,000 PY	HR (95% CI)	RD/1,000 PY (95% CI)
**Hypovolemia**								
Subgroup HbA1c < 7.5%	11,044	74	9.96	11,044	66	8.7	0.87 (0.63, 1.22)	-1.25 (-4.34, 1.84)
Subgroup HbA1c 7.5-9%	14,748	86	8.59	14,748	79	7.66	0.91 (0.67, 1.23)	-0.93 (-3.41, 1.55)
Subgroup HbA1c > 9%	14,006	79	9.69	14,006	76	8.56	0.90 (0.66, 1.24)	-1.13 (-4.01, 1.75)
**Non-vertebral fractures**								
Subgroup HbA1c < 7.5%	11,044	31	4.08	11,044	36	4.83	0.85 (0.52, 1.37)	-0.75 (-2.89, 1.38)
Subgroup HbA1c 7.5-9%	14,748	35	3.39	14,748	42	4.19	0.81 (0.52, 1.27)	-0.68 (-2.23, 0.87)
Subgroup HbA1c > 9%	14,006	29	3.26	14,006	31	3.79	0.86 (0.52, 1.43)	-0.53 (-2.32, 1.25)
**Falls**								
Subgroup HbA1c < 7.5%	11,044	35	4.7	11,044	35	4.6	0.98 (0.61, 1.56)	-0.09 (-2.27, 2.09)
Subgroup HbA1c 7.5-9%	14,748	47	4.55	14,748	58	5.78	0.79 (0.54, 1.16)	-1.23 (-3.21, 0.75)
Subgroup HbA1c > 9%	14,006	34	3.82	14,006	39	4.77	0.81 (0.51, 1.28)	-0.95 (-2.92, 1.02)
**Genital Infections**								
Subgroup HbA1c < 7.5%	11,044	415	56.48	11,044	160	21.7	2.61 (2.17, 3.13)	34.78 (28.39, 41.17)
Subgroup HbA1c 7.5-9%	14,748	611	61.27	14,748	203	20.45	3.03 (2.59, 3.56)	40.82 (35.21, 46.43)
Subgroup HbA1c > 9%	14,006	525	60.92	14,006	246	30.41	2.06 (1.77, 2.40)	30.51 (24.06, 36.96)
**Diabetic Ketoacidosis**								
Subgroup HbA1c < 7.5%	11,044	5	0.66	11,044	3	0.4	1.64 (0.39, 6.85)	0.25 (-0.48, 0.99)
Subgroup HbA1c 7.5-9%	14,748	14	1.35	14,748	6	0.6	2.29 (0.88, 5.97)	0.76 (-0.10, 1.61)
Subgroup HbA1c > 9%	14,006	37	4.15	14,006	18	2.2	1.99 (1.13, 3.49)	1.96 (0.28, 3.64)
**Acute kidney injury**								
Subgroup HbA1c < 7.5%	11,044	138	12.5	11,044	192	17.39	0.70 (0.56, 0.87)	-4.89 (-8.18, -1.60)
Subgroup HbA1c 7.5-9%	14,748	178	17.31	14,748	259	26.15	0.66 (0.55, 0.80)	-8.85 (-12.92, -4.77)
Subgroup HbA1c > 9%	14,006	184	20.81	14,006	213	26.26	0.81 (0.66, 0.99)	-5.45 (-10.08, -0.81)

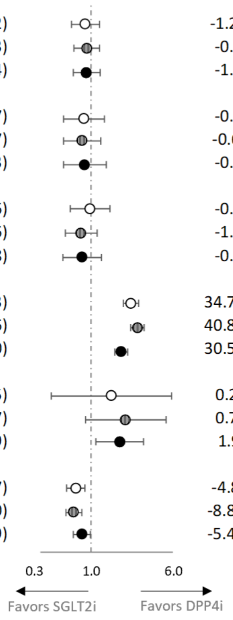

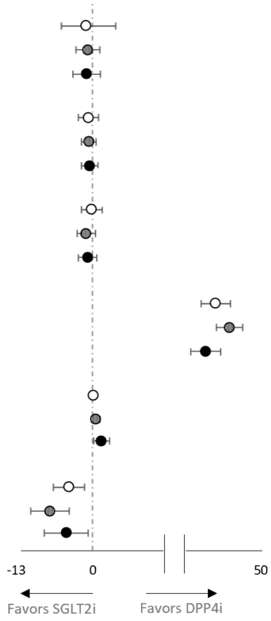

Abbreviations:DPP-4, dipeptidyl peptidase 4; SGLT2, Sodium-Glucose Cotransporter-2; N, number; IR, incidence rate; PY, person-years; RD, rate difference; HR, hazard ratio; CI, confidence interval; HbA1c, glycated hemoglobin.

P value for homogeneity is > 0.05 for all outcomes except for genital infections where p value for homogeneity is < 0.01.

© 2023 D'Andrea E et al. *JAMA Internal Medicine.*
