# Supplementary material for: Comparing Effectiveness and Safety of SGLT2 Inhibitors vs DPP-4 Inhibitors in Patients With Type 2 Diabetes and Varying Baseline HbA1c Levels
Source: JAMA Intern Med. 2023 Feb 6;183(3):242–54. doi: 10.1001/jamainternmed.2022.6664 (PMC9989905; doi:10.1001/jamainternmed.2022.6664)
Supplement: Supplement 2. — Data Sharing Statement [file jamainternmed-e226664-s002.pdf]

Data Sharing Statement

D'Andrea. Comparing Effectiveness and Safety of SGLT2 Inhibitors vs DPP-4 Inhibitors in Patients With Type 2 Diabetes and Varying Baseline HbA_{1c} Levels. *JAMA Intern Med*. Published February 06, 2023. doi:10.1001/jamainternmed.2022.6664

Data
Data available: No

Additional Information
Explanation for why data not available: Individual patient level data cannot be share with third parties based on the Data Use Agreement in place between the Division of Pharmacoepidemiology and Pharmacoeconomics of Mass General Brigham and Optum Clinformatics.
